# Supplementary material for: Novel integrated multiomics analysis reveals a key role for integrin beta-like 1 in wound scarring
Source: EMBO Rep. 2024 Nov 18;26(1):122–52. doi: 10.1038/s44319-024-00322-3 (PMC11724056; doi:10.1038/s44319-024-00322-3)
Supplement: Supplementary file 1 — Appendix [file 44319_2024_322_MOESM1_ESM.pdf]

Appendix

Novel integrated multiomics analysis reveals a key role for integrin beta-like 1 in wound scarring

Sang-Eun Kim, Ryota Noda, Yu-Chen Liu, Yukari Nakajima, Shoichiro Kameoka, Daisuke Motooka, Seiya Mizuno, Satoru Takahashi, Kento Takaya, Takehiko Murase, Kazuya Ikematsu, Katsiaryna Tratsiakova, Takahiro Motoyama, Masahiro Nakashima, Kazuo Kishi, Paul Martin, Shigeto Seno, Daisuke Okuzaki and Ryoichi Mori

Table of contents

Appendix Figure S1. Identification of cell types at skin wound sites (p. 2).
Appendix Figure S2. Subclustering of cell types at skin wound sites (p. 3).
Appendix Figure S3. Specific impacts of IL1β signaling on the F4 fibroblast subcluster (p. 4).
Appendix Figure S4. Special transcriptome analysis using the Visium platform (p. 5).
Appendix Figure S5. Gene expression at wound sites in WT and *Itgbl1*$^{-/-}$ mice (p. 6).
Appendix Figure S6. Collagen fibril morphology and skin wound healing in WT and *Itgbl1* transgenic mice (pp. 7-8).
Appendix Figure S7. Regulation of *Itgbl1* expression in MDF and HDF treated with TGFβ1 (p. 9).

Appendix Table S1. References for marker genes in macrophage subclusters (p. 10).
Appendix Table S2. References for marker genes in fibroblast subclusters (p. 11).

References (pp. 12-18)

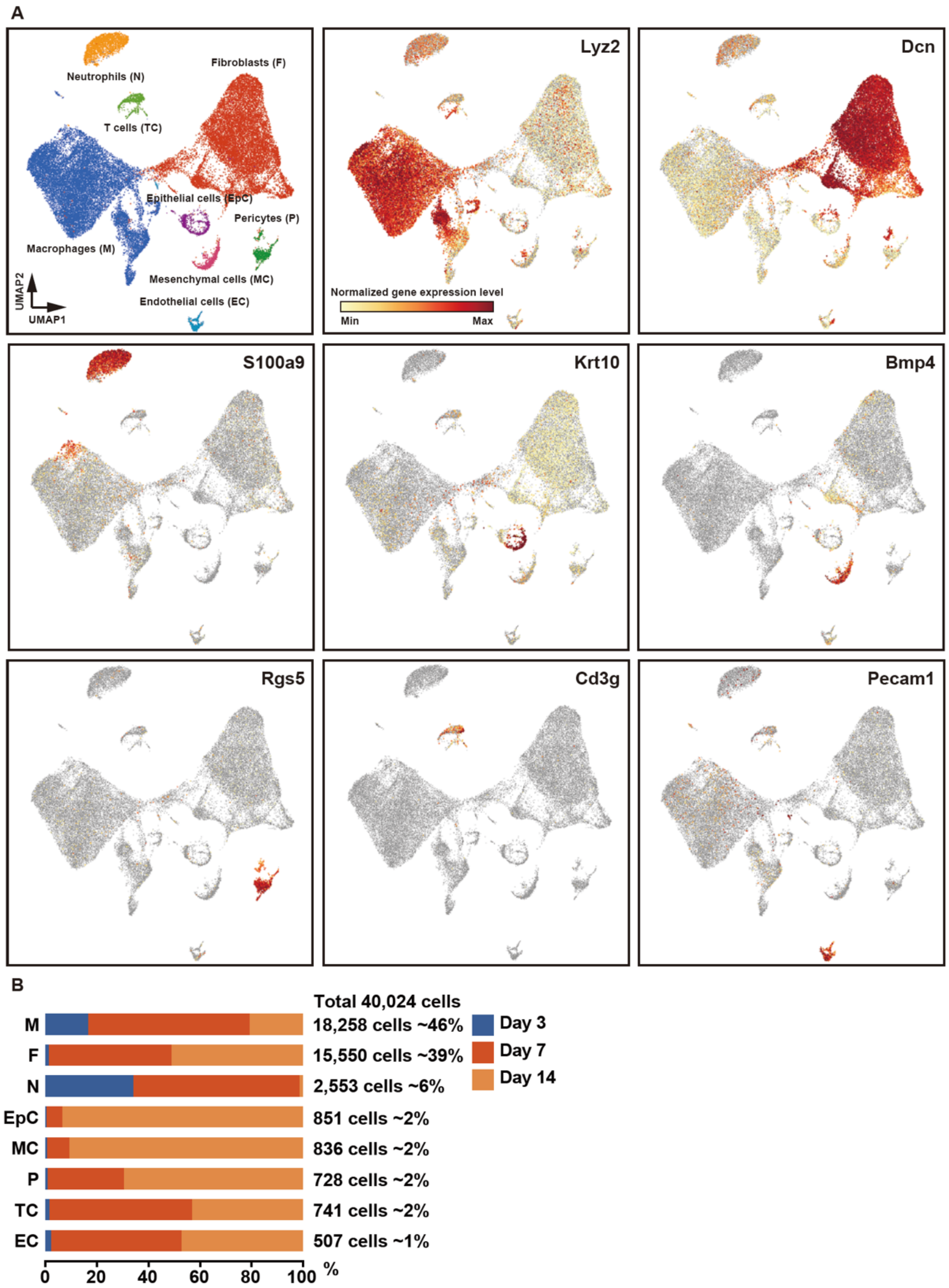

Appendix Figure S1. Identification of cell types at skin wound sites.

A. UMAP plot of 40,024 cells from integrated data collected on Days 3 (4,117 cells), 7 (21,476 cells), and 14 (14,431 cells) post skin wounding, showing each cell type expressing a characteristic marker gene. N: neutrophils (*S100a9*); TC: T cells (*Cd3g*); M: macrophages (*Lyz2*); F: fibroblasts (*Dcn*); EpC: epithelial cells (*Krt10*); MC: mesenchymal cells (*Bmp4*); P: pericytes (*Rgs5*); EC: endothelial cells (*Pecam1*).

B. Unsupervised hierarchical clustering showing relatedness of each cluster and proportions of cells on Days 3 (blue), 7 (red), and 14 (orange) post skin injury illustrated as a bar plot.

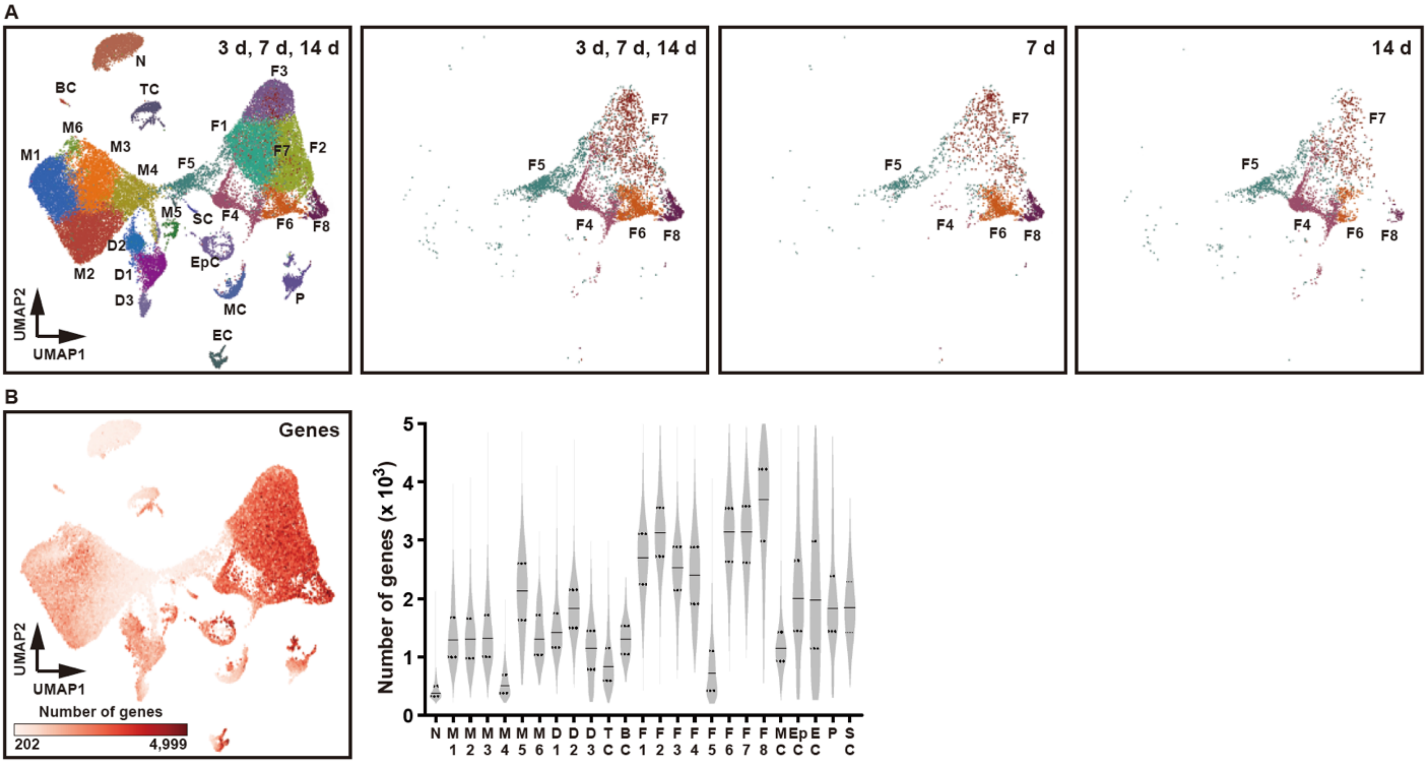

Appendix Figure S2. Subclustering of cell types at skin wound sites.

A. UMAP plot of 40,024 cells from integrated data collected on Days 3 (4,117 cells), 7 (21,476 cells), and 14 (14,431 cells) post skin wounding, showing subcluster F7 on each day.

B. UMAP plot (left) showing gene expression levels across 40,024 cells from Days 3, 7, and 14 (left panel), and violin plot (right) showing the number of genes (right panel) expressed in the different subclusters and cell types. N: neutrophils; TC: T cells; BC: B cells; M: macrophages; D: dendritic cells; F: fibroblasts; EpC: epithelial cells; MC: mesenchymal cells; P: pericytes; EC: endothelial cells; SC: Schwann cells.

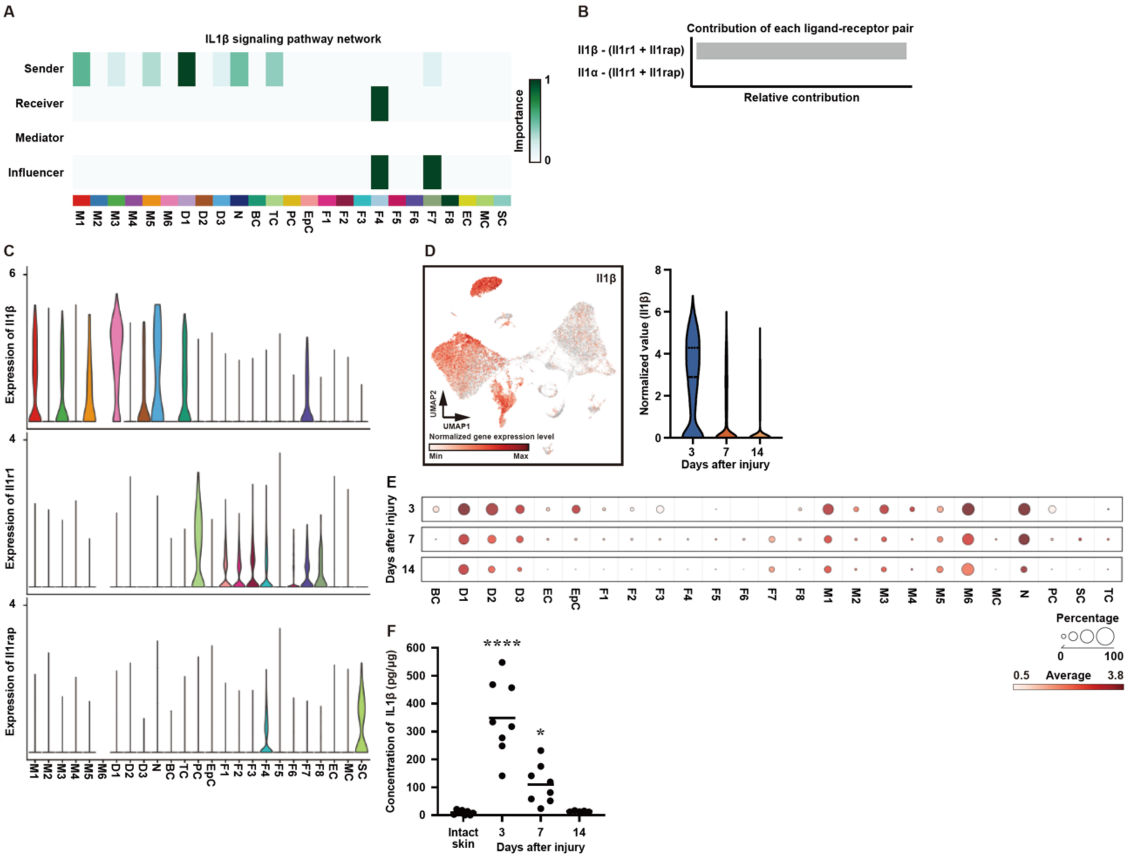

Appendix Figure S3. Specific impacts of IL1β signaling on the F4 fibroblast subcluster.

A. Visualization of the computed network centrality scores for IL1β signaling on Day 14 post injury using CellChat.

B. Analysis of the contribution of each IL1β ligand and Il1r1/Il1rap receptor pair on Day 14 post injury using CellChat.

C. Expression analysis of IL1β, Il1r1, and Il1rap on Day 14 post injury using CellChat. Note that only the F4 fibroblast subcluster expressed Il1r1 and Il1rap, which are necessary for IL1β signaling.

D. UMAP plots (left) and violin plots (right) of normalized IL1β expression levels from integrated data collected on Days 3, 7, and 14 post injury.

E. Bubble heatmap of IL1β expression in each cell subcluster.

F. IL1β protein expression at wound sites in WT mice on intact skin, Days 3, 7, and 14 post injury (intact skin, Day 3 and 7; $n = 8$, Day 14; $n = 5$).

Data information: All values represent the mean (F). One-way ANOVA followed by Dunnett's multiple comparisons test (control vs sample) was used to generate the indicated P values; $^{*}P < 0.05$, $^{**}P < 0.01$, $^{****}P < 0.0001$.

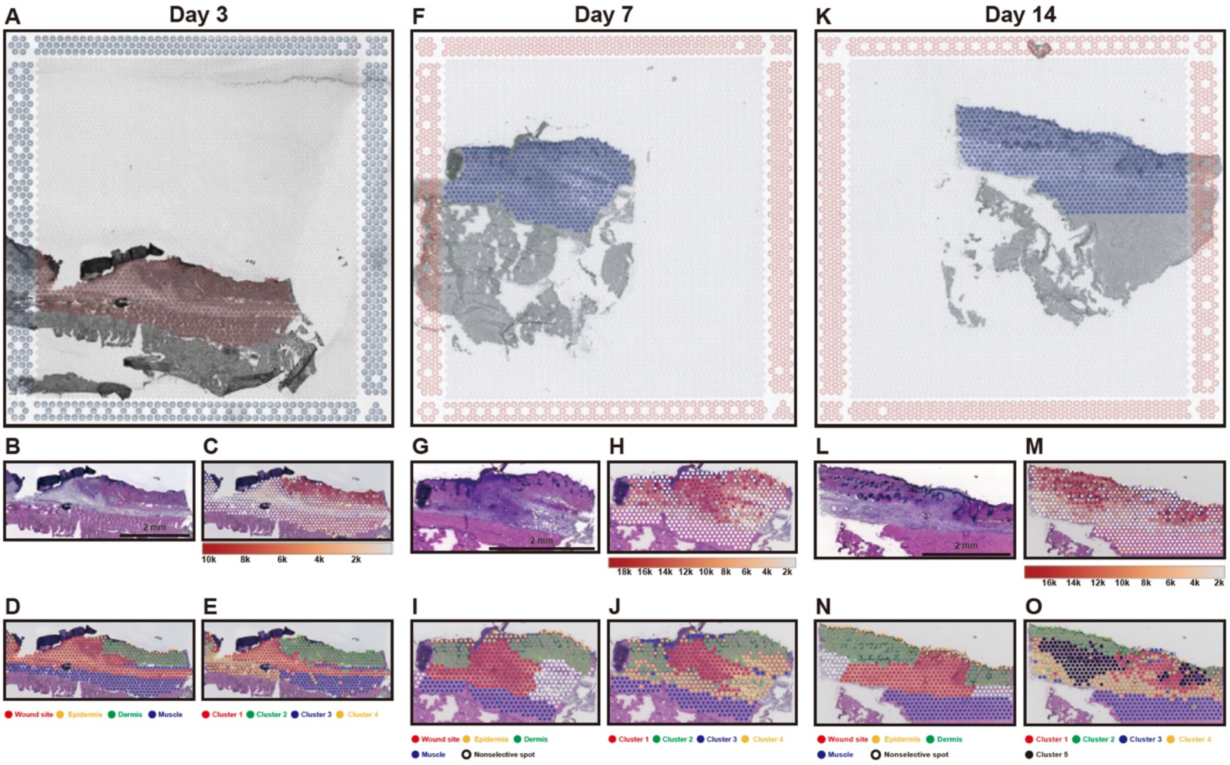

Appendix Figure S4. Special transcriptome analysis using the Visium platform. Skin wound sites on Days 3 (A–E), 7 (F–J), and 14 (K–O) post injury. (A, F, K) Original Visium photographs, with selected spots indicating the region of interest. (B, G, H) H&E staining. (C, H, M) Visualization of gene expression levels in skin wound tissue at each spot, with spot colors indicating the unique molecular identifier (UMI) count. Heat maps indicate UMI expression levels. (D, I, N) Tissue plots with spot colors indicating results of manual clustering. (E, J, O) Tissue plots with spot colors indicating results of computational clustering analysis.

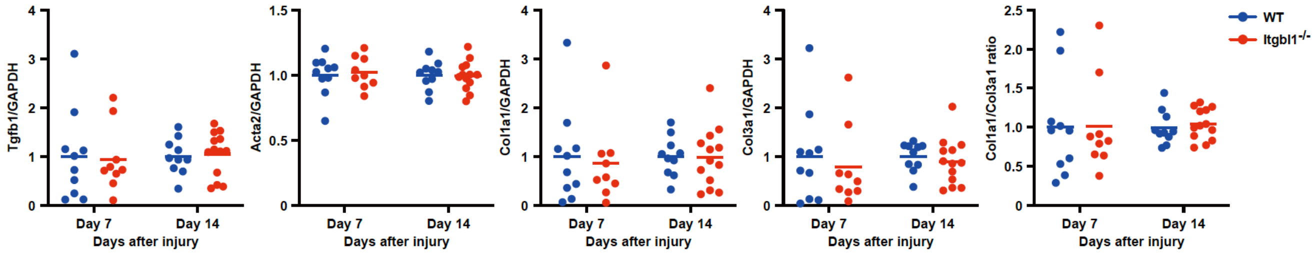

Appendix Figure S5. Gene expression at wound sites in WT and *Itgbl1*$^{-/-}$ mice.

qPCR analyses of *Tgfb1*, *Acta2*, *Co1la1*, and *Col3a1* expression patterns at skin wound sites in WT and *Itgbl1*$^{-/-}$ mice on Days 7 (WT; *n* = 10, *Itgbl1*$^{-/-}$; *n* = 9) and 14 (WT; *n* = 10, *Itgbl1*$^{-/-}$; *n* = 13) post injury.

Data information: All values represent the mean.

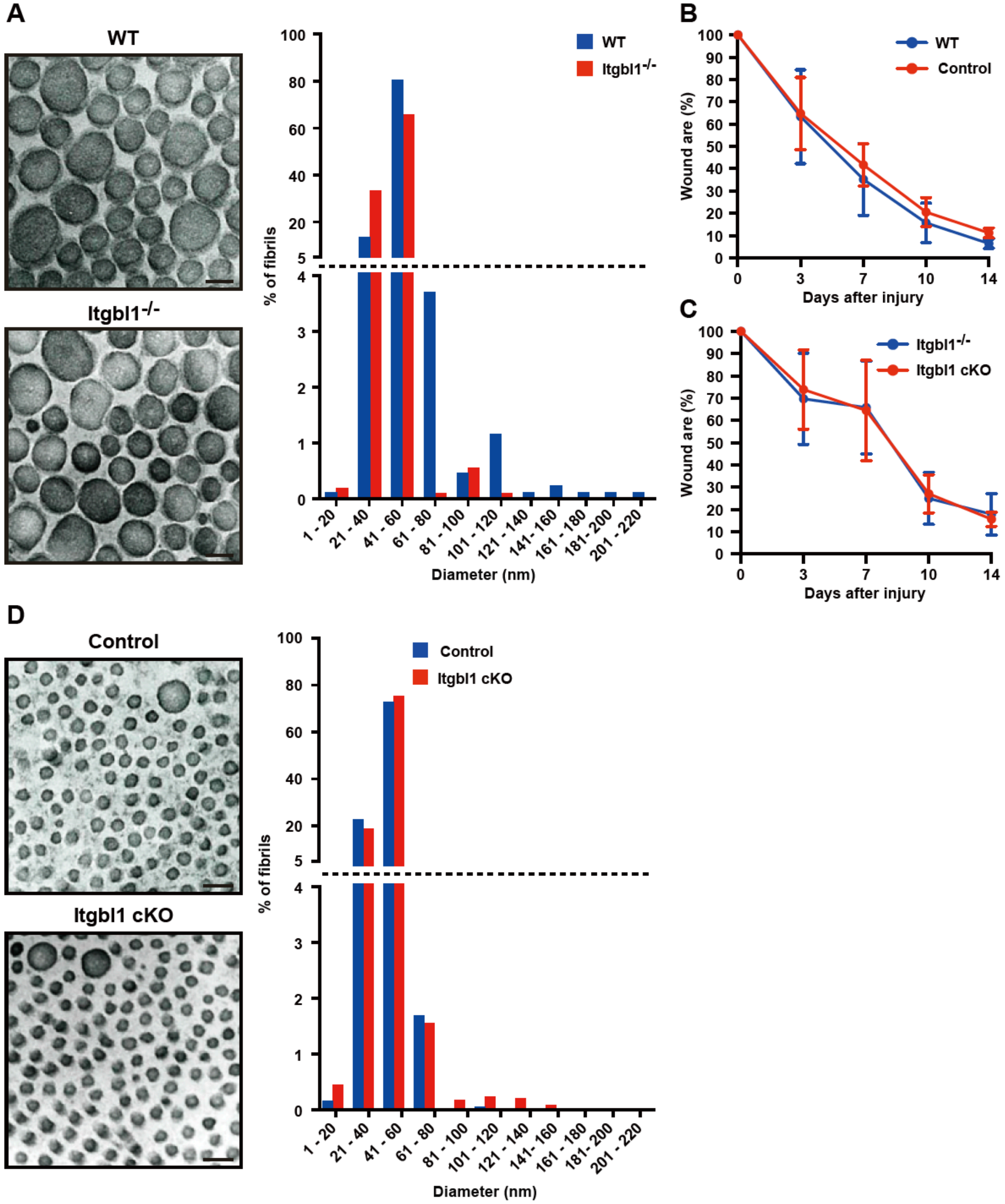

A
WT
Itgbl1-/-
100
80
60
40
20
5
4
3
2
1
0
% of fibrils
WT
Itgbl1-/-
1 - 20
21 - 40
41 - 60
61 - 80
81 - 100
101 - 120
121 - 140
141- 160
161 - 180
181- 200
201 - 220
Diameter (nm)
B
Wound are (%)
WT
Control
Days after injury
C
Wound are (%)
Itgbl1-/-
Itgbl1 cKO
Days after injury
D
Control
Itgbl1 cKO
% of fibrils
Control
Itgbl1 cKO
Diameter (nm)

Appendix Figure S6. Collagen fibril morphology and skin wound healing in WT and *Itgbl1* transgenic mice.

A. Representative TEM images of collagen fibrils in intact skin (left) and histogram of total range of fibril diameters (right); *n* = 150 fibrils from four WT mice; *n* = 182 fibrils from three *Itgbl1*$^{-/-}$ mice. Scale bars: 100 nm

B. Proportion of the wound area remaining open at each time point relative to the initial wound area in WT (*n* = 11) and control (*Itgbl1*$^{flox/flox}$; (*n* = 8) mice.

C. Proportion of the wound area remaining open at each time point relative to the initial wound area in *Itgbl1*$^{-/-}$ (*n* = 12) and *Itgbl1* conditional knockout (cKO, *Itgbl1*$^{flox/flox}$*::Tagln*$^{Cre/Cre}$; *n* = 16) mice.

D. Representative TEM images of collagen fibrils in connective tissue from mid-wound sites at Day 14 post injury (left). Histogram of total range of fibril diameters in the wound site at Day 14 post injury (right); *n* = 1,897 fibrils from six control mice; *n* = 3,289 fibrils from eight *Itgbl1*$^{-/-}$ mice. Scale bars: 100 nm

Data information: All values represent the mean ± SD (B and C).

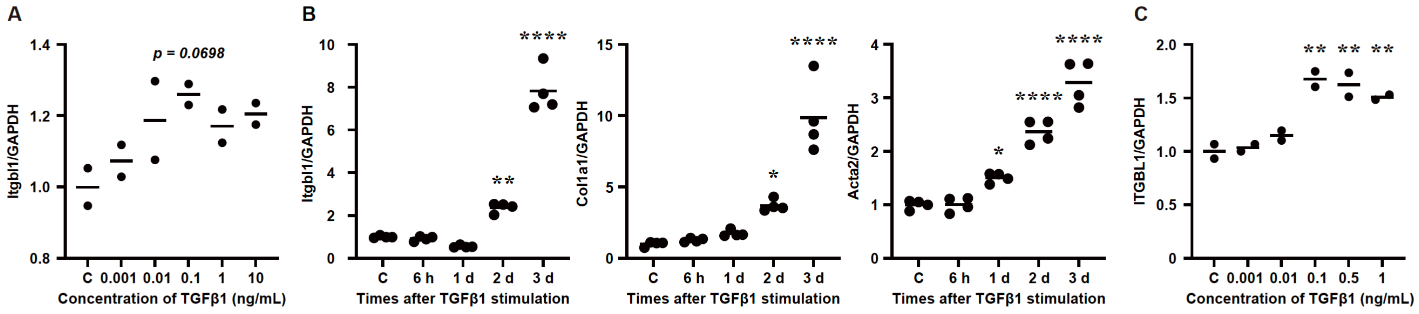

Appendix Figure S7. Regulation of *Itgbl1* expression in MDF and HDF treated with TGFβ1.

A. qPCR of *Itgbl1* expression relative to that of *GAPDH* in MDF stimulated with TGFβ1 for 1 day ($n = 2$).

B. qPCR measurement of temporal expression patterns of *Itgbl1*, *Col1a1*, and *Acta2* in MDF stimulated with TGFβ1 (100 pg/mL) ($n = 4$).

C. qPCR of *ITGBL1* expression relative to that of *GAPDH* in HDF stimulated with TGFβ1 for 1 day ($n = 2$).

Data information: All values represent the mean. One-way ANOVA followed by Dunnett's multiple comparisons test (control vs sample) (A–C) was used to generate the indicated *P* values; $*P < 0.05$, $**P < 0.01$, $****P < 0.0001$.

Appendix Table S1. References for marker genes in macrophage subclusters

M1	M2	M3	M5	M6
Cd74 (Leng *et al*, 2003)	*Mrc1* (Menzies *et al*, 2010)	*Arg1* (Gordon, 2003)	*Stmn1* *(Liu et al, 2022)*	*S100A8/A9* (Wang *et al*, 2018),
H2-EB1 (Stables *et al*, 2011)	*F13a1* (Martinez *et al*, 2006)	*Spp1* (Mori *et al*, 2008)	*Hmgb2* (Yano *et al*, 2022)	*G0s2* (Okabe *et al*, 2022)
H2-AB1 (Stables *et al.*, 2011)	*Wfdc17* (Karlstetter *et al*, 2010)	*Ctsd* (Krasniewski *et al*, 2022)	*Mki67* *(Gerdes et al, 1991)*	*Slpi* (Odaka *et al*, 2003)
Ccr2 (Sierra-Filardi *et al*, 2014)	*Cbr2* (Cochain *et al*, 2018) (Colin *et al*, 2014)	*Cd36* *(Yang et al, 2022)*	*Top2a* (Liu *et al.*, 2022)	*Acdo1* (Wu *et al*, 2020)
Il1b (Jablonski *et al*, 2015)	*Gas6* (Chen *et al*, 2019)	*Lgals3* (Bai *et al*, 2022)	*Tuba1b* (Hu *et al*, 2022)	*Hdc* (Xu *et al*, 2017),
		Fth1 *(Mesquita et al, 2020)*	*Hmgb1* (Salo *et al*, 2021)	*Il1r2* (Ehrchen *et al*, 2007)

Appendix Table S2. References for marker genes in fibroblast subclusters

F1	F2	F3	F4	F6	F7	F8
Mgp<br>(Canfield *et al*, 2002)	*Postn*<br>(Nunomura *et al*, 2018)	*Pi16*<br>(Buechler *et al*, 2021)	*Mfap4*<br>(Kanaan *et al*, 2022)	*Acta2*<br>*(McAndrews et al, 2022)*	*Cd74*<br>(Su *et al*, 2017)	*Mki67*<br>*(Gerdes et al., 1991)*
Eln<br>(Almine *et al*, 2012)	*Col5a3*<br>(DeNigris *et al*, 2016)	*C3*<br>(Friščić *et al*, 2021)	*Pcolce2*<br>(Baicu *et al*, 2012)	*Tagln*<br>(Lawson *et al*, 1997)	*Lyz2*<br>(Clausen *et al*, 1999)	*Pclaf*<br>(Tabib *et al*, 2021)
Cilp<br>(Groß & Thum, 2020)	*Lgals1*<br>(Lin *et al*, 2015)	*Prss23*<br>(Tabib *et al.*, 2021)	*Ecm2*<br>(Cheng *et al*, 2023)	*Col12a1*<br>(Muhl *et al*, 2020)	*Fcer1g*<br>(Dong *et al*, 2022)	*Stmn1*<br>(Hu *et al*, 2021)
Igf1<br>(Yin *et al*, 2020)	*Vcan*<br>(Hattori *et al*, 2011)	*Cd55*<br>(Karpus *et al*, 2015)	*Col15a1*<br>(Buechler *et al.*, 2021)	*Lrrc15*<br>(Krishnamurty *et al*, 2022)	*Apoe*<br>(Feng *et al*, 2022)	*Cks2*<br>(Miao *et al*, 2022)
Cygb<br>(Nakatani *et al*, 2004)	*Plod2*<br>(Knipper *et al*, 2015)	*Sema3c*<br>*(De Angelis Rigotti et al, 2023)*	*Ctsk*<br>(Quintanilla-Dieck *et al*, 2009)	*Tpm1*<br>(Huang *et al*, 2020)	*C1qc*<br>(Feng *et al.*, 2022)	*Tubb5*<br>(Mouton *et al*, 2019)
Gas6<br>(Bellan *et al*, 2019)	*Loxl2*<br>(Matsuo *et al*, 2021)	*Anxa3*<br>(Wang *et al*, 2019)	*Ppp1r14a*<br>(Ascensión *et al*, 2021)	*Cdh11*<br>(Chang *et al*, 2011)	*Tyrobp*<br>(Feng *et al.*, 2022)	*Hmgb2*<br>(Yano *et al.*, 2022)
Smoc2<br>(Gerarduzzi *et al*, 2017)		*Dpp4*<br>(Soare *et al*, 2020)		*Thbs2*<br>(Bornstein *et al*, 2000)	*C1qa*<br>(Feng *et al.*, 2022)	*Hmgb1*<br>(Wang *et al*, 2017)
Mmp3<br>(Kümper *et al*, 2022)		*Scara5*<br>(Ojala *et al*, 2013)		*Mmp14*<br>(Pach *et al*, 2021)		*H2afz*<br>(Yao *et al*, 2018)
						Birc5<br>*(Lin et al, 2020)*
						Nucks1<br>*(Huang et al, 2018)*

References

Almine JF, Wise SG, Weiss AS (2012) Elastin signaling in wound repair. *Birth Defects Res C Embryo Today* 96: 248-257

Ascensión AM, Fuertes-Álvarez S, Ibañez-Solé O, Izeta A, Araúzo-Bravo MJ (2021) Human Dermal Fibroblast Subpopulations Are Conserved across Single-Cell RNA Sequencing Studies. *J Invest Dermatol* 141: 1735-1744.e1735

Bai L, Lu W, Tang S, Tang H, Xu M, Liang C, Zheng S, Liu S, Kong M, Duan Z *et al* (2022) Galectin-3 critically mediates the hepatoprotection conferred by M2-like macrophages in ACLF by inhibiting pyroptosis but not necroptosis signalling. *Cell Death Dis* 13: 775

Baicu CF, Zhang Y, Van Laer AO, Renaud L, Zile MR, Bradshaw AD (2012) Effects of the absence of procollagen C-endopeptidase enhancer-2 on myocardial collagen accumulation in chronic pressure overload. *Am J Physiol Heart Circ Physiol* 303: H234-240

Bellan M, Cittone MG, Tonello S, Rigamonti C, Castello LM, Gavelli F, Pirisi M, Sainaghi PP (2019) Gas6/TAM System: A Key Modulator of the Interplay between Inflammation and Fibrosis. *Int J Mol Sci* 20

Bornstein P, Kyriakides TR, Yang Z, Armstrong LC, Birk DE (2000) Thrombospondin 2 modulates collagen fibrillogenesis and angiogenesis. *J Investig Dermatol Symp Proc* 5: 61-66

Buechler MB, Pradhan RN, Krishnamurty AT, Cox C, Calviello AK, Wang AW, Yang YA, Tam L, Caothien R, Roose-Girma M *et al* (2021) Cross-tissue organization of the fibroblast lineage. *Nature* 593: 575-579

Canfield AE, Farrington C, Dziobon MD, Boot-Handford RP, Heagerty AM, Kumar SN, Roberts IS (2002) The involvement of matrix glycoproteins in vascular calcification and fibrosis: an immunohistochemical study. *J Pathol* 196: 228-234

Chang SK, Noss EH, Chen M, Gu Z, Townsend K, Grenha R, Leon L, Lee SY, Lee DM, Brenner MB (2011) Cadherin-11 regulates fibroblast inflammation. *Proc Natl Acad Sci U S A* 108: 8402-8407

Chen SY, Chiang CF, Chiu KC, Cheng CW, Huang SM, Chen PH, Chen CY, Shieh YS (2019) Macrophage phenotypes and Gas6/Axl signaling in apical lesions. *J Dent Sci* 14: 281-287

Cheng X, Liu Z, Liang W, Zhu Q, Wang C, Wang H, Zhang J, Li P, Gao Y (2023) ECM2, a prognostic biomarker for lower grade glioma, serves as a potential novel target for immunotherapy. *Int J Biochem Cell Biol* 158: 106409

Clausen BE, Burkhardt C, Reith W, Renkawitz R, Förster I (1999) Conditional gene

targeting in macrophages and granulocytes using LysMcre mice. *Transgenic Res* 8: 265-277

Cochain C, Vafadarnejad E, Arampatzi P, Pelisek J, Winkels H, Ley K, Wolf D, Saliba AE, Zernecke A (2018) Single-Cell RNA-Seq Reveals the Transcriptional Landscape and Heterogeneity of Aortic Macrophages in Murine Atherosclerosis. *Circ Res* 122: 1661-1674

Colin S, Chinetti-Gbaguidi G, Staels B (2014) Macrophage phenotypes in atherosclerosis. *Immunol Rev* 262: 153-166

De Angelis Rigotti F, Wiedmann L, Hubert MO, Vacca M, Hasan SS, Moll I, Carvajal S, Jiménez W, Starostecka M, Billeter AT *et al* (2023) Semaphorin 3C exacerbates liver fibrosis. *Hepatology*

DeNigris J, Yao Q, Birk EK, Birk DE (2016) Altered dermal fibroblast behavior in a collagen V haploinsufficient murine model of classic Ehlers-Danlos syndrome. *Connect Tissue Res* 57: 1-9

Dong K, Chen W, Pan X, Wang H, Sun Y, Qian C, Chen W, Wang C, Yang F, Cui X (2022) FCER1G positively relates to macrophage infiltration in clear cell renal cell carcinoma and contributes to unfavorable prognosis by regulating tumor immunity. *BMC Cancer* 22: 140

Ehrchen J, Steinmüller L, Barczyk K, Tenbrock K, Nacken W, Eisenacher M, Nordhues U, Sorg C, Sunderkötter C, Roth J (2007) Glucocorticoids induce differentiation of a specifically activated, anti-inflammatory subtype of human monocytes. *Blood* 109: 1265-1274

Feng C, Shan M, Xia Y, Zheng Z, He K, Wei Y, Song K, Meng T, Liu H, Hao Y *et al* (2022) Single-cell RNA sequencing reveals distinct immunology profiles in human keloid. *Front Immunol* 13: 940645

Friščić J, Böttcher M, Reinwald C, Bruns H, Wirth B, Popp SJ, Walker KI, Ackermann JA, Chen X, Turner J *et al* (2021) The complement system drives local inflammatory tissue priming by metabolic reprogramming of synovial fibroblasts. *Immunity* 54: 1002-1021.e1010

Gerarduzzi C, Kumar RK, Trivedi P, Ajay AK, Iyer A, Boswell S, Hutchinson JN, Waikar SS, Vaidya VS (2017) Silencing SMOC2 ameliorates kidney fibrosis by inhibiting fibroblast to myofibroblast transformation. *JCI Insight* 2

Gerdes J, Li L, Schlueter C, Duchrow M, Wohlenberg C, Gerlach C, Stahmer I, Kloth S, Brandt E, Flad HD (1991) Immunobiochemical and molecular biologic characterization of the cell proliferation-associated nuclear antigen that is defined by monoclonal antibody Ki-67. *Am J Pathol* 138: 867-873

Gordon S (2003) Alternative activation of macrophages. *Nat Rev Immunol* 3: 23-35
Groß S, Thum T (2020) TGF-β Inhibitor CILP as a Novel Biomarker for Cardiac Fibrosis. *JACC Basic Transl Sci* 5: 444-446
Hattori N, Carrino DA, Lauer ME, Vasanji A, Wylie JD, Nelson CM, Apte SS (2011) Pericellular versican regulates the fibroblast-myofibroblast transition: a role for ADAMTS5 protease-mediated proteolysis. *J Biol Chem* 286: 34298-34310
Hu C, Chu C, Liu L, Wang C, Jin S, Yang R, Rung S, Li J, Qu Y, Man Y (2021) Dissecting the microenvironment around biosynthetic scaffolds in murine skin wound healing. *Sci Adv* 7
Hu X, Zhu H, Chen B, He X, Shen Y, Zhang X, Chen W, Liu X, Xu Y, Xu X (2022) Tubulin Alpha 1b Is Associated with the Immune Cell Infiltration and the Response of HCC Patients to Immunotherapy. *Diagnostics (Basel)* 12
Huang H, Huang X, Luo S, Zhang H, Hu F, Chen R, Huang C, Su Z (2020) The MicroRNA MiR-29c Alleviates Renal Fibrosis via TPM1-Mediated Suppression of the Wnt/β-Catenin Pathway. *Front Physiol* 11: 331
Huang P, Cai Y, Zhao B, Cui L (2018) Roles of NUCKS1 in Diseases: Susceptibility, Potential Biomarker, and Regulatory Mechanisms. *Biomed Res Int* 2018: 7969068
Jablonski KA, Amici SA, Webb LM, Ruiz-Rosado Jde D, Popovich PG, Partida-Sanchez S, Guerau-de-Arellano M (2015) Novel Markers to Delineate Murine M1 and M2 Macrophages. *PLoS One* 10: e0145342
Kanaan R, Medlej-Hashim M, Jounblat R, Pilecki B, Sorensen GL (2022) Microfibrillar-associated protein 4 in health and disease. *Matrix Biol* 111: 1-25
Karlstetter M, Walczak Y, Weigelt K, Ebert S, Van den Brulle J, Schwer H, Fuchshofer R, Langmann T (2010) The novel activated microglia/macrophage WAP domain protein, AMWAP, acts as a counter-regulator of proinflammatory response. *J Immunol* 185: 3379-3390
Karpus ON, Kiener HP, Niederreiter B, Yilmaz-Elis AS, van der Kaa J, Ramaglia V, Arens R, Smolen JS, Botto M, Tak PP *et al* (2015) CD55 deposited on synovial collagen fibers protects from immune complex-mediated arthritis. *Arthritis Res Ther* 17: 6
Knipper JA, Willenborg S, Brinckmann J, Bloch W, Maaß T, Wagener R, Krieg T, Sutherland T, Munitz A, Rothenberg ME *et al* (2015) Interleukin-4 Receptor α Signaling in Myeloid Cells Controls Collagen Fibril Assembly in Skin Repair. *Immunity* 43: 803-816
Krasniewski LK, Chakraborty P, Cui CY, Mazan-Mamczarz K, Dunn C, Piao Y, Fan J, Shi C, Wallace T, Nguyen C *et al* (2022) Single-cell analysis of skeletal muscle macrophages reveals age-associated functional subpopulations. *Elife* 11

Krishnamurty AT, Shyer JA, Thai M, Gandham V, Buechler MB, Yang YA, Pradhan RN, Wang AW, Sanchez PL, Qu Y *et al* (2022) LRRC15(+) myofibroblasts dictate the stromal setpoint to suppress tumour immunity. *Nature* 611: 148-154

Kümper M, Zamek J, Steinkamp J, Pach E, Mauch C, Zigrino P (2022) Role of MMP3 and fibroblast-MMP14 in skin homeostasis and repair. *Eur J Cell Biol* 101: 151276

Lawson D, Harrison M, Shapland C (1997) Fibroblast transgelin and smooth muscle SM22alpha are the same protein, the expression of which is down-regulated in many cell lines. *Cell Motil Cytoskeleton* 38: 250-257

Leng L, Metz CN, Fang Y, Xu J, Donnelly S, Baugh J, Delohery T, Chen Y, Mitchell RA, Bucala R (2003) MIF signal transduction initiated by binding to CD74. *J Exp Med* 197: 1467-1476

Lin TY, Chan HH, Chen SH, Sarvagalla S, Chen PS, Coumar MS, Cheng SM, Chang YC, Lin CH, Leung E *et al* (2020) BIRC5/Survivin is a novel ATG12-ATG5 conjugate interactor and an autophagy-induced DNA damage suppressor in human cancer and mouse embryonic fibroblast cells. *Autophagy* 16: 1296-1313

Lin YT, Chen JS, Wu MH, Hsieh IS, Liang CH, Hsu CL, Hong TM, Chen YL (2015) Galectin-1 accelerates wound healing by regulating the neuropilin-1/Smad3/NOX4 pathway and ROS production in myofibroblasts. *J Invest Dermatol* 135: 258-268

Liu R, Han C, Hu J, Zhang B, Luo W, Ling F (2022) Infiltration of Apoptotic M2 Macrophage Subpopulation Is Negatively Correlated with the Immunotherapy Response in Colorectal Cancer. *Int J Mol Sci* 23

Martinez FO, Gordon S, Locati M, Mantovani A (2006) Transcriptional profiling of the human monocyte-to-macrophage differentiation and polarization: new molecules and patterns of gene expression. *J Immunol* 177: 7303-7311

Matsuo A, Tanida R, Yanagi S, Tsubouchi H, Miura A, Shigekusa T, Matsumoto N, Nakazato M (2021) Significance of nuclear LOXL2 inhibition in fibroblasts and myofibroblasts in the fibrotic process of acute respiratory distress syndrome. *Eur J Pharmacol* 892: 173754

McAndrews KM, Miyake T, Ehsanipour EA, Kelly PJ, Becker LM, McGrail DJ, Sugimoto H, LeBleu VS, Ge Y, Kalluri R (2022) Dermal αSMA(+) myofibroblasts orchestrate skin wound repair via β1 integrin and independent of type I collagen production. *Embo j* 41: e109470

Menzies FM, Henriquez FL, Alexander J, Roberts CW (2010) Sequential expression of macrophage anti-microbial/inflammatory and wound healing markers following innate, alternative and classical activation. *Clin Exp Immunol* 160: 369-379

Mesquita G, Silva T, Gomes AC, Oliveira PF, Alves MG, Fernandes R, Almeida AA,

Moreira AC, Gomes MS (2020) H-Ferritin is essential for macrophages' capacity to store or detoxify exogenously added iron. *Sci Rep* 10: 3061

Miao Y, Su D, Fu Q, Chen T, Ji Y, Zhang F (2022) Identification of CKS2 and RRM2 as potential markers of vitiligo using bioinformatics analysis. *Medicine (Baltimore)* 101: e31908

Mori R, Shaw TJ, Martin P (2008) Molecular mechanisms linking wound inflammation and fibrosis: knockdown of osteopontin leads to rapid repair and reduced scarring. *J Exp Med* 205: 43-51

Mouton AJ, Ma Y, Rivera Gonzalez OJ, Daseke MJ, 2nd, Flynn ER, Freeman TC, Garrett MR, DeLeon-Pennell KY, Lindsey ML (2019) Fibroblast polarization over the myocardial infarction time continuum shifts roles from inflammation to angiogenesis. *Basic Res Cardiol* 114: 6

Muhl L, Genové G, Leptidis S, Liu J, He L, Mocci G, Sun Y, Gustafsson S, Buyandelger B, Chivukula IV *et al* (2020) Single-cell analysis uncovers fibroblast heterogeneity and criteria for fibroblast and mural cell identification and discrimination. *Nat Commun* 11: 3953

Nakatani K, Okuyama H, Shimahara Y, Saeki S, Kim DH, Nakajima Y, Seki S, Kawada N, Yoshizato K (2004) Cytoglobin/STAP, its unique localization in splanchnic fibroblast-like cells and function in organ fibrogenesis. *Lab Invest* 84: 91-101

Nunomura S, Nanri Y, Ogawa M, Arima K, Mitamura Y, Yoshihara T, Hasuwa H, Conway SJ, Izuhara K (2018) Constitutive overexpression of periostin delays wound healing in mouse skin. *Wound Repair Regen* 26: 6-15

Odaka C, Mizuochi T, Yang J, Ding A (2003) Murine macrophages produce secretory leukocyte protease inhibitor during clearance of apoptotic cells: implications for resolution of the inflammatory response. *J Immunol* 171: 1507-1514

Ojala JR, Pikkarainen T, Elmberger G, Tryggvason K (2013) Progressive reactive lymphoid connective tissue disease and development of autoantibodies in scavenger receptor A5-deficient mice. *Am J Pathol* 182: 1681-1695

Okabe M, Takarada S, Miyao N, Nakaoka H, Ibuki K, Ozawa S, Watanabe K, Tsuji H, Hashimoto I, Hatasaki K *et al* (2022) G0S2 regulates innate immunity in Kawasaki disease via lncRNA HSD11B1-AS1. *Pediatr Res* 92: 378-387

Pach E, Kümper M, Fromme JE, Zamek J, Metzen F, Koch M, Mauch C, Zigrino P (2021) Extracellular Matrix Remodeling by Fibroblast-MMP14 Regulates Melanoma Growth. *Int J Mol Sci* 22

Quintanilla-Dieck MJ, Codriansky K, Keady M, Bhawan J, Rünger TM (2009) Expression and regulation of cathepsin K in skin fibroblasts. *Exp Dermatol* 18: 596-602

Salo H, Qu H, Mitsiou D, Aucott H, Han J, Zhang XM, Aulin C, Erlandsson Harris H (2021) Disulfide and Fully Reduced HMGB1 Induce Different Macrophage Polarization and Migration Patterns. *Biomolecules* 11

Sierra-Filardi E, Nieto C, Domínguez-Soto A, Barroso R, Sánchez-Mateos P, Puig-Kroger A, López-Bravo M, Joven J, Ardavín C, Rodríguez-Fernández JL *et al* (2014) CCL2 shapes macrophage polarization by GM-CSF and M-CSF: identification of CCL2/CCR2-dependent gene expression profile. *J Immunol* 192: 3858-3867

Soare A, Györfi HA, Matei AE, Dees C, Rauber S, Wohlfahrt T, Chen CW, Ludolph I, Horch RE, Bäuerle T *et al* (2020) Dipeptidylpeptidase 4 as a Marker of Activated Fibroblasts and a Potential Target for the Treatment of Fibrosis in Systemic Sclerosis. *Arthritis Rheumatol* 72: 137-149

Stables MJ, Shah S, Camon EB, Lovering RC, Newson J, Bystrom J, Farrow S, Gilroy DW (2011) Transcriptomic analyses of murine resolution-phase macrophages. *Blood* 118: e192-208

Su H, Na N, Zhang X, Zhao Y (2017) The biological function and significance of CD74 in immune diseases. *Inflamm Res* 66: 209-216

Tabib T, Huang M, Morse N, Papazoglou A, Behera R, Jia M, Bulik M, Monier DE, Benos PV, Chen W *et al* (2021) Myofibroblast transcriptome indicates SFRP2(hi) fibroblast progenitors in systemic sclerosis skin. *Nat Commun* 12: 4384

Wang L, Li X, Ren Y, Geng H, Zhang Q, Cao L, Meng Z, Wu X, Xu M, Xu K (2019) Cancer-associated fibroblasts contribute to cisplatin resistance by modulating ANXA3 in lung cancer cells. *Cancer Sci* 110: 1609-1620

Wang Q, Wang J, Wang J, Hong S, Han F, Chen J, Chen G (2017) HMGB1 induces lung fibroblast to myofibroblast differentiation through NF-κB-mediated TGF-β1 release. *Mol Med Rep* 15: 3062-3068

Wang S, Song R, Wang Z, Jing Z, Wang S, Ma J (2018) S100A8/A9 in Inflammation. *Front Immunol* 9: 1298

Wu R, Chen F, Wang N, Tang D, Kang R (2020) ACOD1 in immunometabolism and disease. *Cell Mol Immunol* 17: 822-833

Xu L, Cheng D, Huang Z, Ding S, Zhang W, Tan H, Shi H, Chen R, Zou Y, Wang TC *et al* (2017) Histamine promotes the differentiation of macrophages from CD11b(+) myeloid cells and formation of foam cells through a Stat6-dependent pathway. *Atherosclerosis* 263: 42-52

Yang P, Qin H, Li Y, Xiao A, Zheng E, Zeng H, Su C, Luo X, Lu Q, Liao M *et al* (2022) CD36-mediated metabolic crosstalk between tumor cells and macrophages affects liver metastasis. *Nat Commun* 13: 5782

Yano K, Choijookhuu N, Ikenoue M, Fidya, Fukaya T, Sato K, Lee D, Taniguchi N, Chosa E, Nanashima A *et al* (2022) Spatiotemporal expression of HMGB2 regulates cell proliferation and hepatocyte size during liver regeneration. *Sci Rep* 12: 11962

Yao F, Yu P, Li Y, Yuan X, Li Z, Zhang T, Liu F, Wang Y, Wang Y, Li D *et al* (2018) Histone Variant H2A.Z Is Required for the Maintenance of Smooth Muscle Cell Identity as Revealed by Single-Cell Transcriptomics. *Circulation* 138: 2274-2288

Yin Y, Han Y, Shi C, Xia Z (2020) IGF-1 regulates the growth of fibroblasts and extracellular matrix deposition in pelvic organ prolapse. *Open Med (Wars)* 15: 833-840
