# Supplementary figures and images for: Novel integrated multiomics analysis reveals a key role for integrin beta-like 1 in wound scarring
Source: EMBO Rep. 2024 Nov 18;26(1):122–52. doi: 10.1038/s44319-024-00322-3 (PMC11724056; doi:10.1038/s44319-024-00322-3)

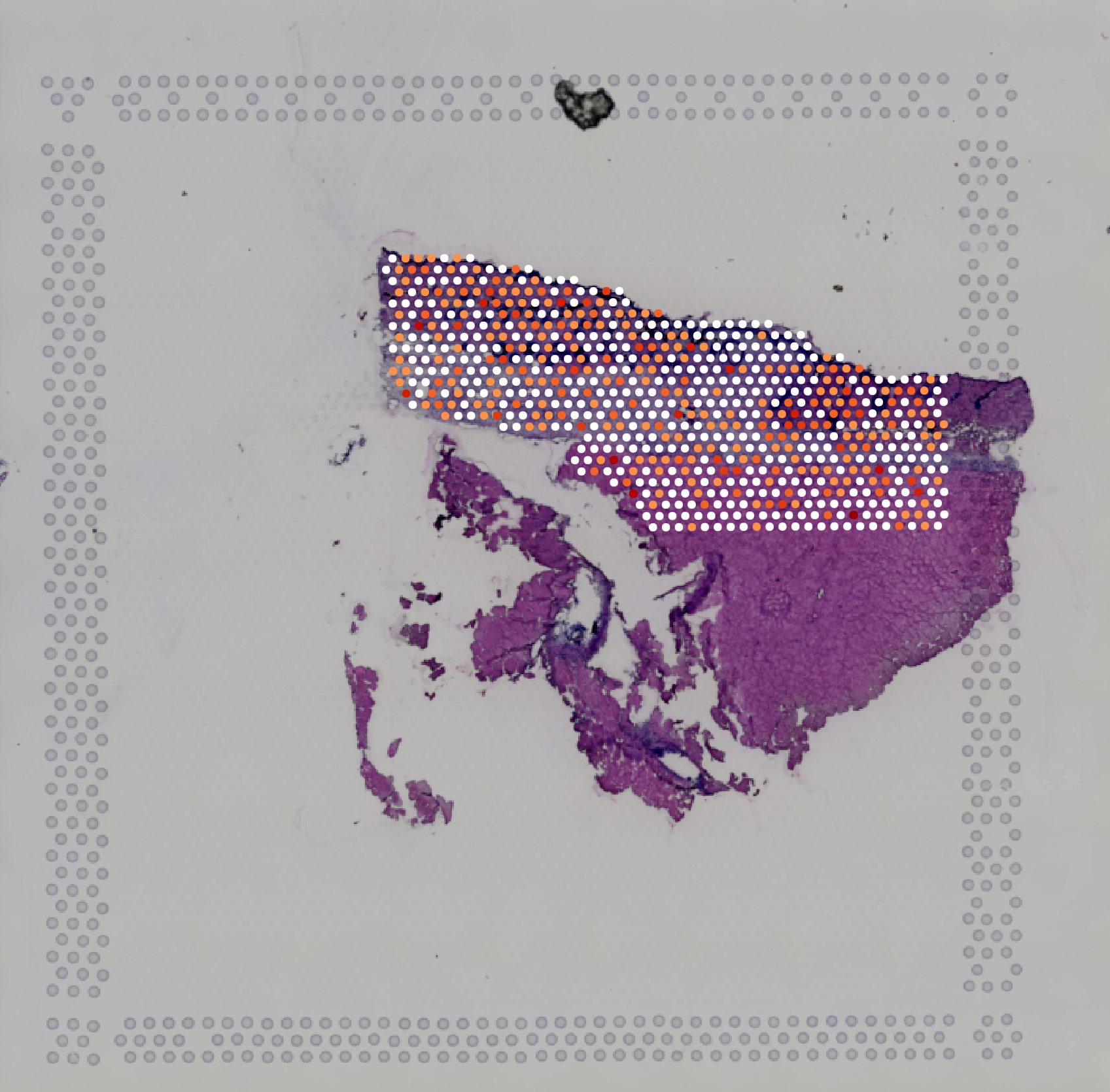

Supplement: Supplementary file 16 — Source data Fig. 3 [file 44319_2024_322_MOESM16_ESM.zip › SD figure 3/Figure3B/Day 14/C3.tif]

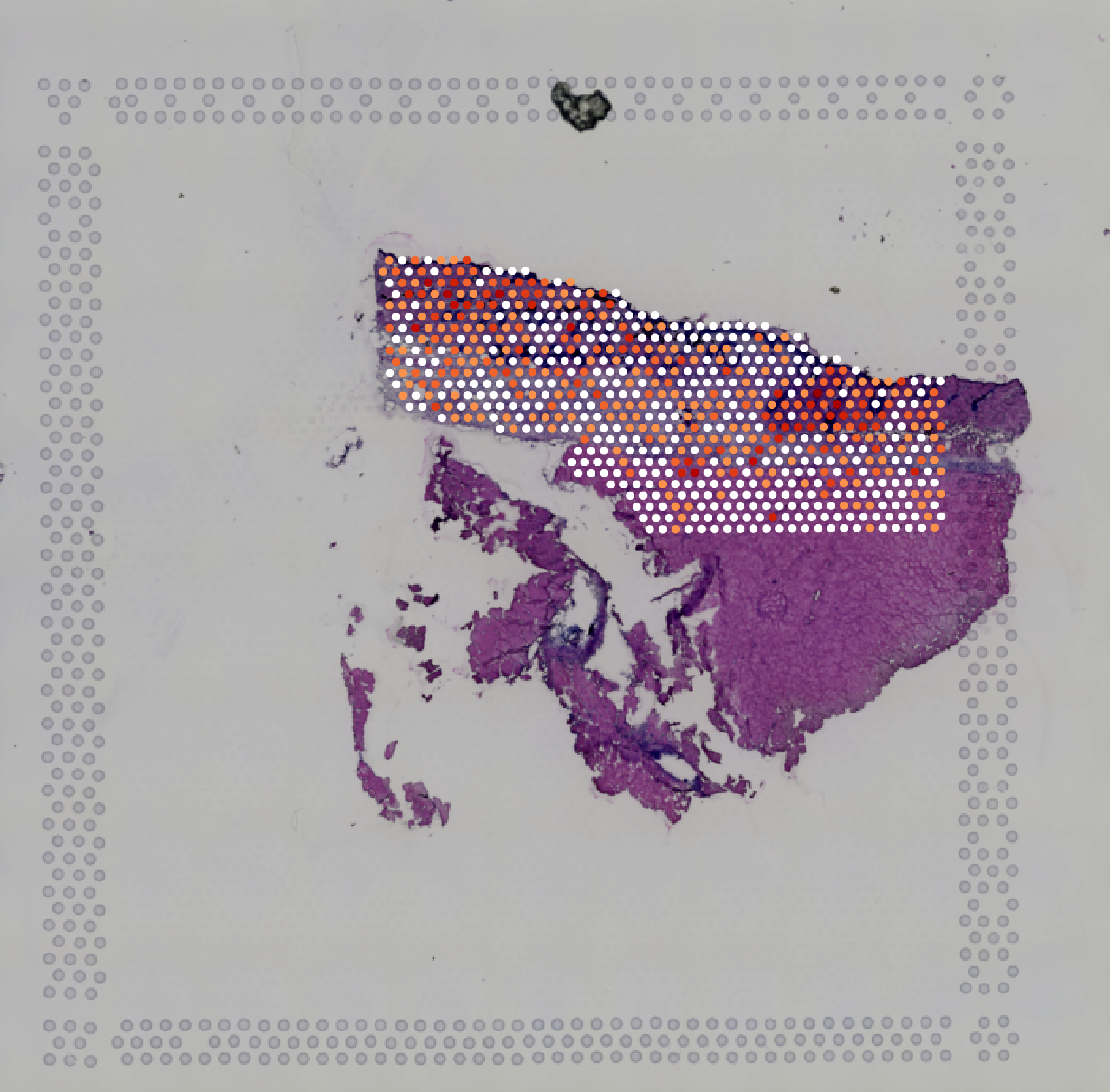

Supplement: Supplementary file 16 — Source data Fig. 3 [file 44319_2024_322_MOESM16_ESM.zip › SD figure 3/Figure3B/Day 14/C4b.tif]

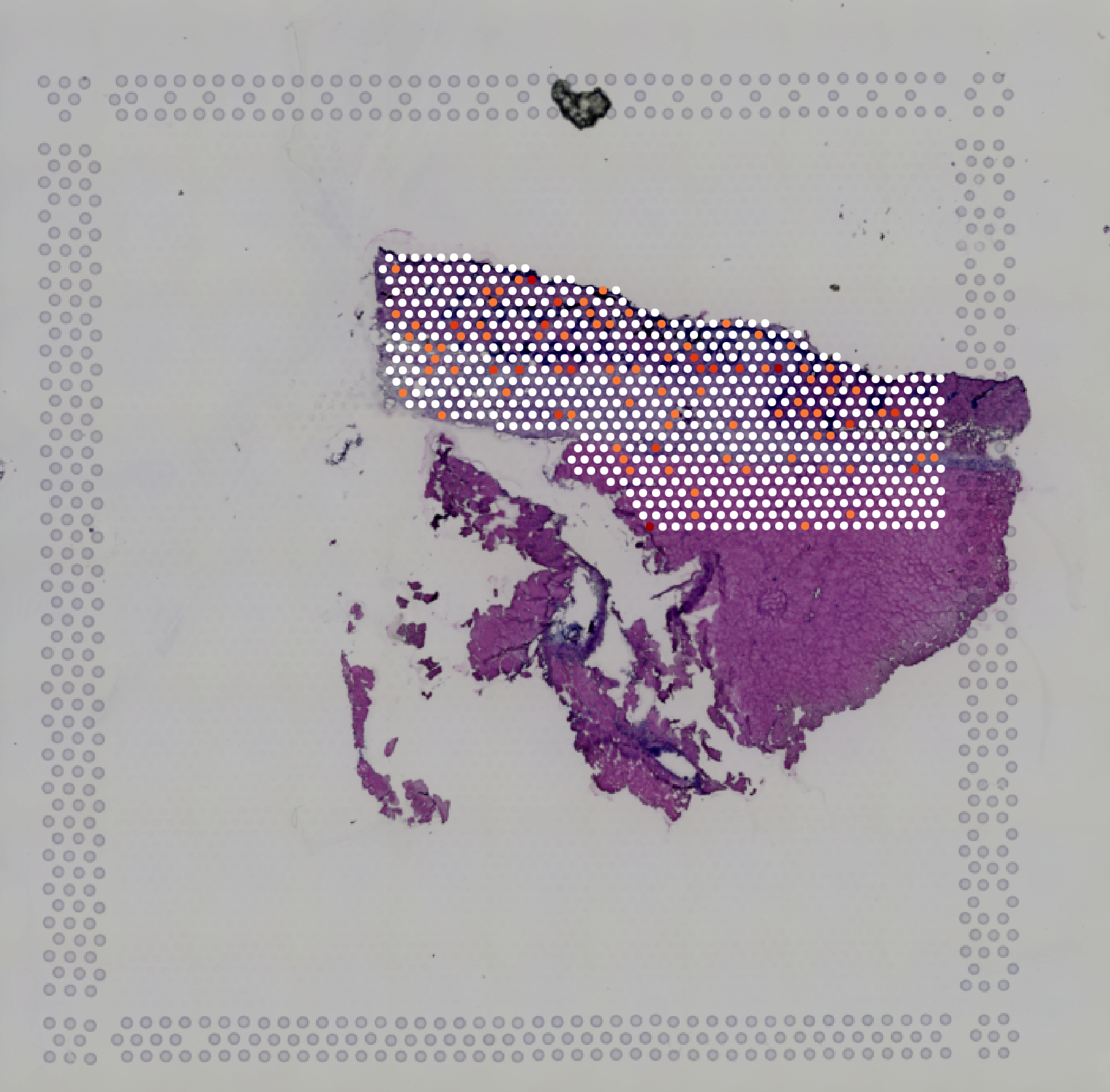

Supplement: Supplementary file 16 — Source data Fig. 3 [file 44319_2024_322_MOESM16_ESM.zip › SD figure 3/Figure3B/Day 14/Ccl11.tif]

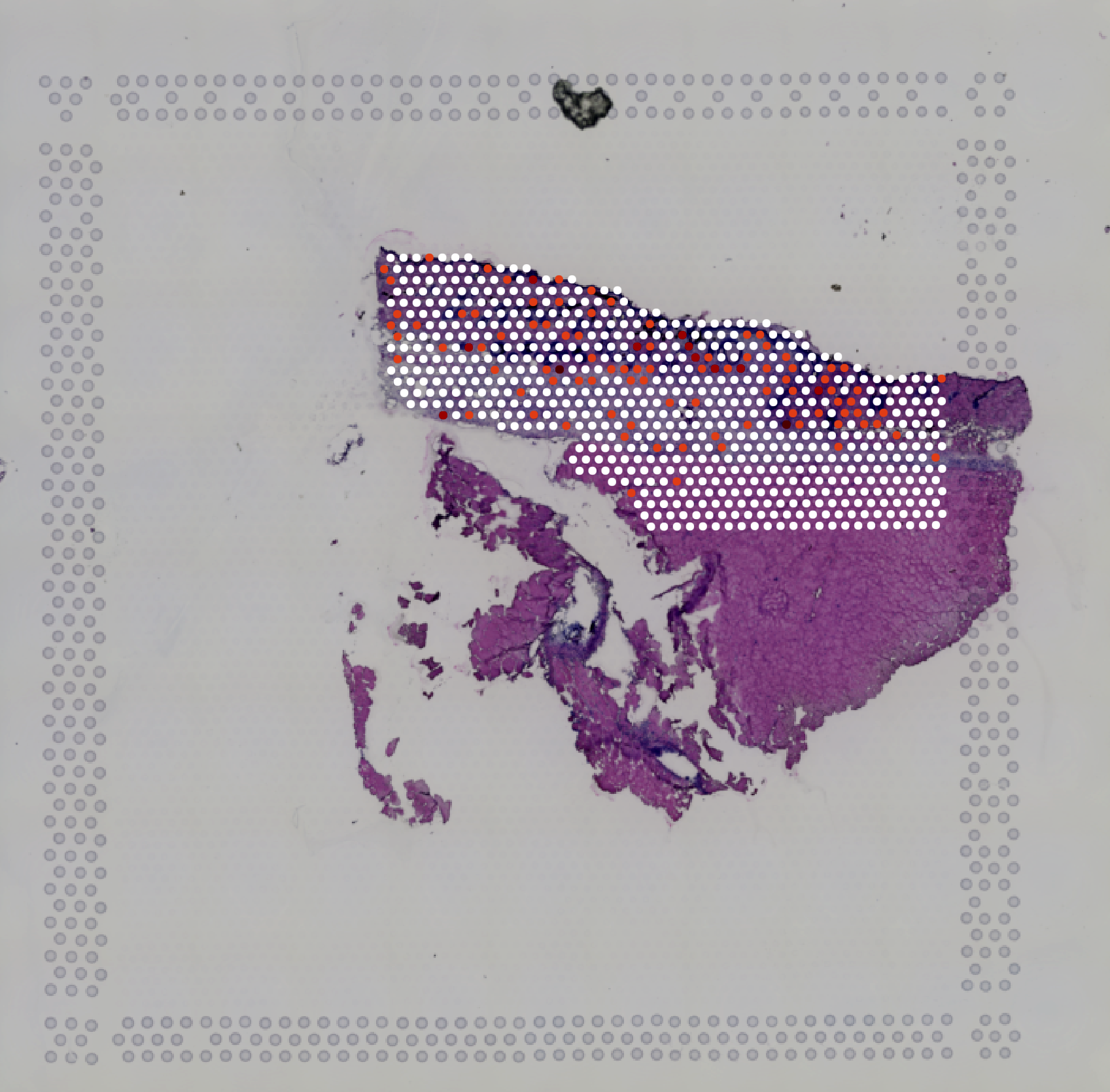

Supplement: Supplementary file 16 — Source data Fig. 3 [file 44319_2024_322_MOESM16_ESM.zip › SD figure 3/Figure3B/Day 14/Ccl7.tif]

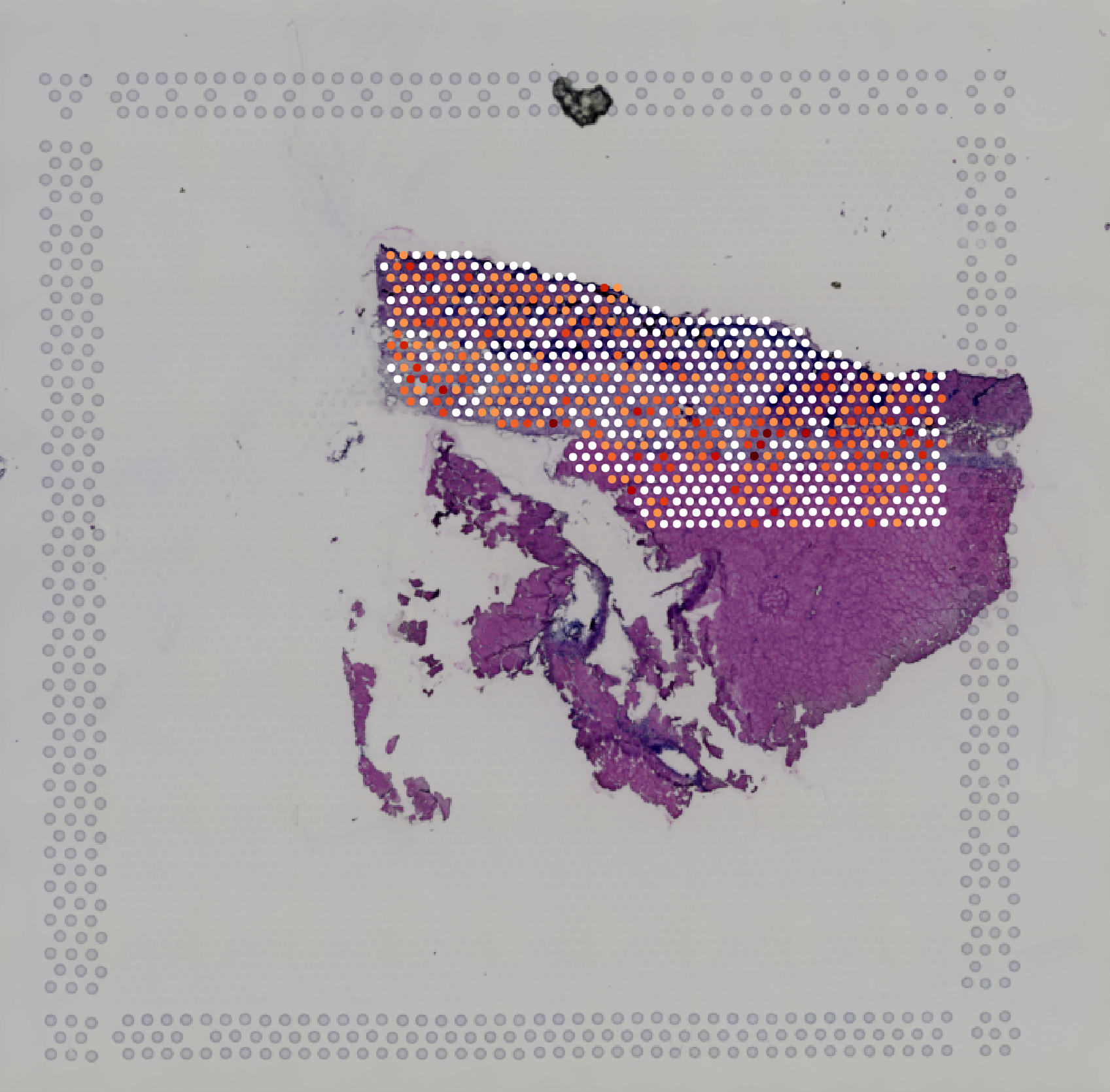

Supplement: Supplementary file 16 — Source data Fig. 3 [file 44319_2024_322_MOESM16_ESM.zip › SD figure 3/Figure3B/Day 14/Clec3b.tif]

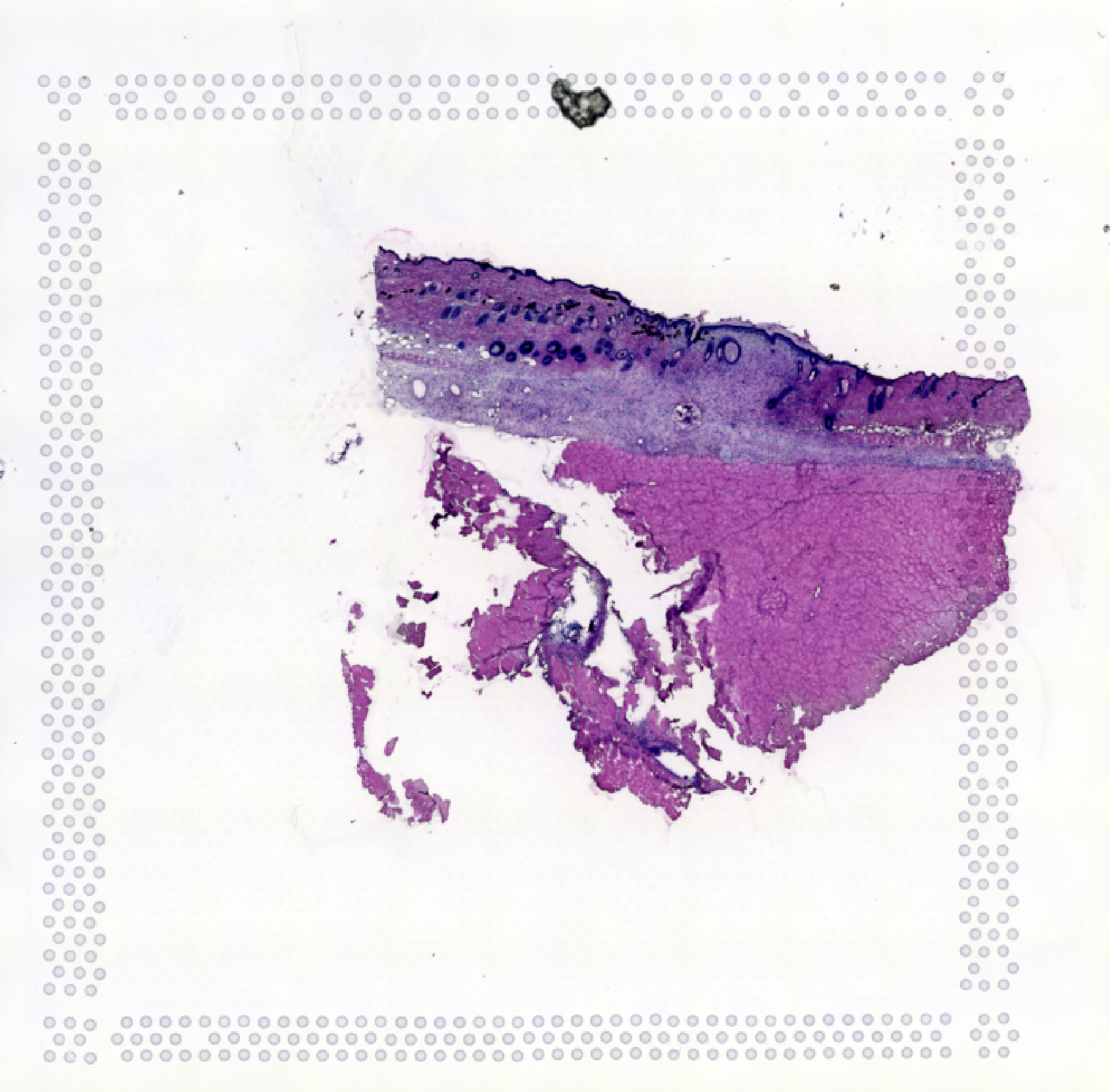

Supplement: Supplementary file 16 — Source data Fig. 3 [file 44319_2024_322_MOESM16_ESM.zip › SD figure 3/Figure3B/Day 14/HE.tif]

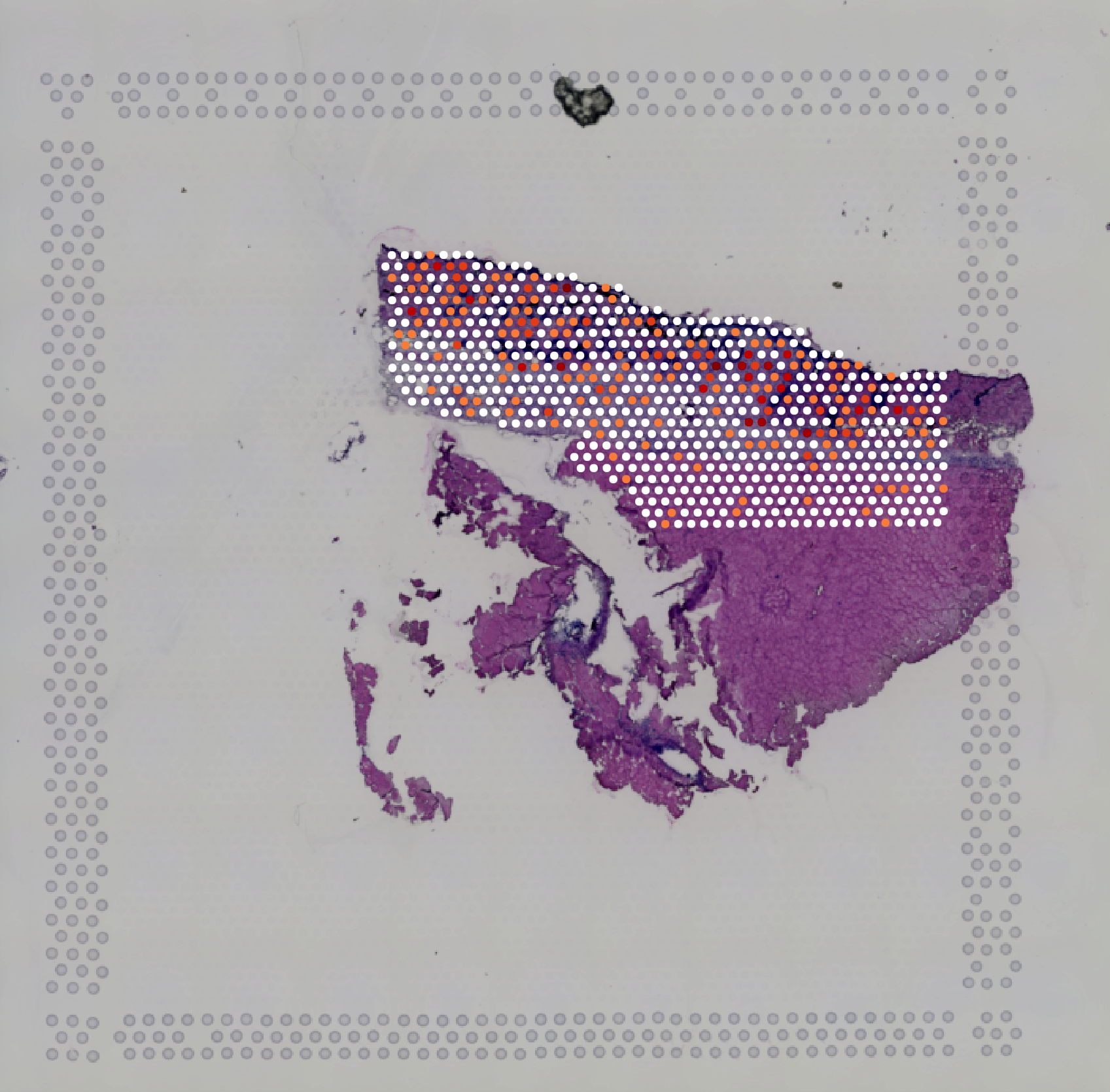

Supplement: Supplementary file 16 — Source data Fig. 3 [file 44319_2024_322_MOESM16_ESM.zip › SD figure 3/Figure3B/Day 14/Itgbl1.tif]

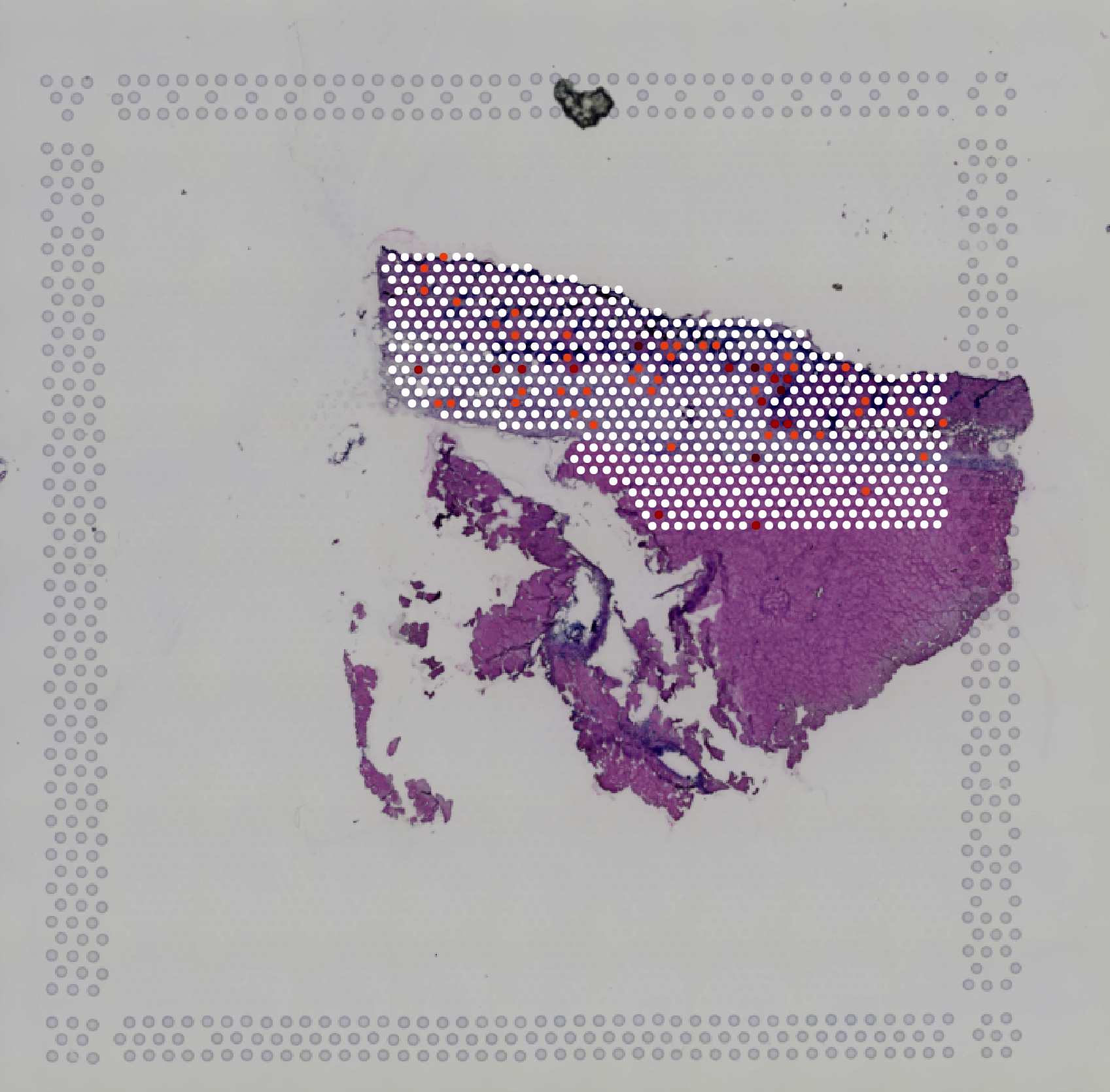

Supplement: Supplementary file 16 — Source data Fig. 3 [file 44319_2024_322_MOESM16_ESM.zip › SD figure 3/Figure3B/Day 14/Lrrc17.tif]

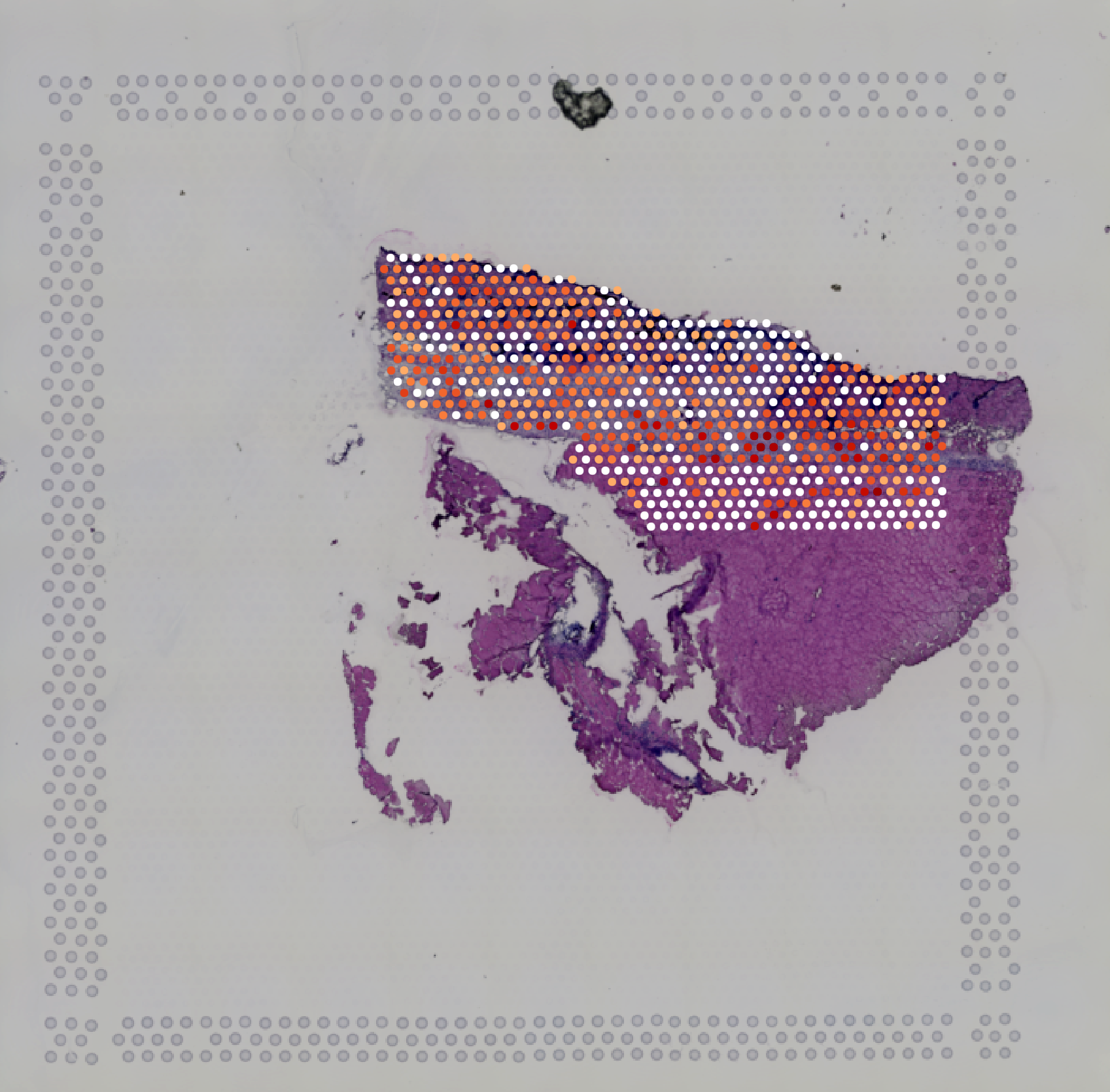

Supplement: Supplementary file 16 — Source data Fig. 3 [file 44319_2024_322_MOESM16_ESM.zip › SD figure 3/Figure3B/Day 14/Pi16.tif]

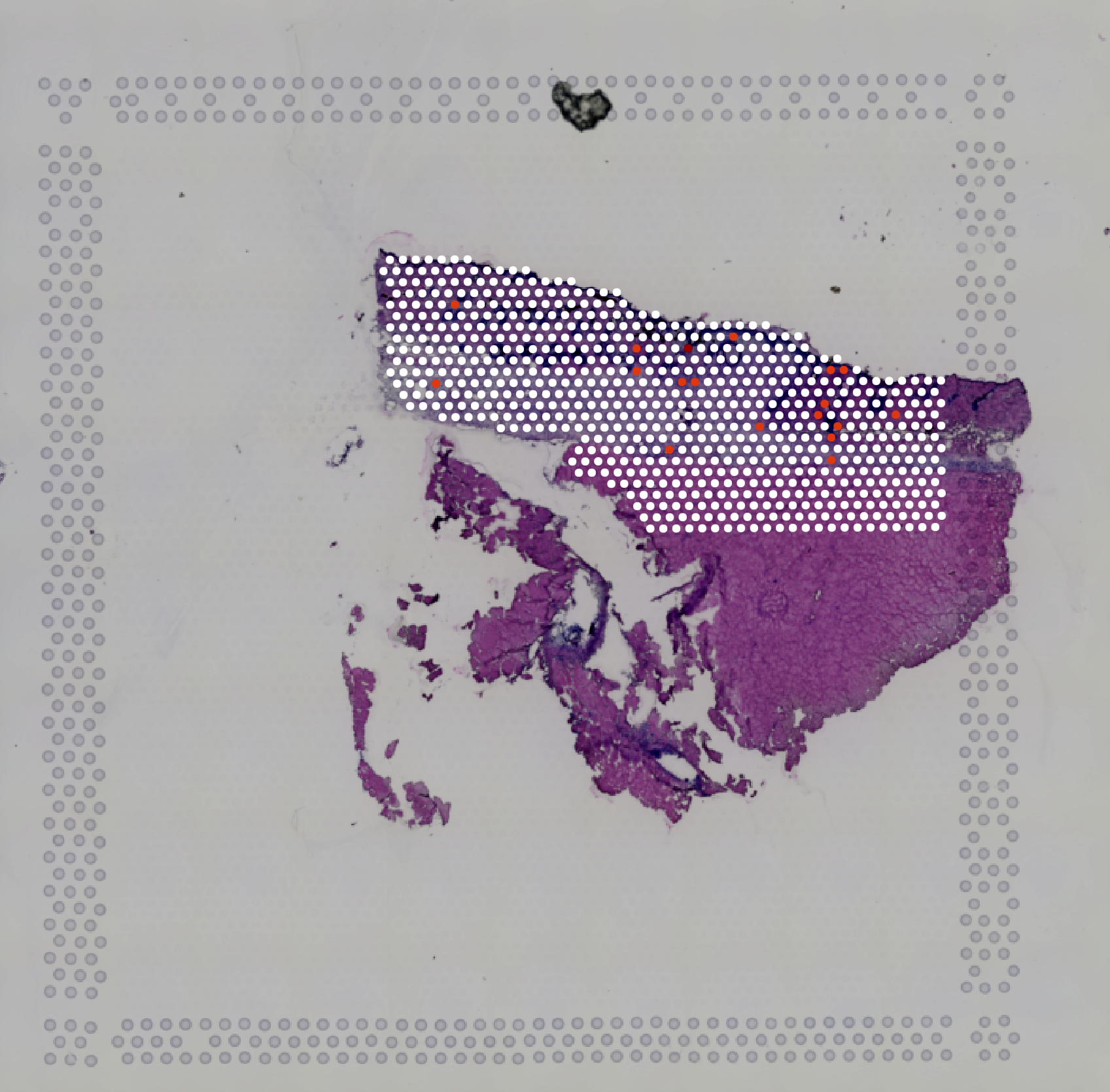

Supplement: Supplementary file 16 — Source data Fig. 3 [file 44319_2024_322_MOESM16_ESM.zip › SD figure 3/Figure3B/Day 14/Saa3.tif]

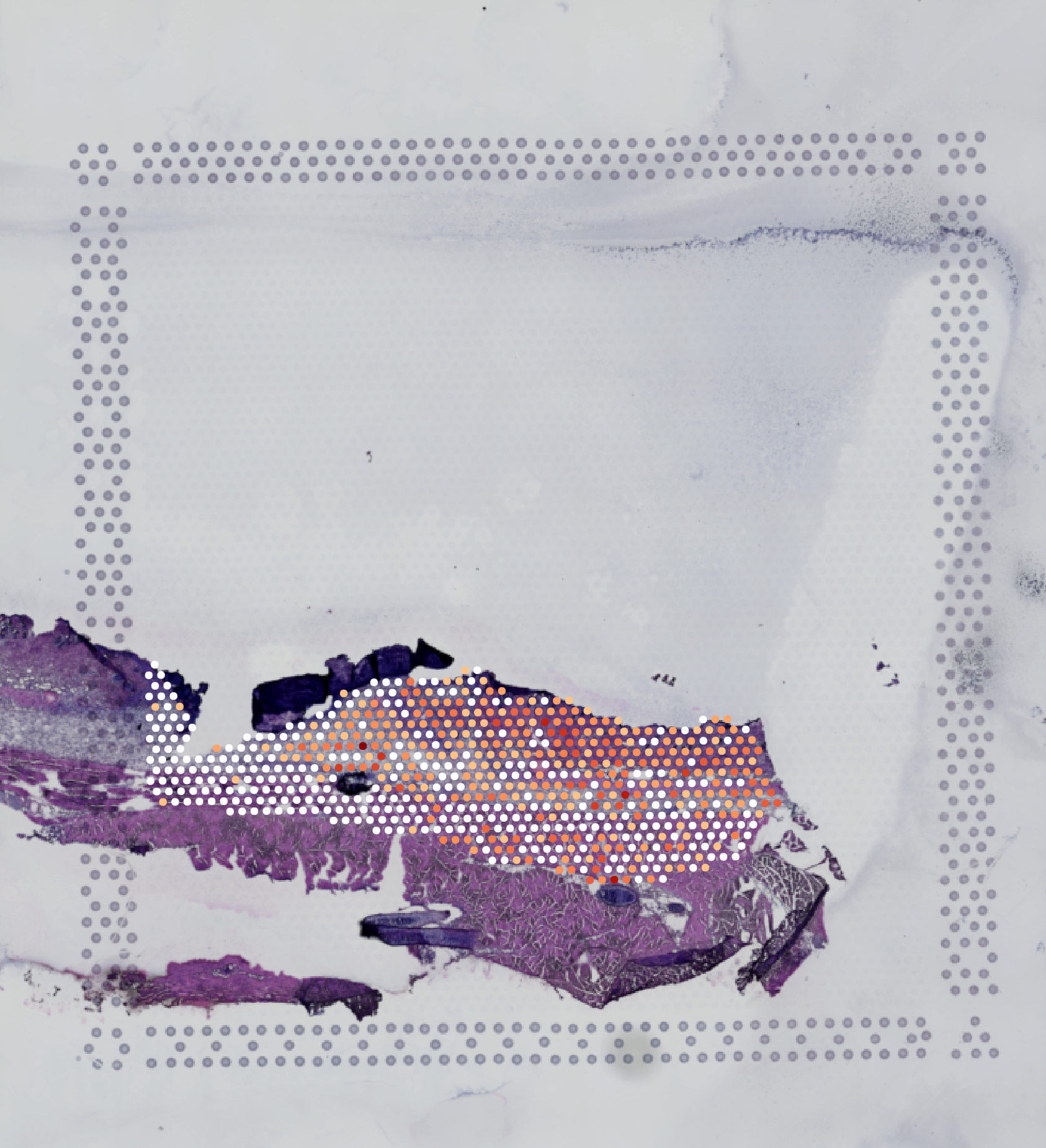

Supplement: Supplementary file 16 — Source data Fig. 3 [file 44319_2024_322_MOESM16_ESM.zip › SD figure 3/Figure3B/Day 3/C3.tif]

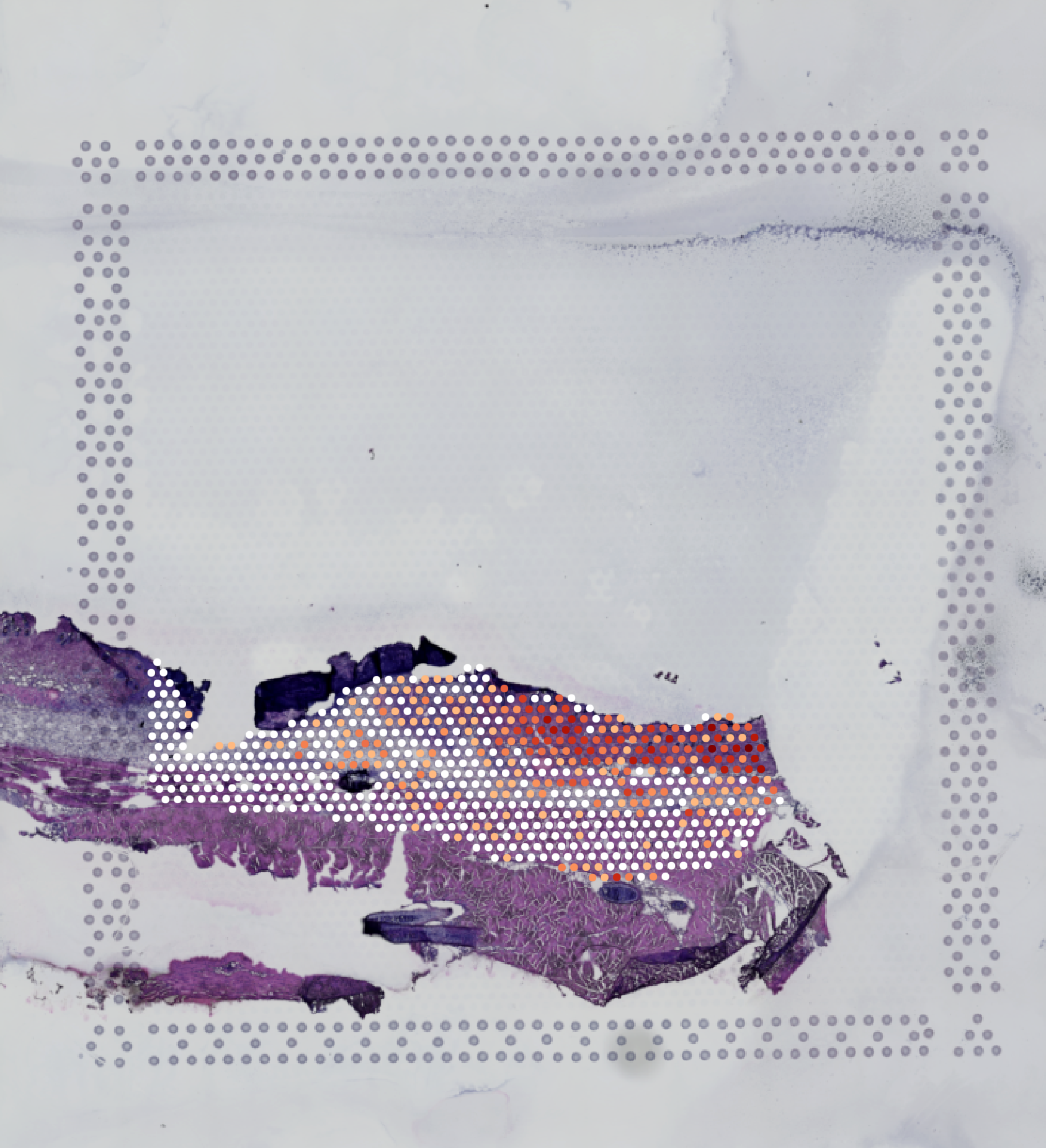

Supplement: Supplementary file 16 — Source data Fig. 3 [file 44319_2024_322_MOESM16_ESM.zip › SD figure 3/Figure3B/Day 3/C4b.tif]

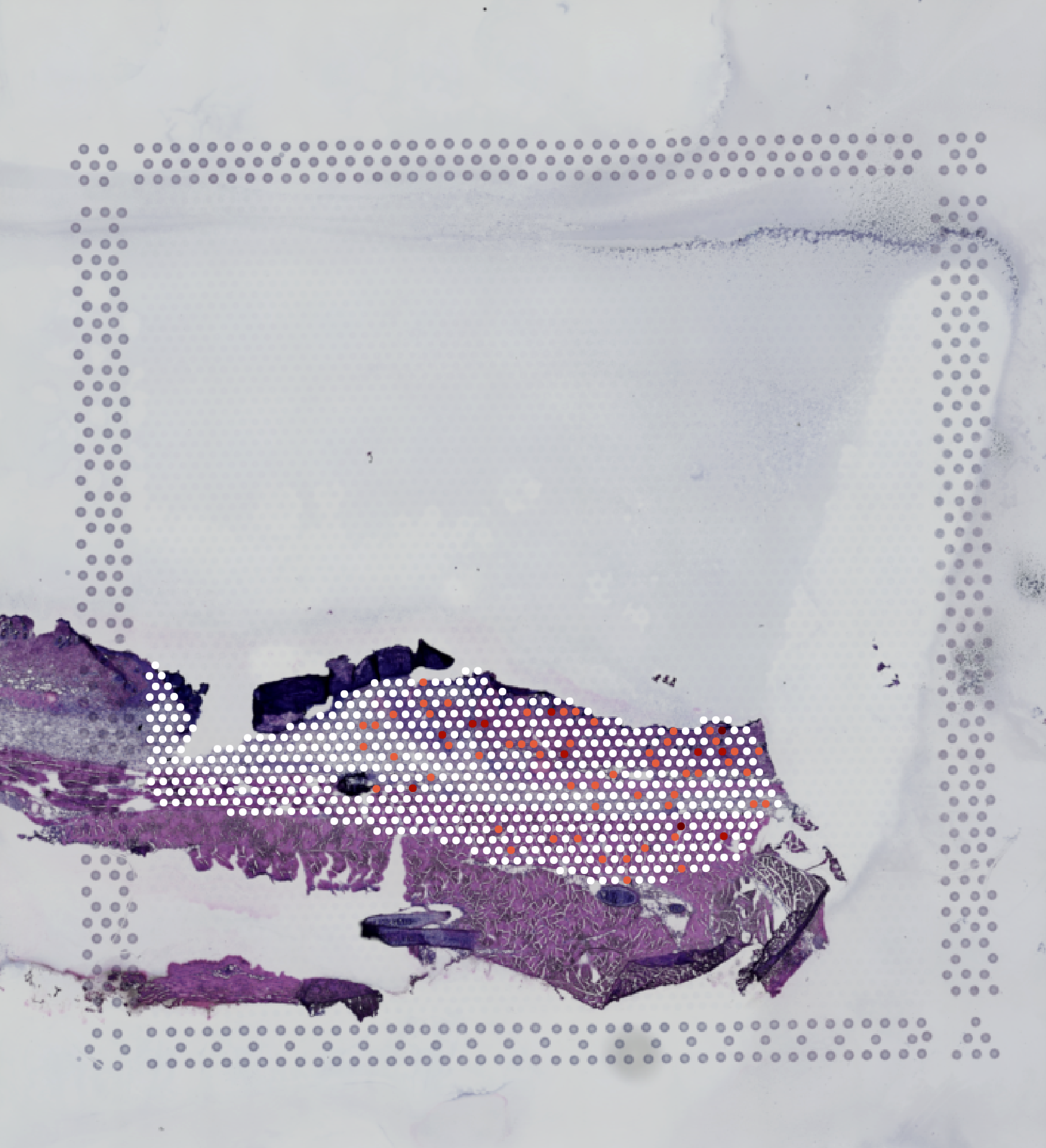

Supplement: Supplementary file 16 — Source data Fig. 3 [file 44319_2024_322_MOESM16_ESM.zip › SD figure 3/Figure3B/Day 3/Ccl11.tif]

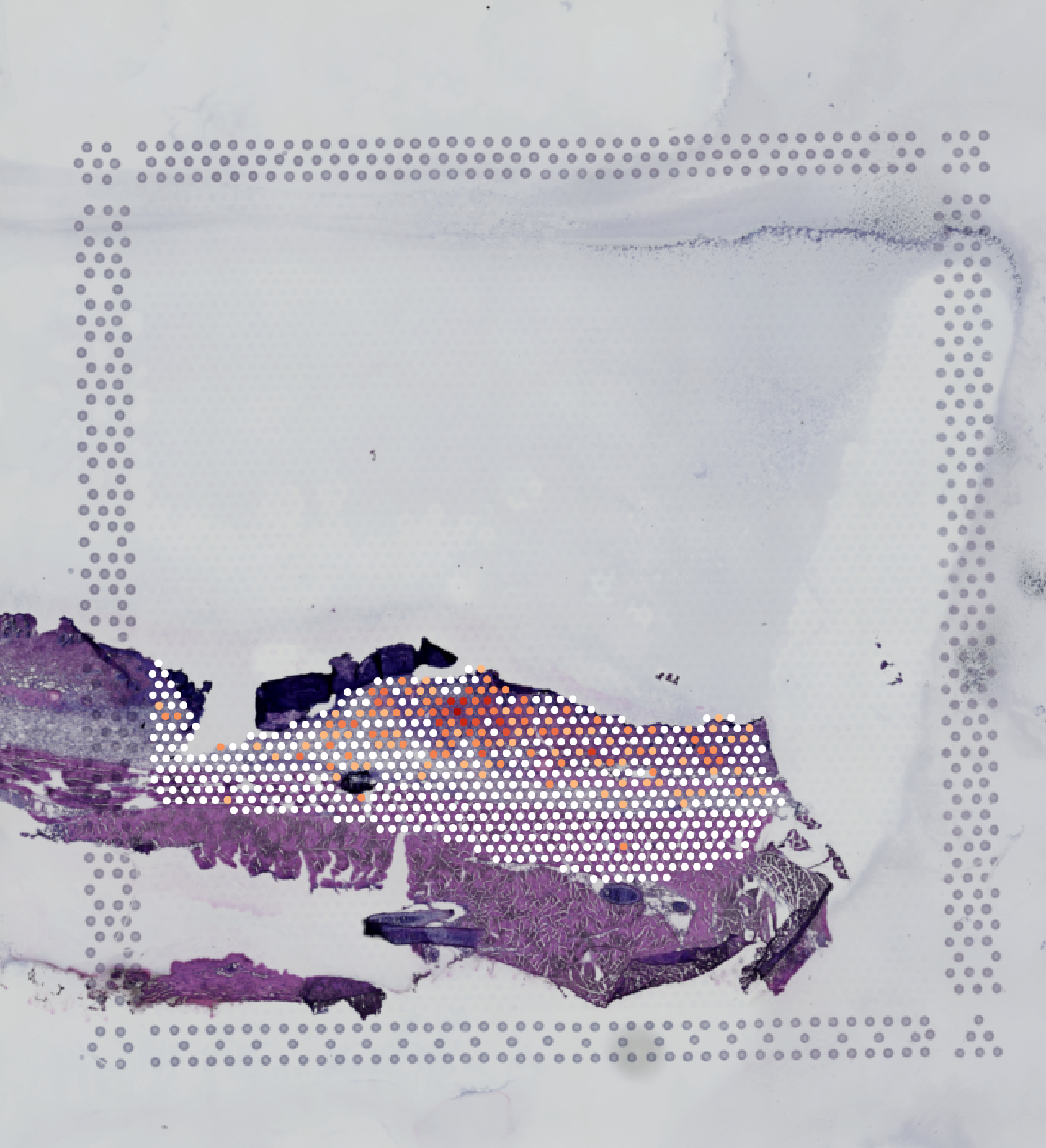

Supplement: Supplementary file 16 — Source data Fig. 3 [file 44319_2024_322_MOESM16_ESM.zip › SD figure 3/Figure3B/Day 3/Ccl7.tif]

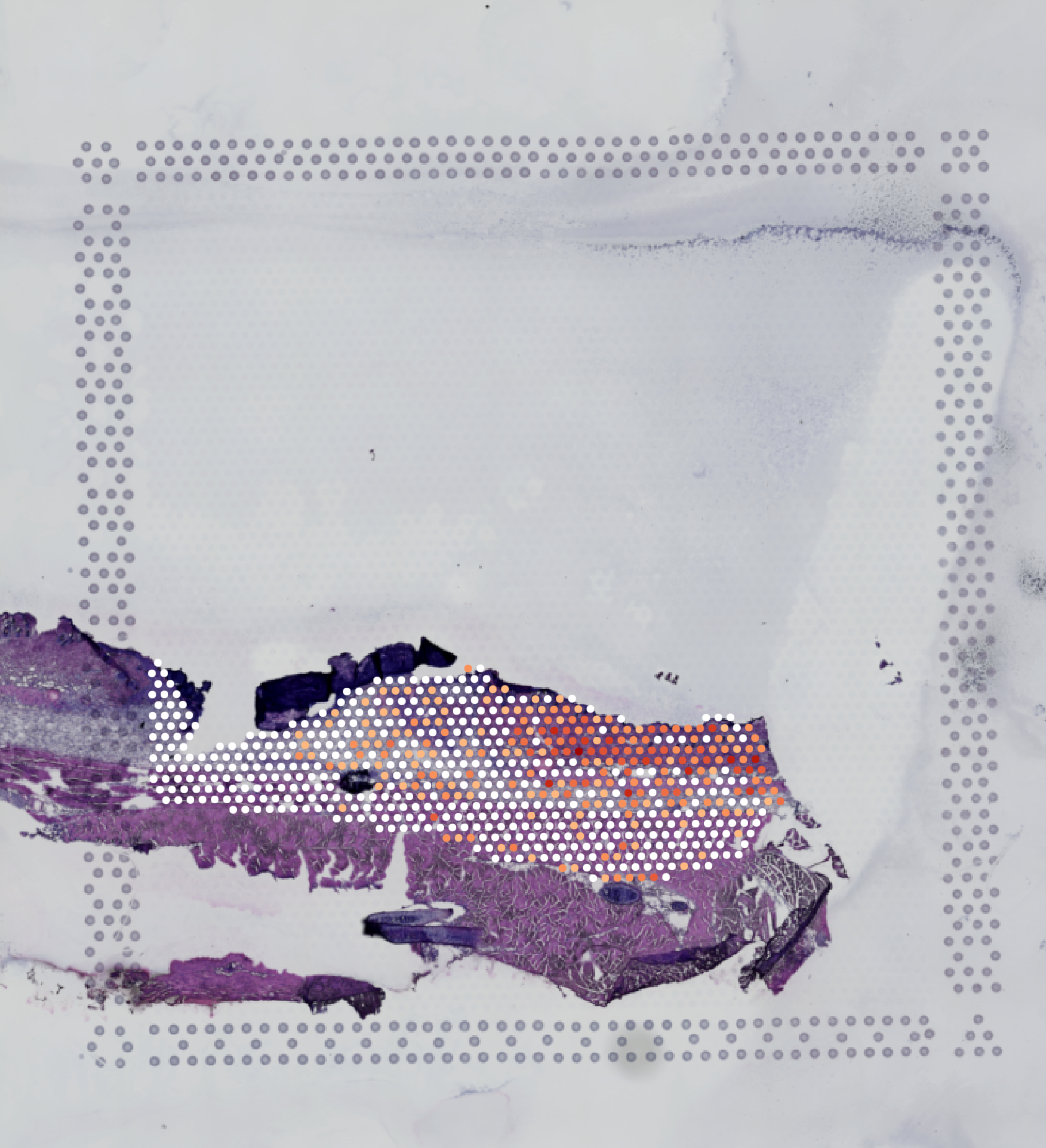

Supplement: Supplementary file 16 — Source data Fig. 3 [file 44319_2024_322_MOESM16_ESM.zip › SD figure 3/Figure3B/Day 3/Clec3b.tif]

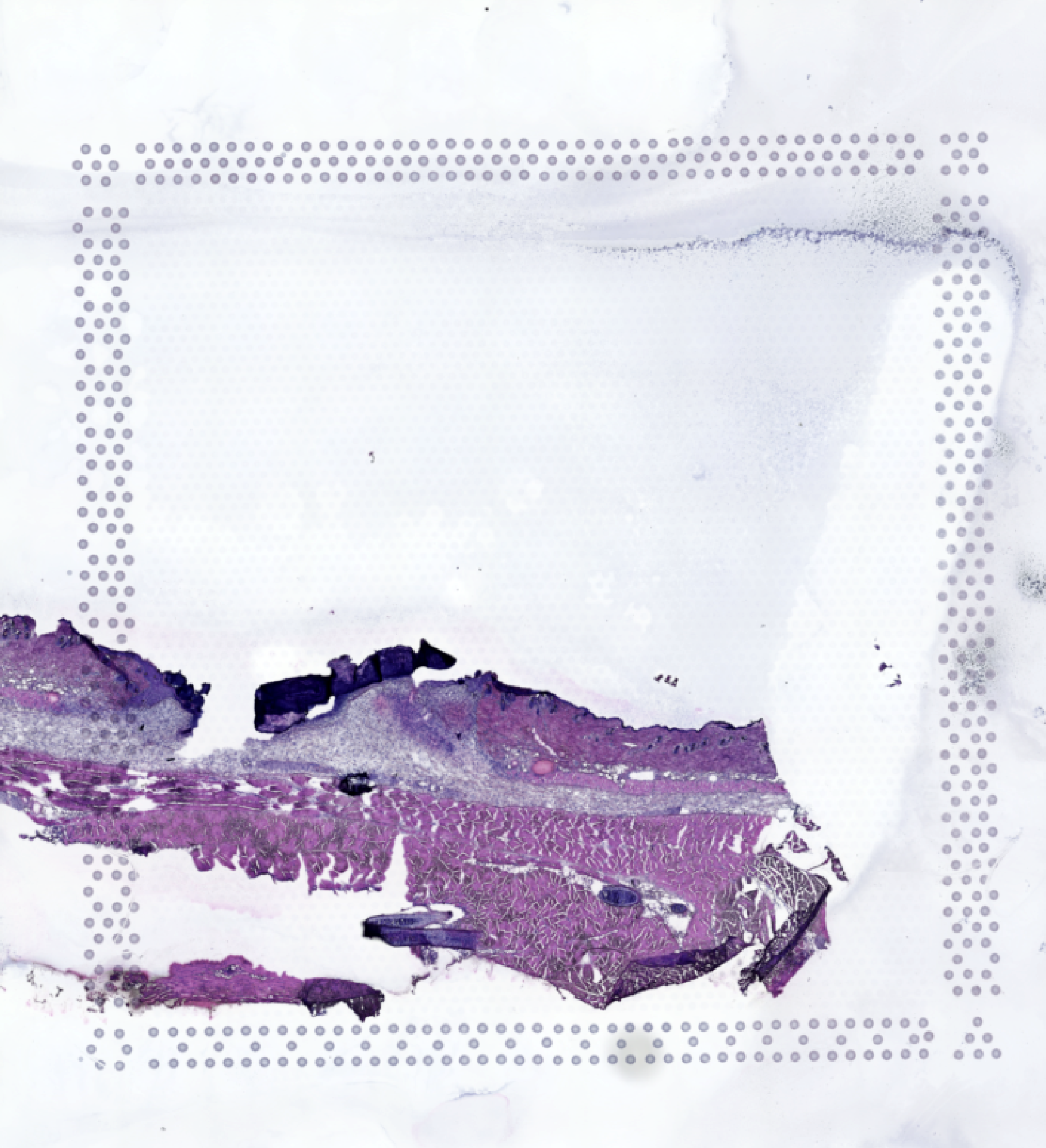

Supplement: Supplementary file 16 — Source data Fig. 3 [file 44319_2024_322_MOESM16_ESM.zip › SD figure 3/Figure3B/Day 3/HE.tif]

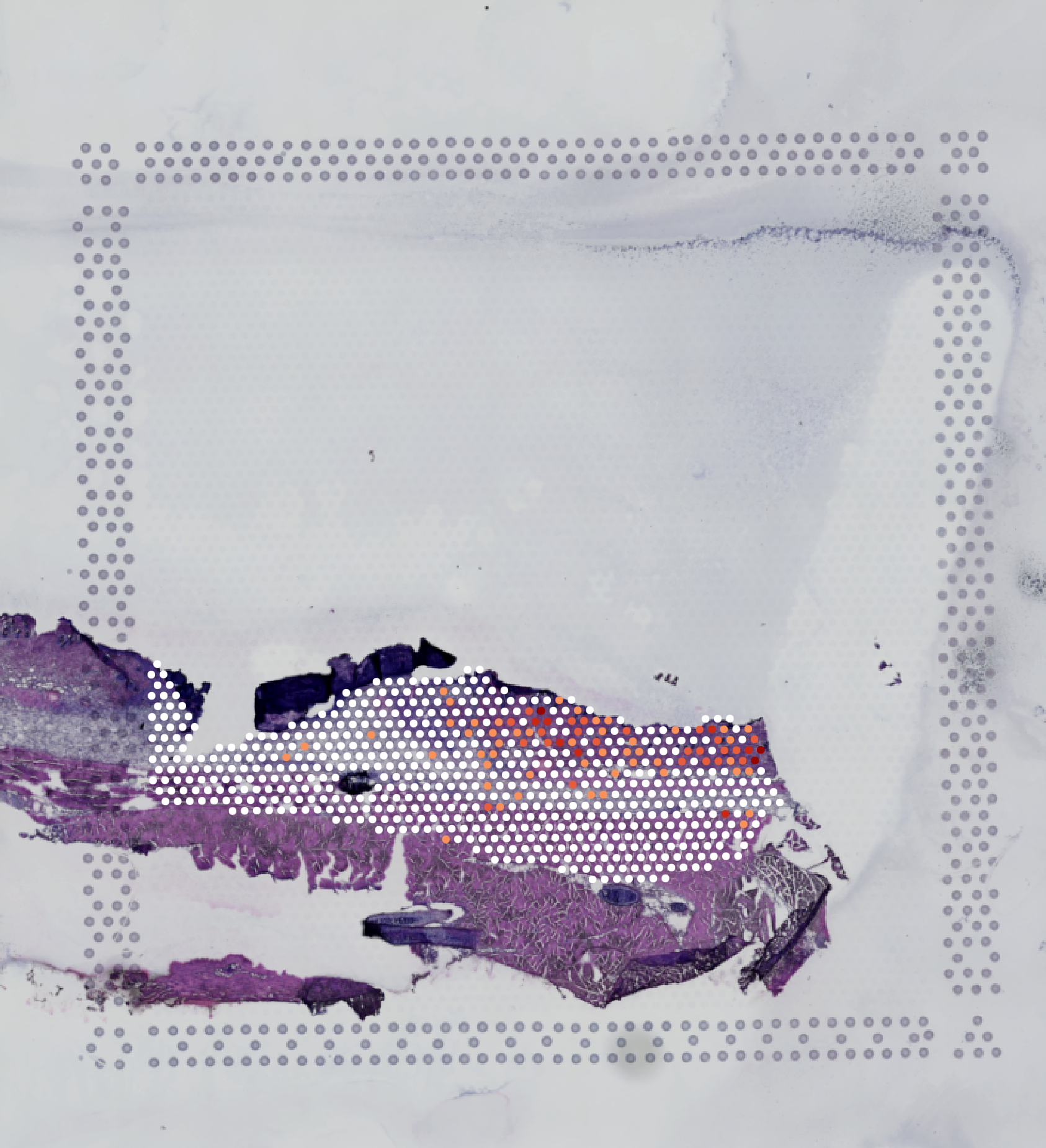

Supplement: Supplementary file 16 — Source data Fig. 3 [file 44319_2024_322_MOESM16_ESM.zip › SD figure 3/Figure3B/Day 3/Itgbl1.tif]

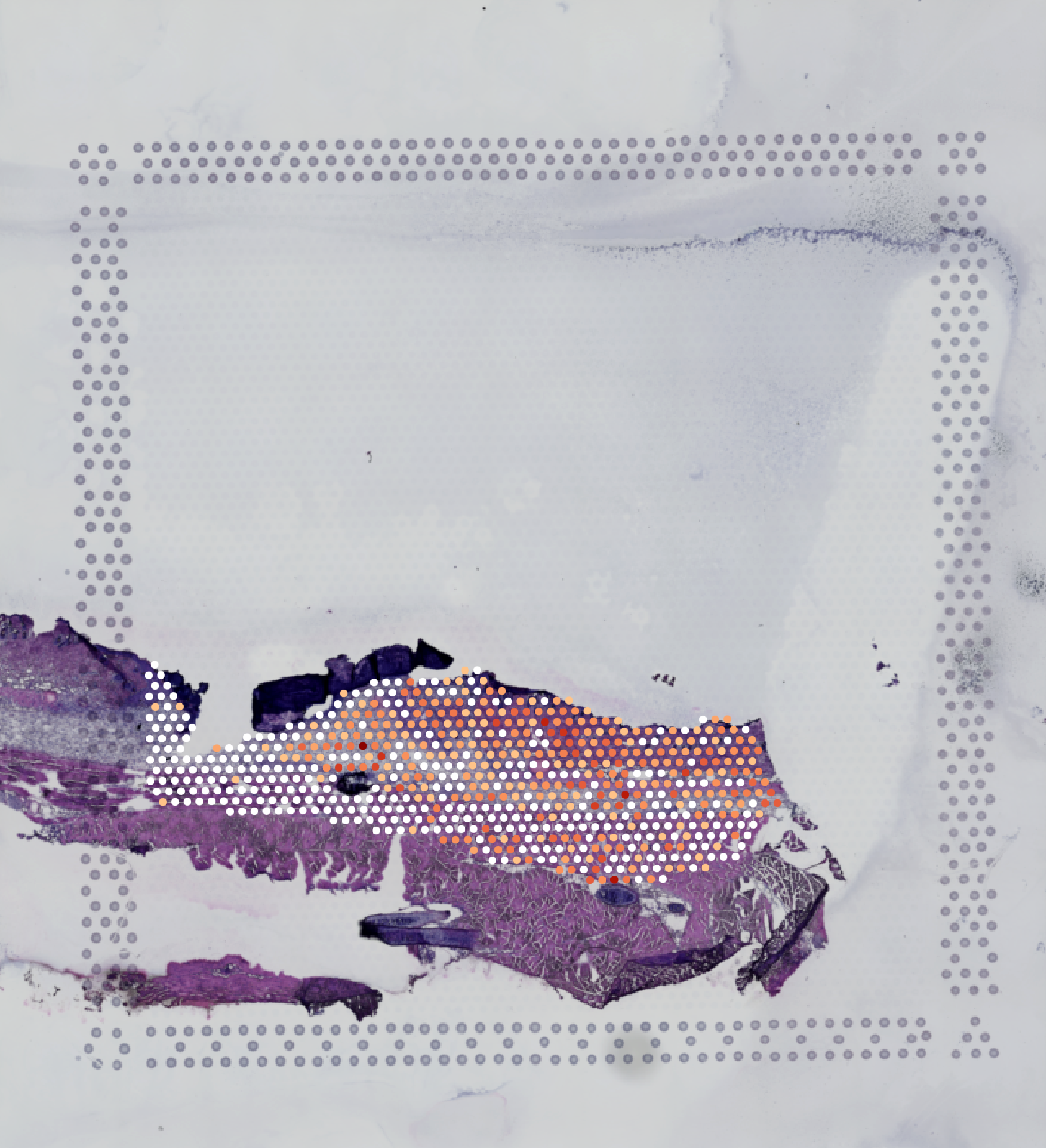

Supplement: Supplementary file 16 — Source data Fig. 3 [file 44319_2024_322_MOESM16_ESM.zip › SD figure 3/Figure3B/Day 3/Lrrc17.tif]

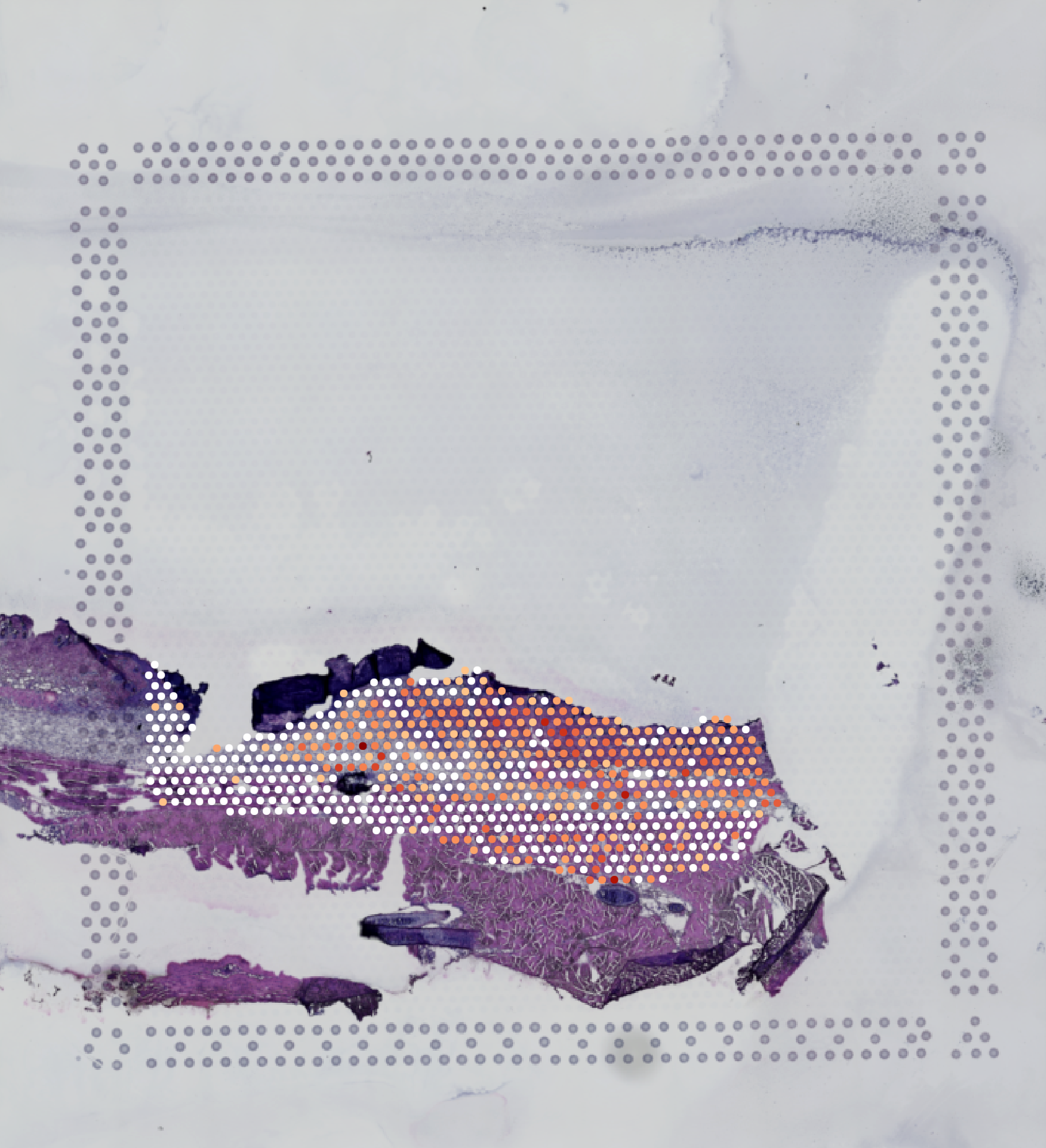

Supplement: Supplementary file 16 — Source data Fig. 3 [file 44319_2024_322_MOESM16_ESM.zip › SD figure 3/Figure3B/Day 3/Pi16.tif]

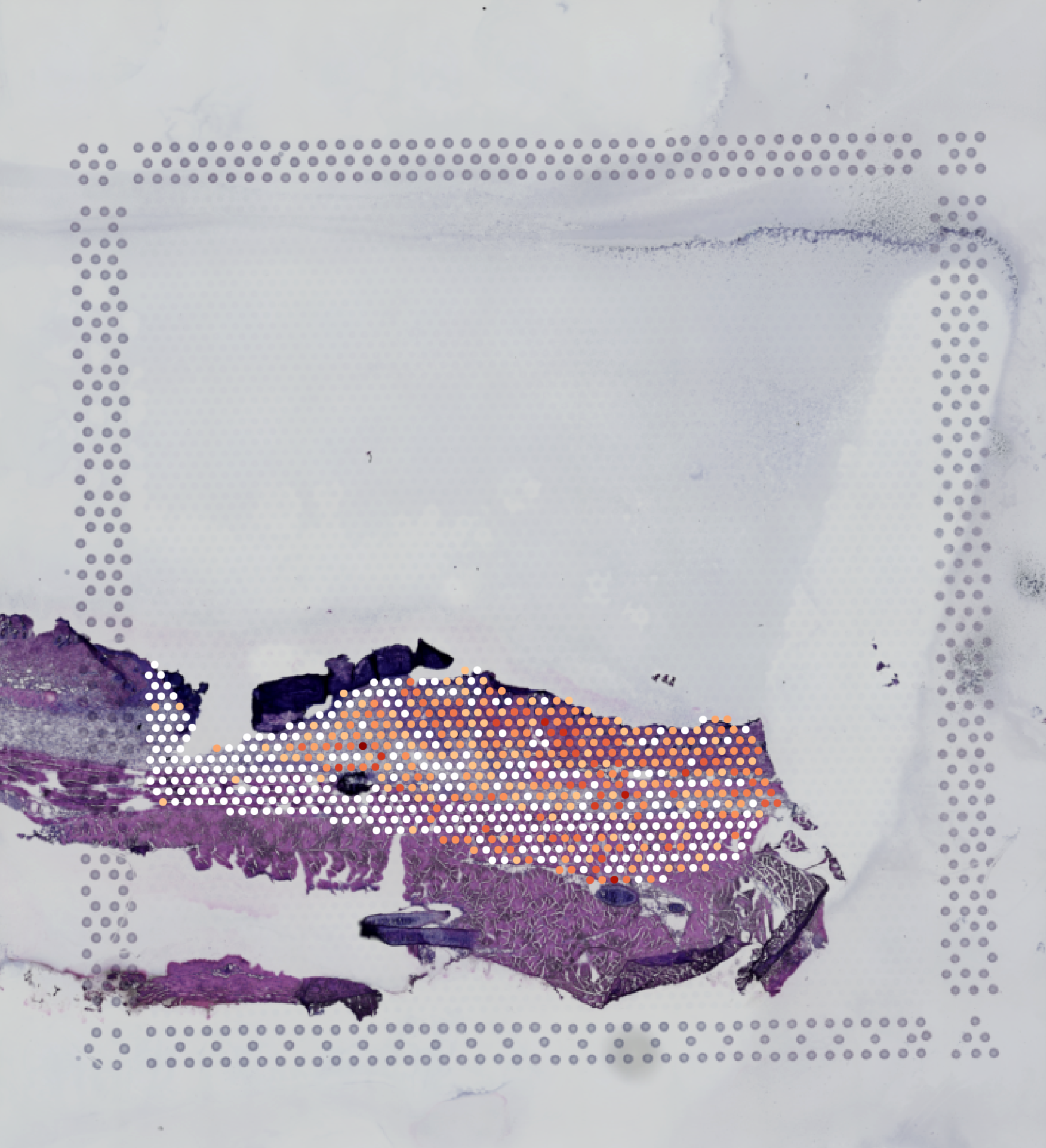

Supplement: Supplementary file 16 — Source data Fig. 3 [file 44319_2024_322_MOESM16_ESM.zip › SD figure 3/Figure3B/Day 3/Saa3.tif]

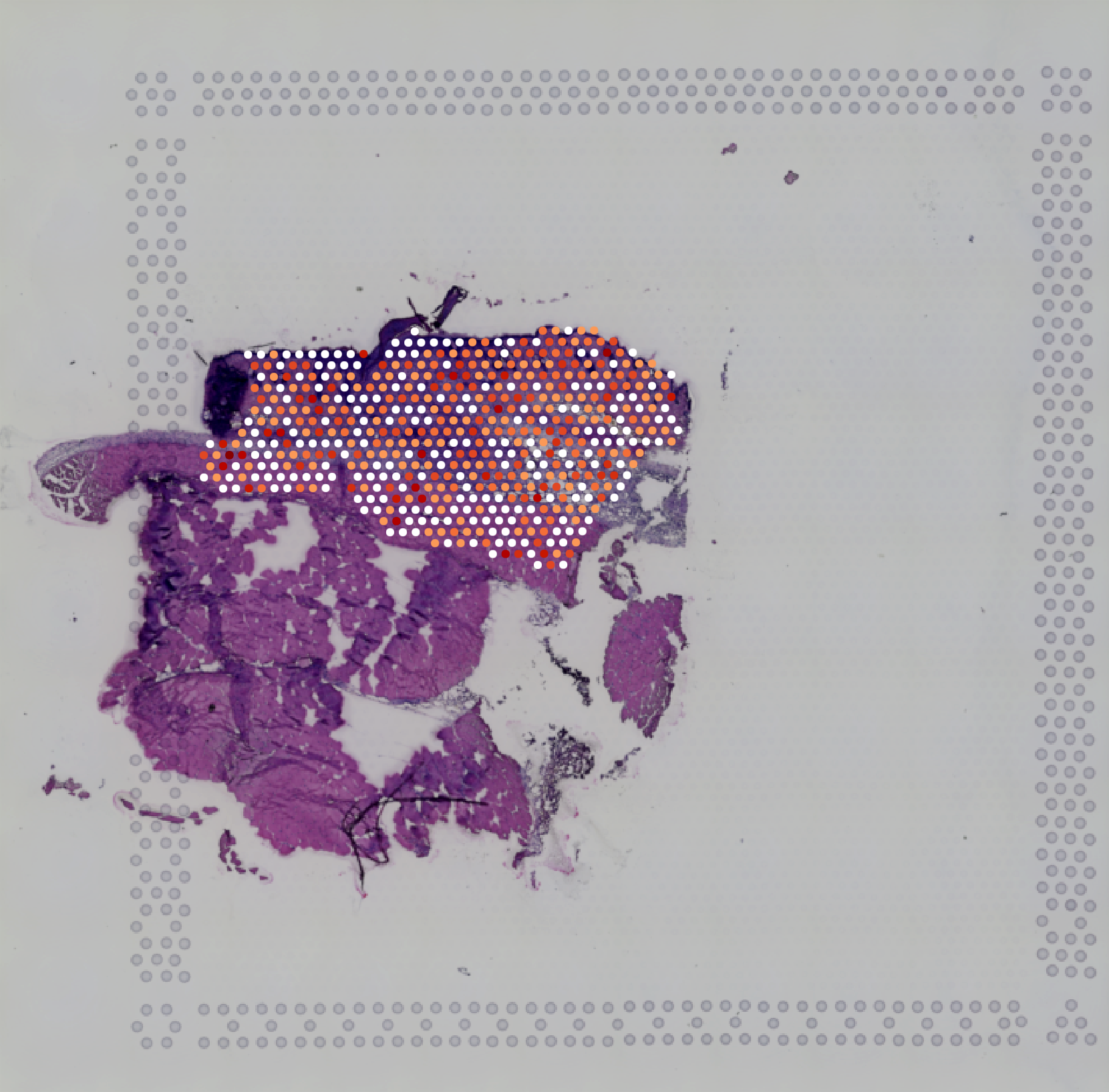

Supplement: Supplementary file 16 — Source data Fig. 3 [file 44319_2024_322_MOESM16_ESM.zip › SD figure 3/Figure3B/Day 7/C3.tif]

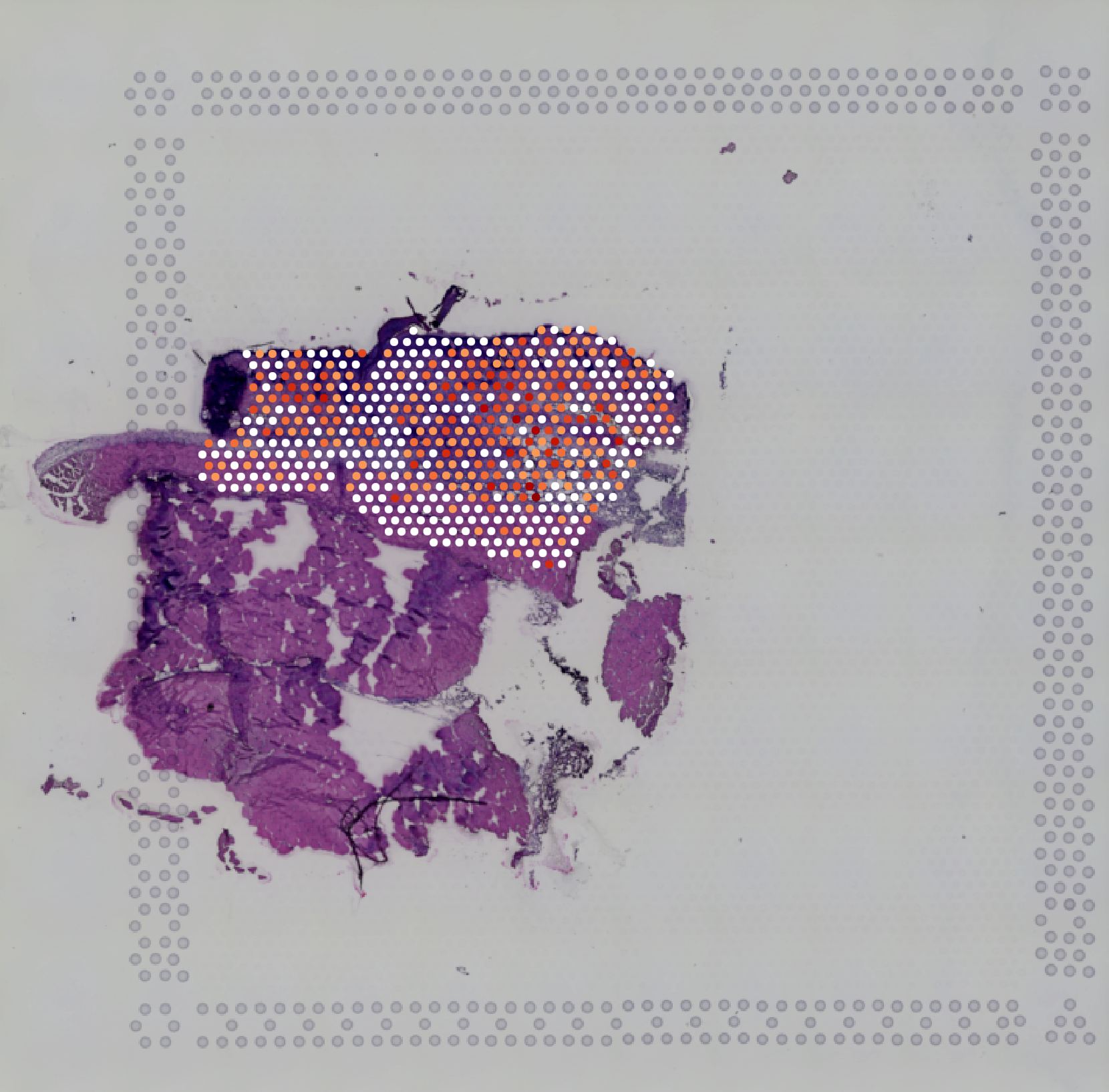

Supplement: Supplementary file 16 — Source data Fig. 3 [file 44319_2024_322_MOESM16_ESM.zip › SD figure 3/Figure3B/Day 7/C4b.tif]

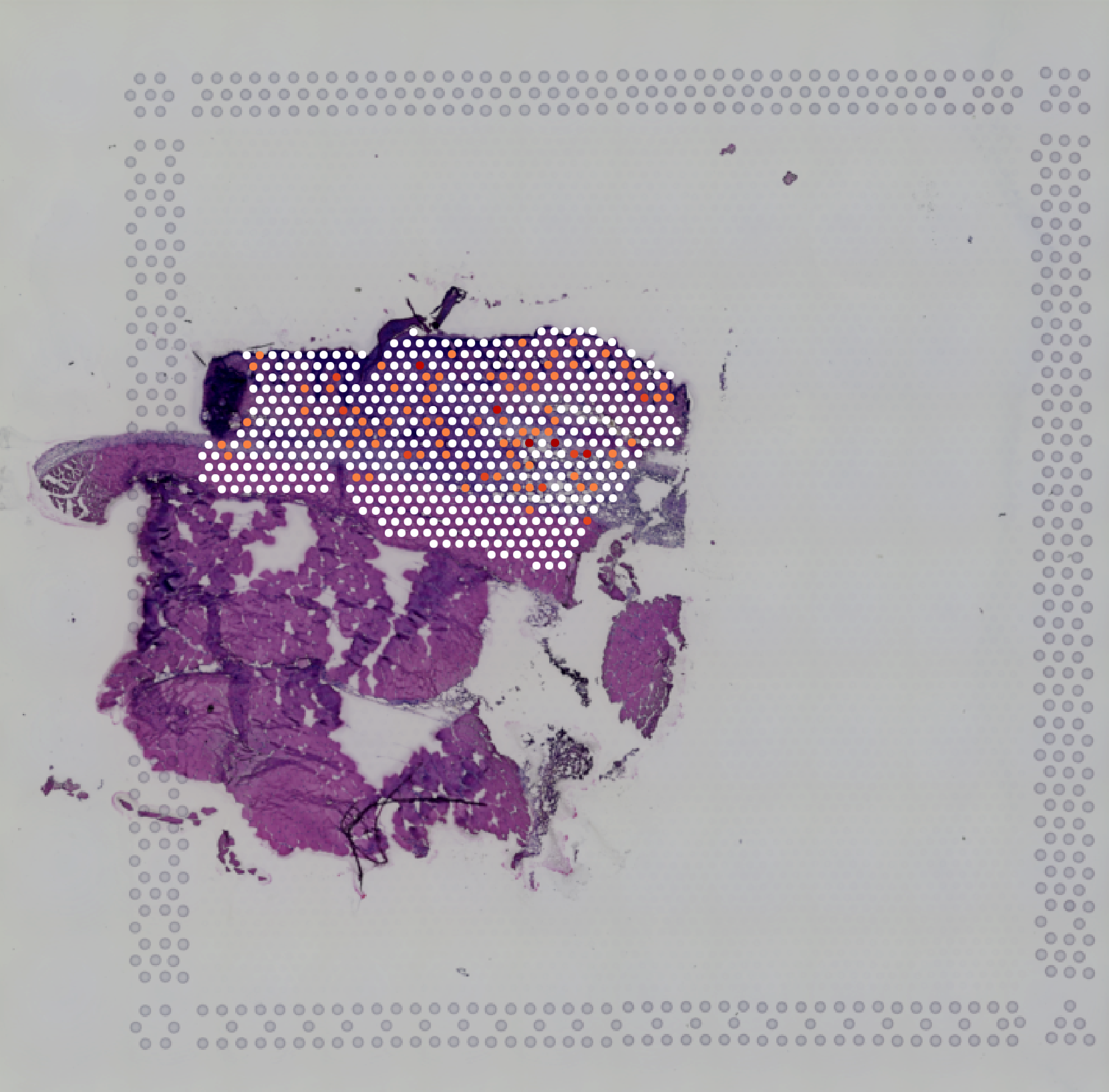

Supplement: Supplementary file 16 — Source data Fig. 3 [file 44319_2024_322_MOESM16_ESM.zip › SD figure 3/Figure3B/Day 7/Ccl11.tif]

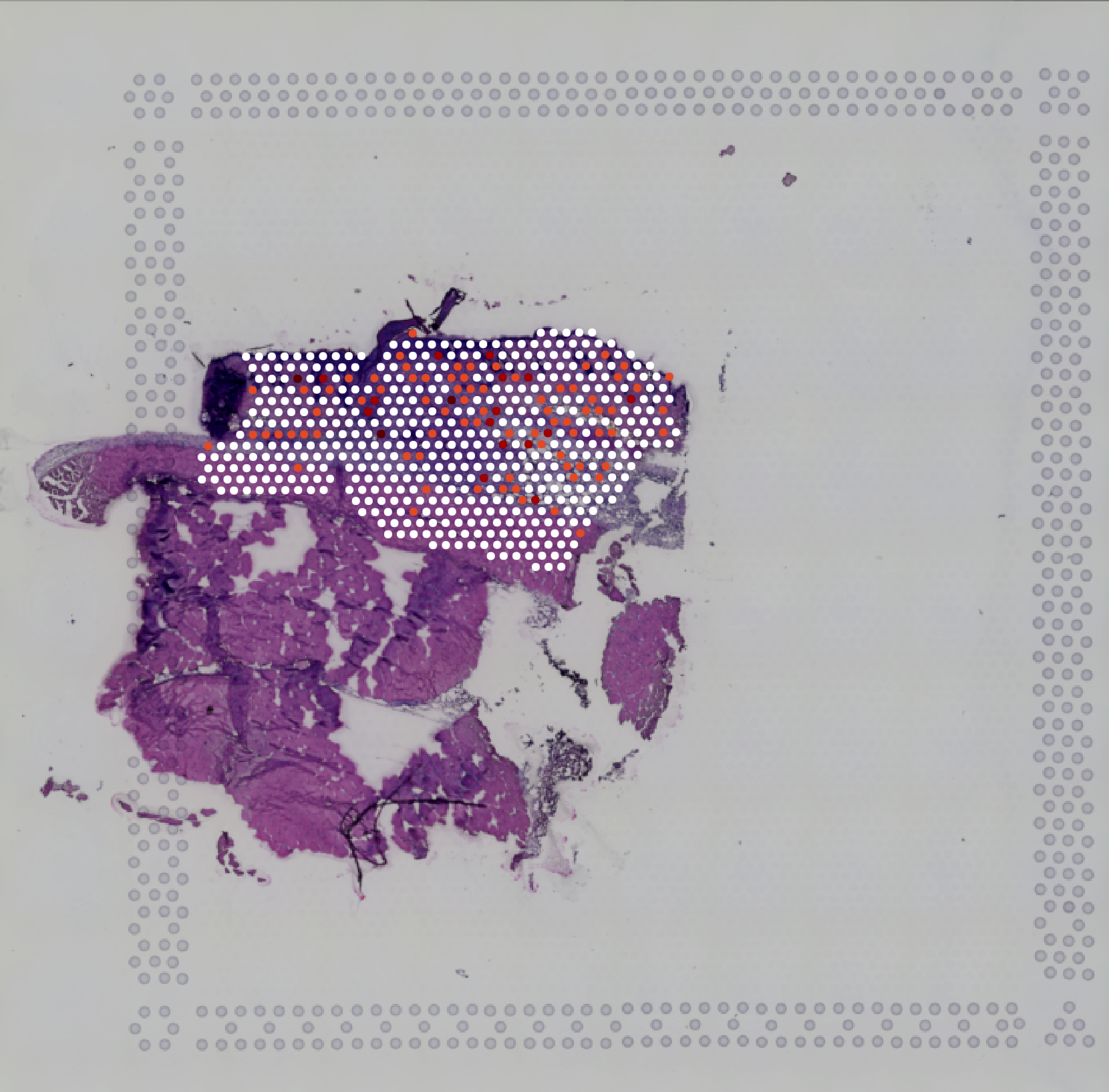

Supplement: Supplementary file 16 — Source data Fig. 3 [file 44319_2024_322_MOESM16_ESM.zip › SD figure 3/Figure3B/Day 7/Ccl7.tif]

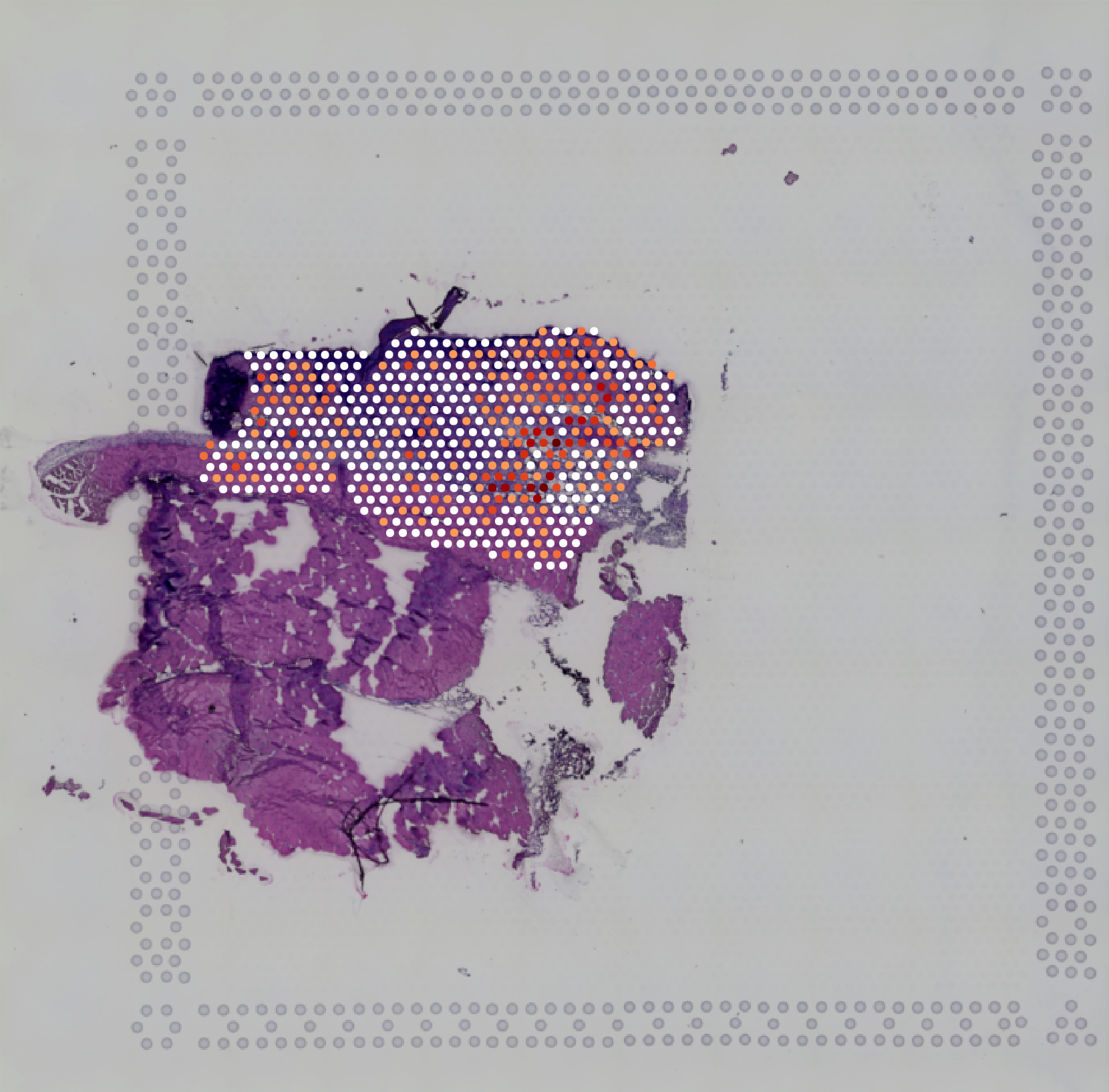

Supplement: Supplementary file 16 — Source data Fig. 3 [file 44319_2024_322_MOESM16_ESM.zip › SD figure 3/Figure3B/Day 7/Clec3b.tif]

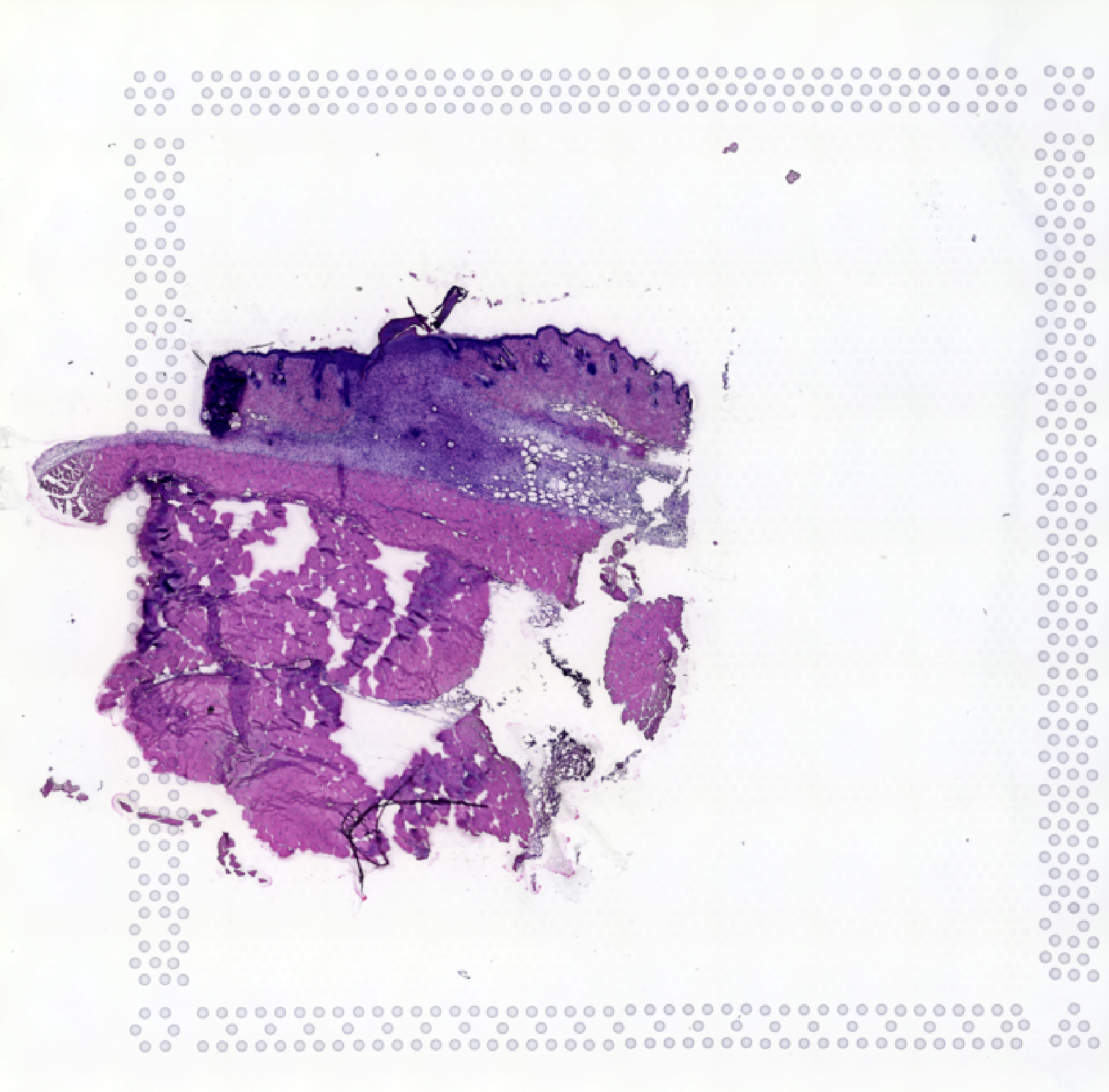

Supplement: Supplementary file 16 — Source data Fig. 3 [file 44319_2024_322_MOESM16_ESM.zip › SD figure 3/Figure3B/Day 7/HE.tif]

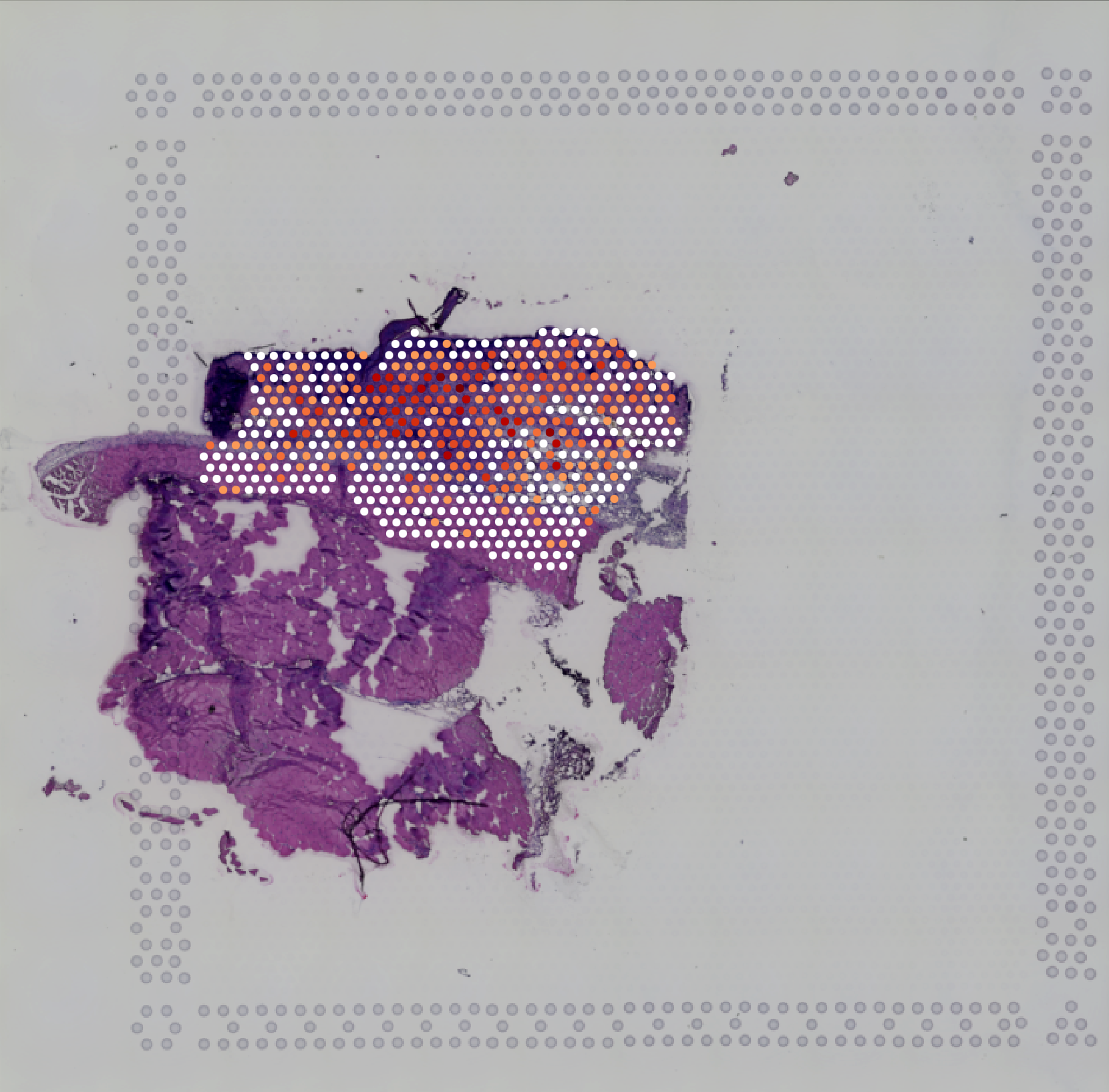

Supplement: Supplementary file 16 — Source data Fig. 3 [file 44319_2024_322_MOESM16_ESM.zip › SD figure 3/Figure3B/Day 7/Itgbl1.tif]

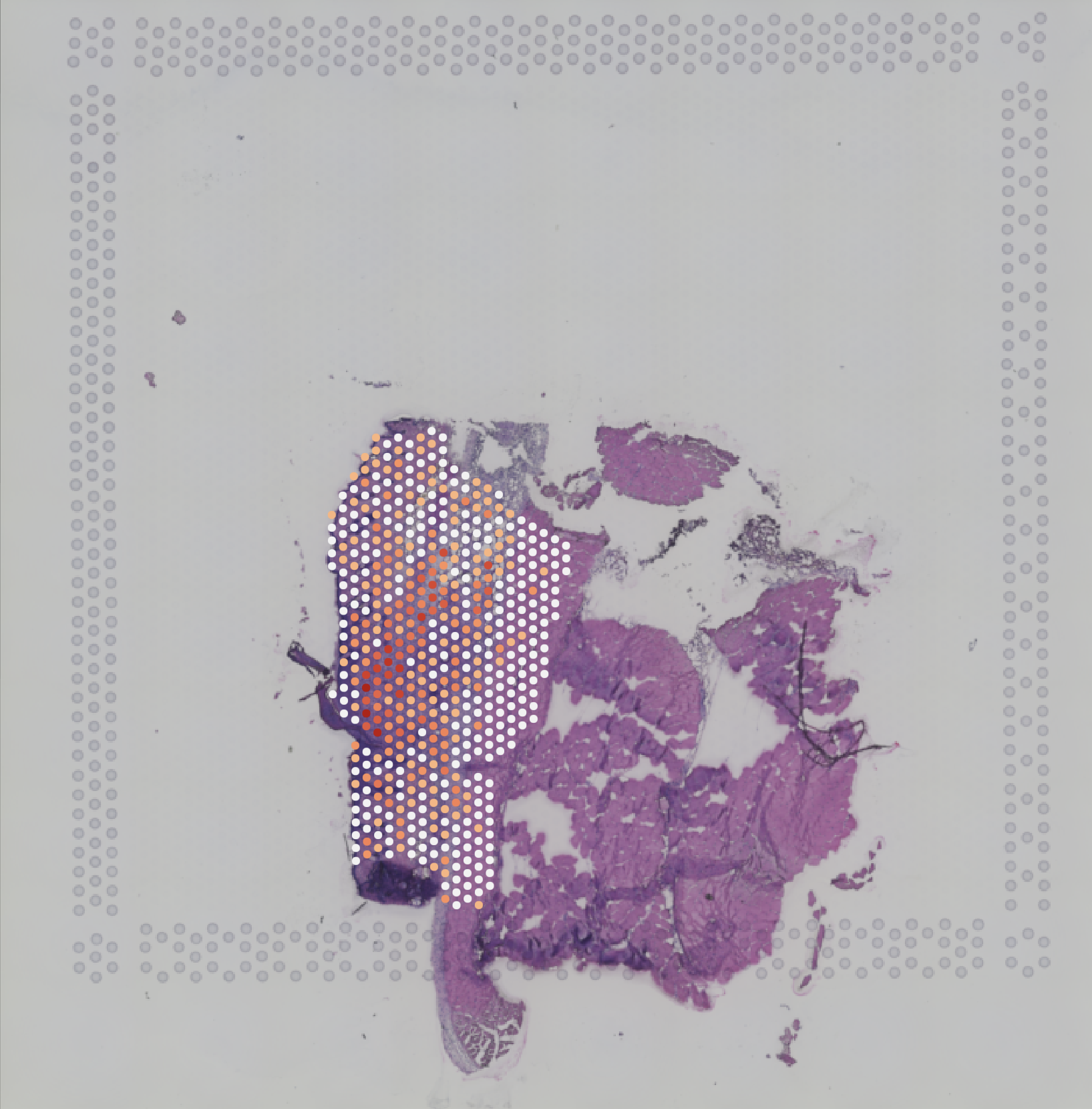

Supplement: Supplementary file 16 — Source data Fig. 3 [file 44319_2024_322_MOESM16_ESM.zip › SD figure 3/Figure3B/Day 7/Lrrc17.tif]

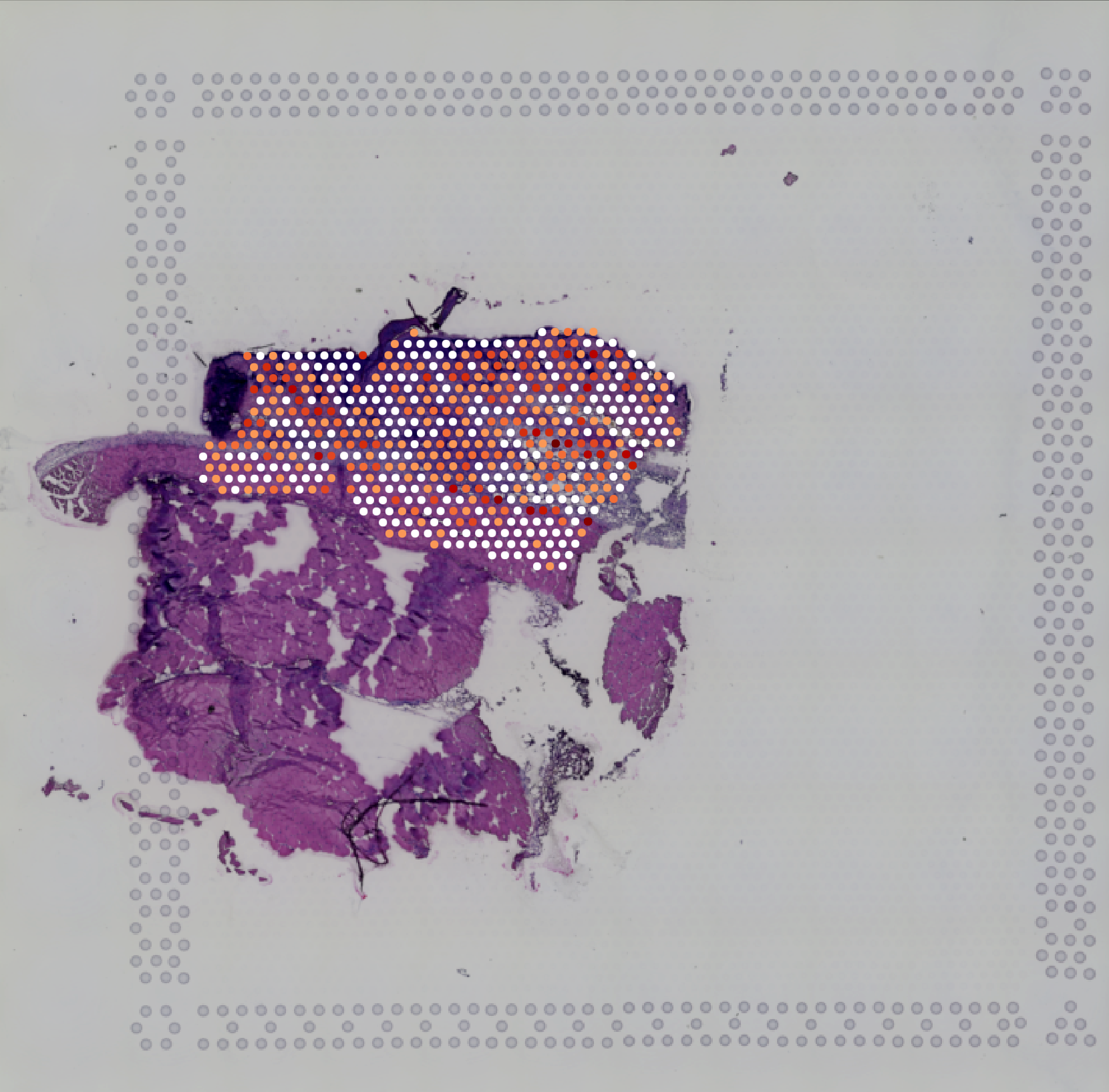

Supplement: Supplementary file 16 — Source data Fig. 3 [file 44319_2024_322_MOESM16_ESM.zip › SD figure 3/Figure3B/Day 7/Pi16.tif]

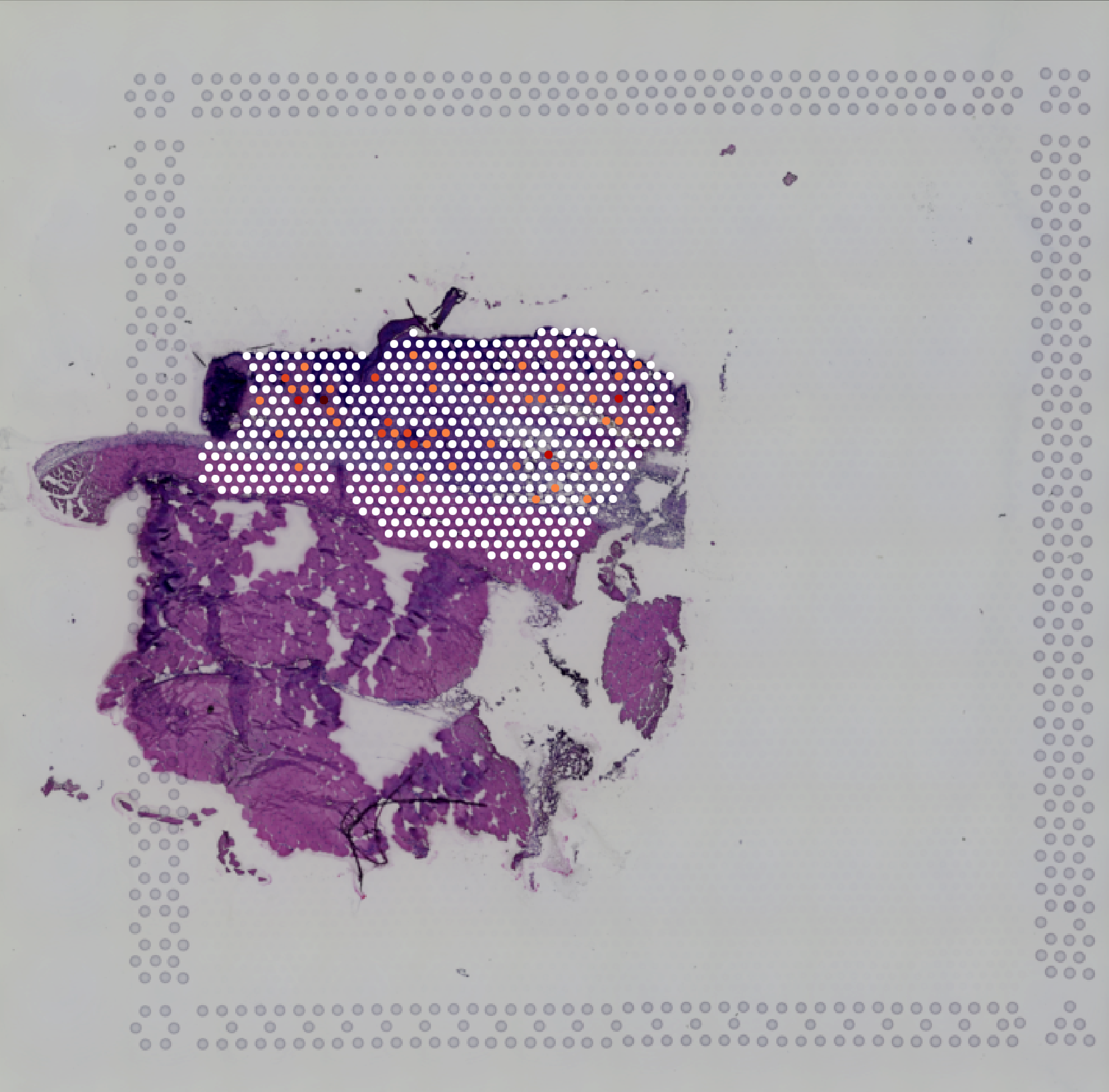

Supplement: Supplementary file 16 — Source data Fig. 3 [file 44319_2024_322_MOESM16_ESM.zip › SD figure 3/Figure3B/Day 7/Saa3.tif]

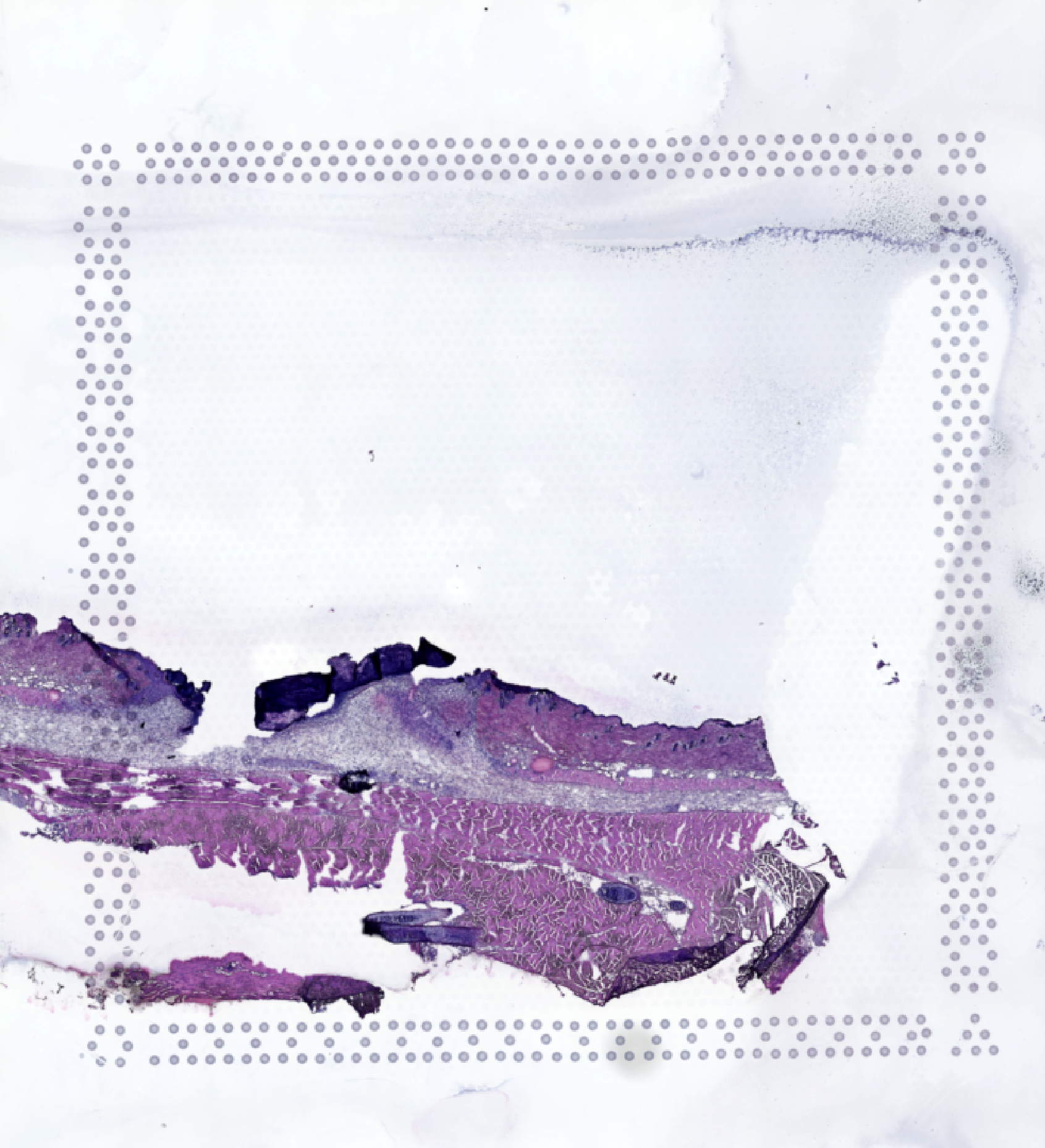

Supplement: Supplementary file 17 — Source data Fig. 4 [file 44319_2024_322_MOESM17_ESM.zip › SD figure 4/Figure4B/HE.tif]

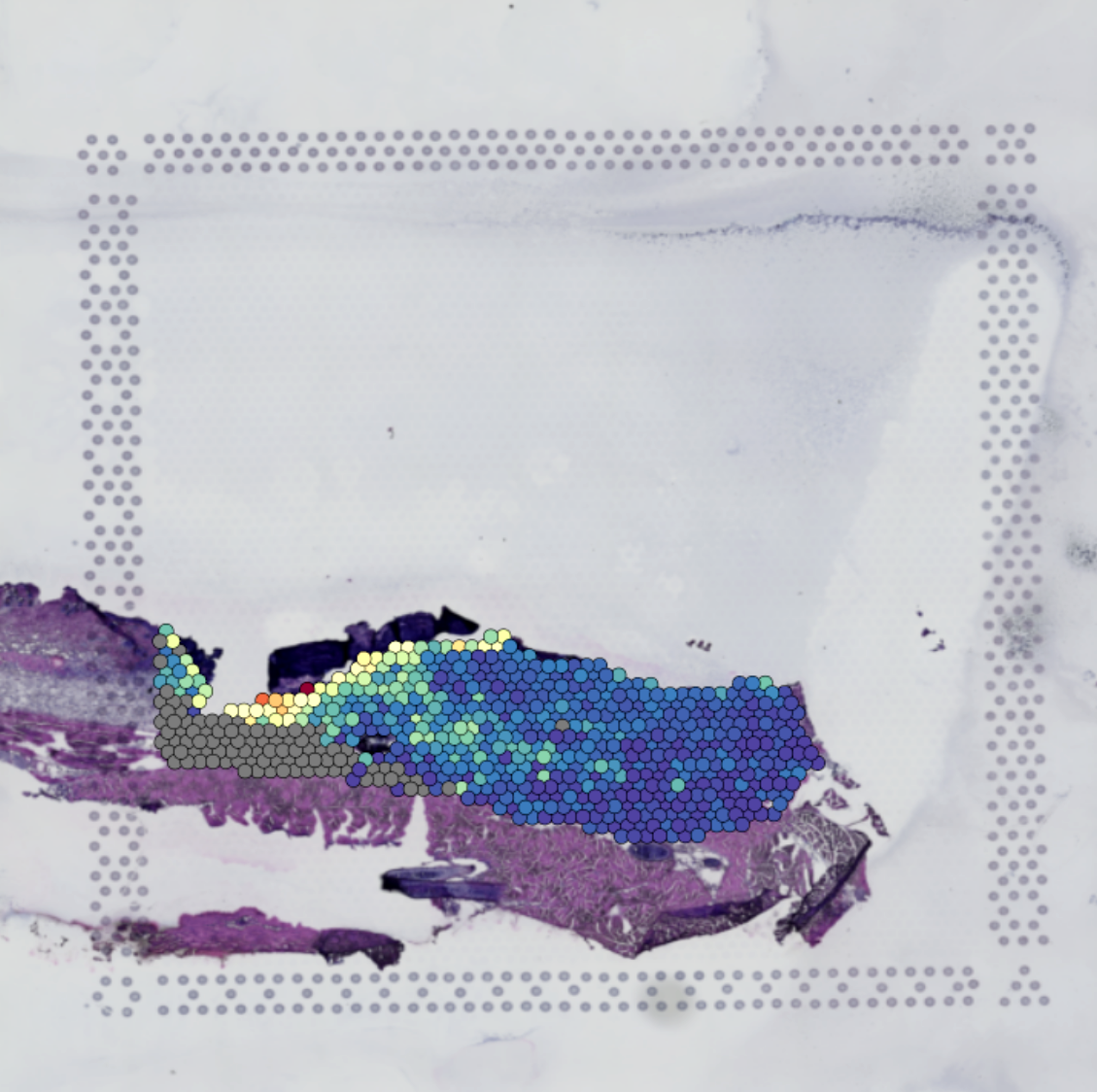

Supplement: Supplementary file 17 — Source data Fig. 4 [file 44319_2024_322_MOESM17_ESM.zip › SD figure 4/Figure4B/Neutrophil.tif]

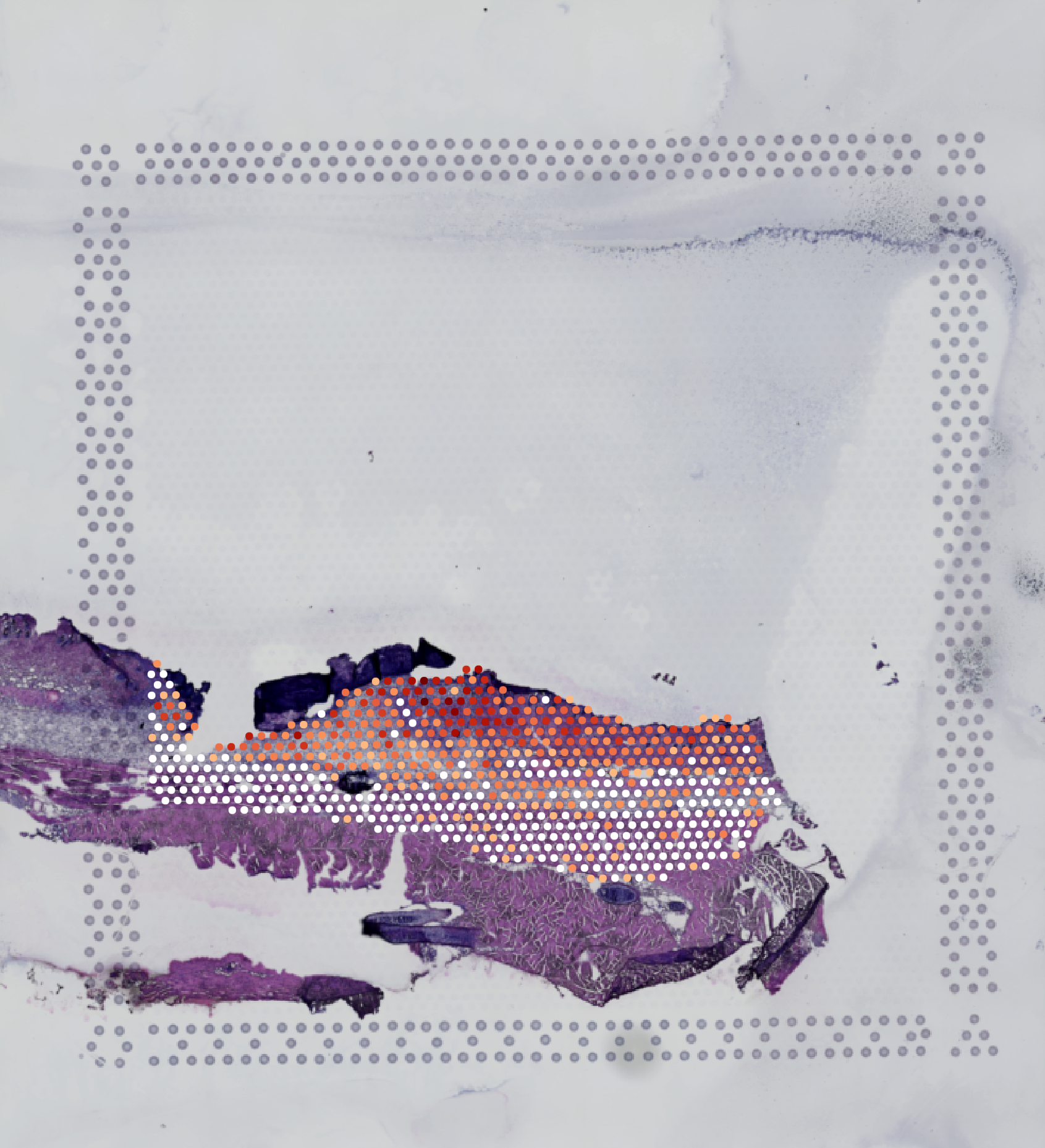

Supplement: Supplementary file 17 — Source data Fig. 4 [file 44319_2024_322_MOESM17_ESM.zip › SD figure 4/Figure4B/S100a9.tif]

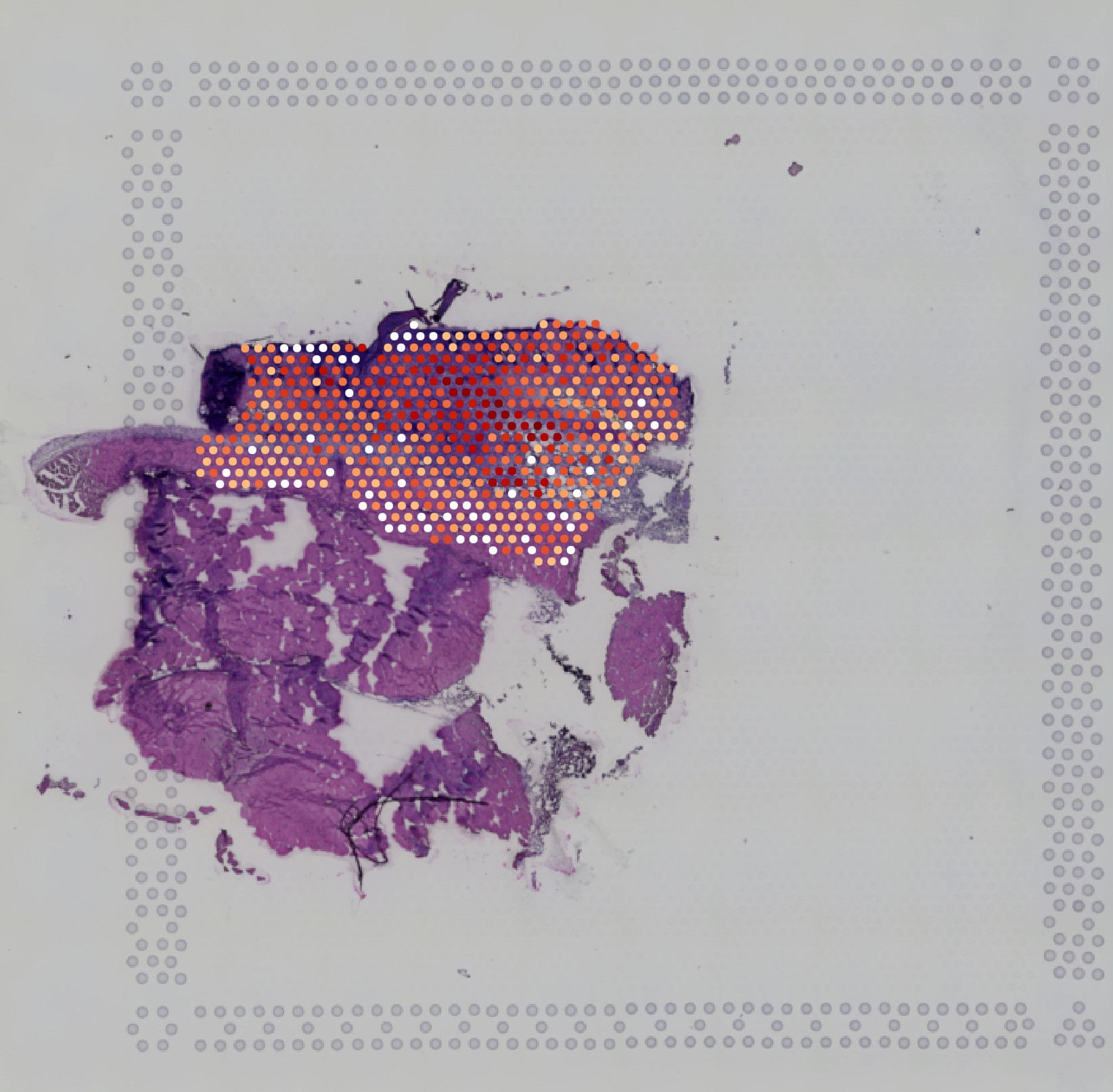

Supplement: Supplementary file 17 — Source data Fig. 4 [file 44319_2024_322_MOESM17_ESM.zip › SD figure 4/Figure4C/Dcn.tif]

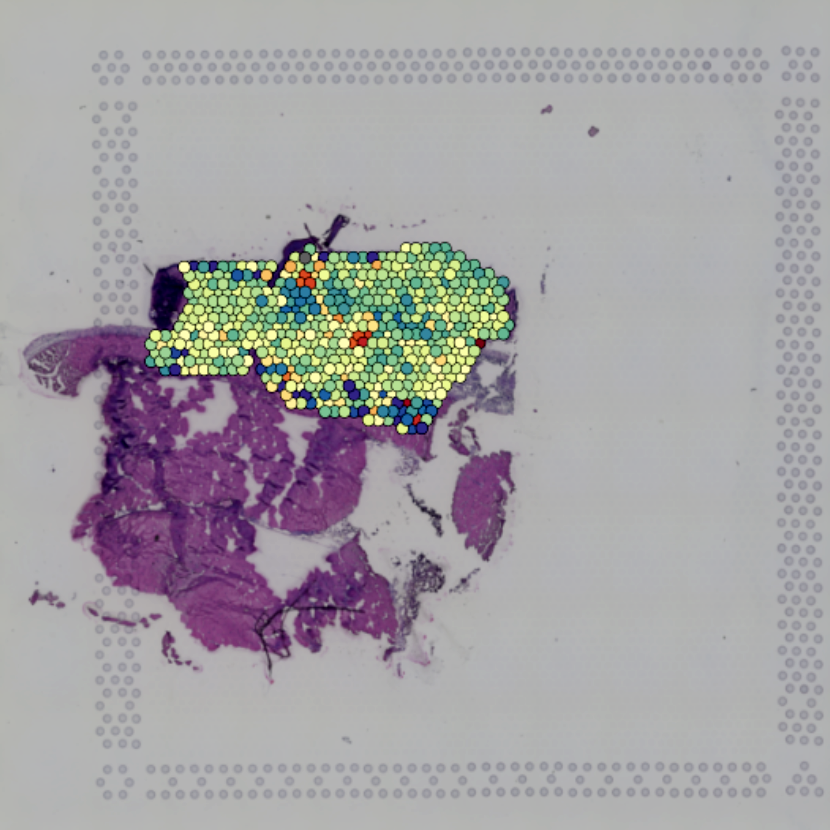

Supplement: Supplementary file 17 — Source data Fig. 4 [file 44319_2024_322_MOESM17_ESM.zip › SD figure 4/Figure4C/Endothelial cell.tif]

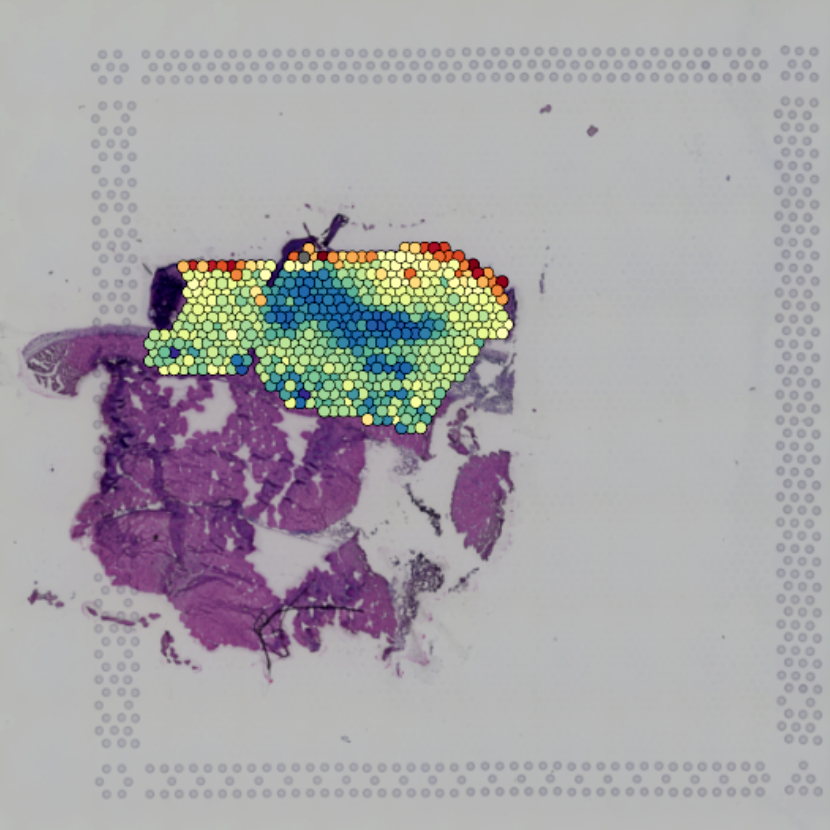

Supplement: Supplementary file 17 — Source data Fig. 4 [file 44319_2024_322_MOESM17_ESM.zip › SD figure 4/Figure4C/Epithelial cell.tif]

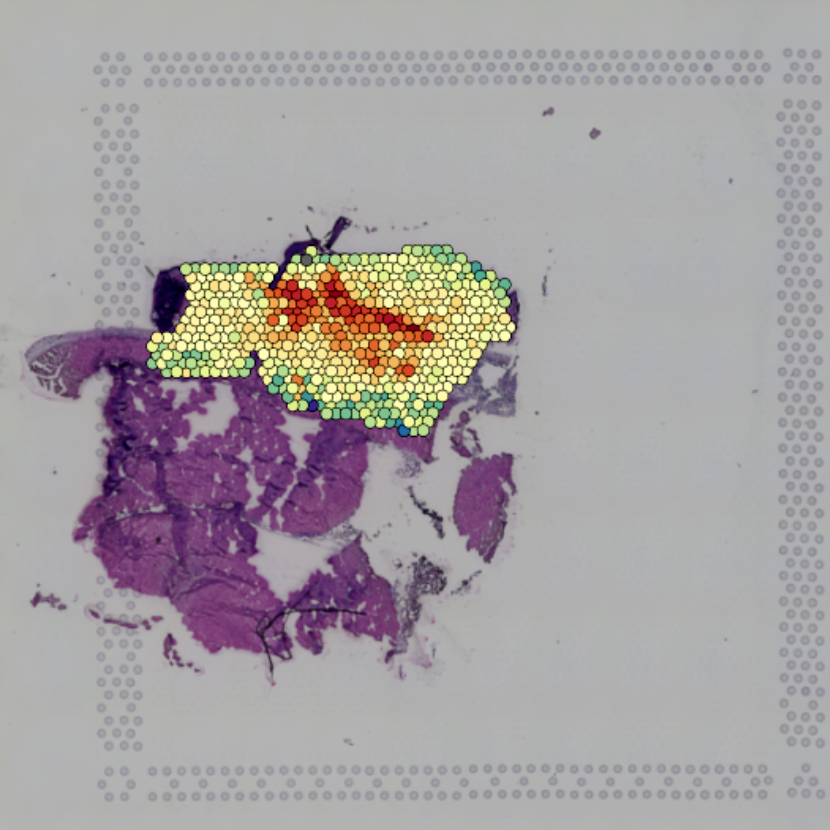

Supplement: Supplementary file 17 — Source data Fig. 4 [file 44319_2024_322_MOESM17_ESM.zip › SD figure 4/Figure4C/Fibroblast.tif]

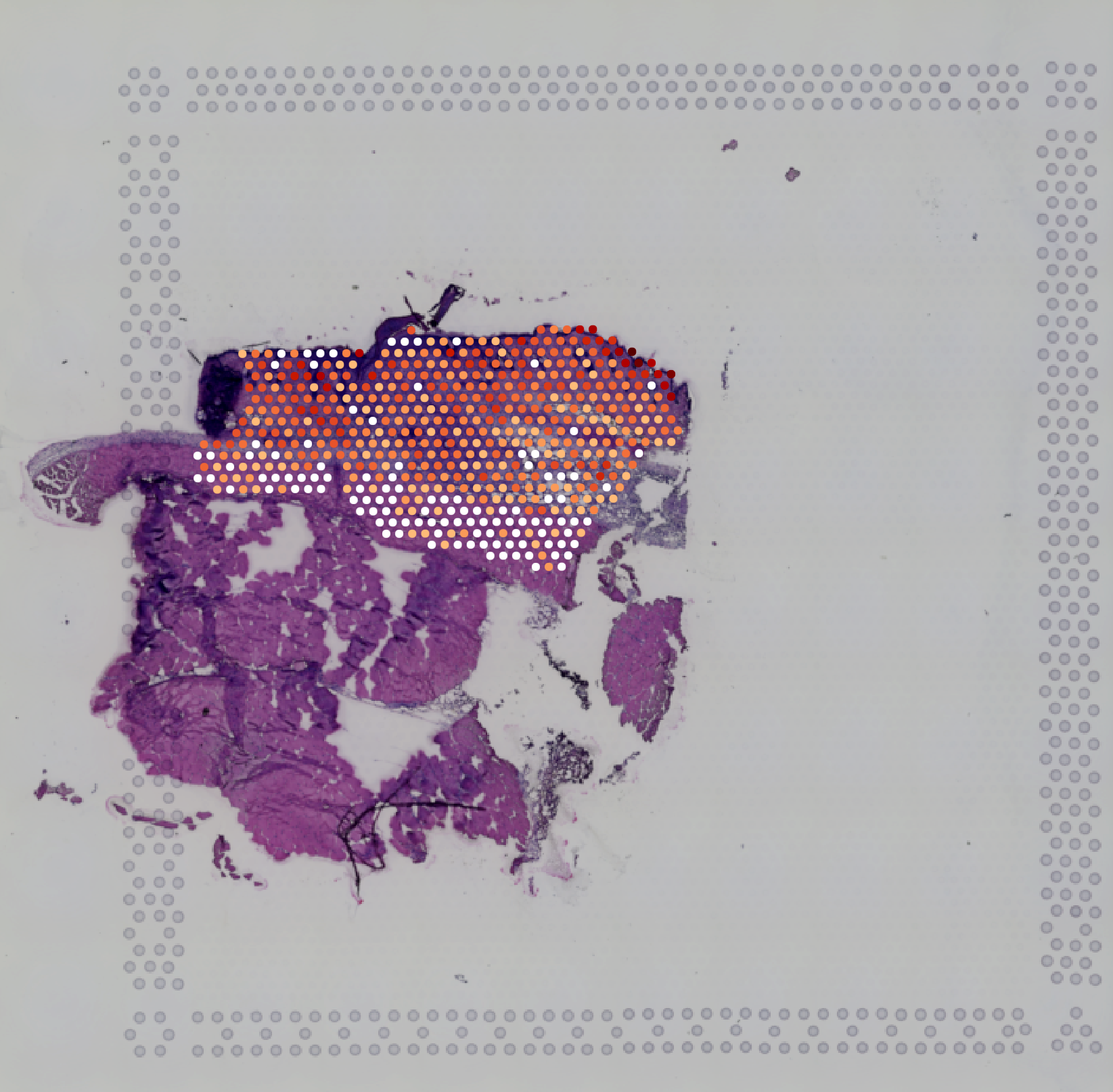

Supplement: Supplementary file 17 — Source data Fig. 4 [file 44319_2024_322_MOESM17_ESM.zip › SD figure 4/Figure4C/Krt10.tif]

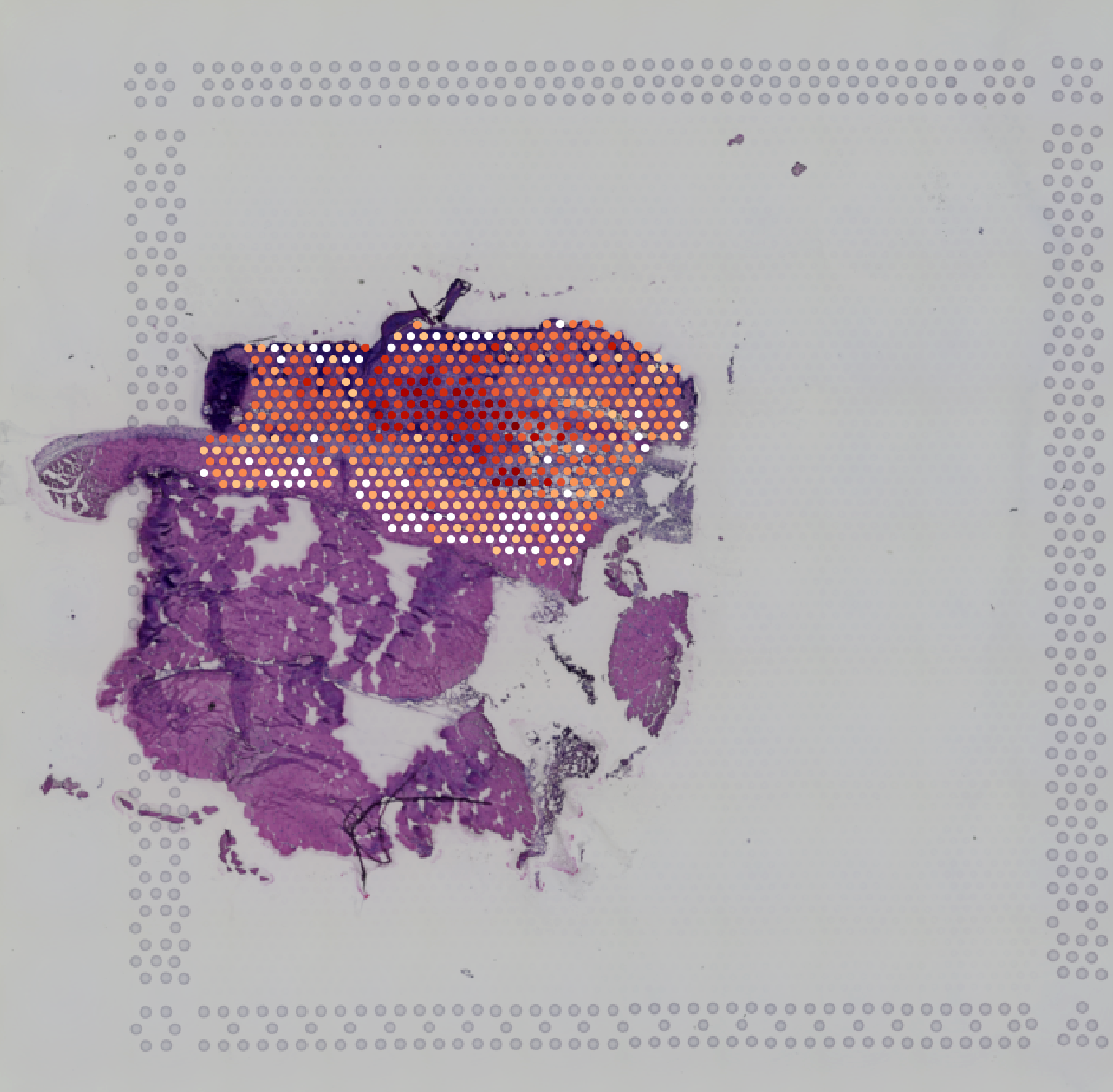

Supplement: Supplementary file 17 — Source data Fig. 4 [file 44319_2024_322_MOESM17_ESM.zip › SD figure 4/Figure4C/Lyz2.tif]

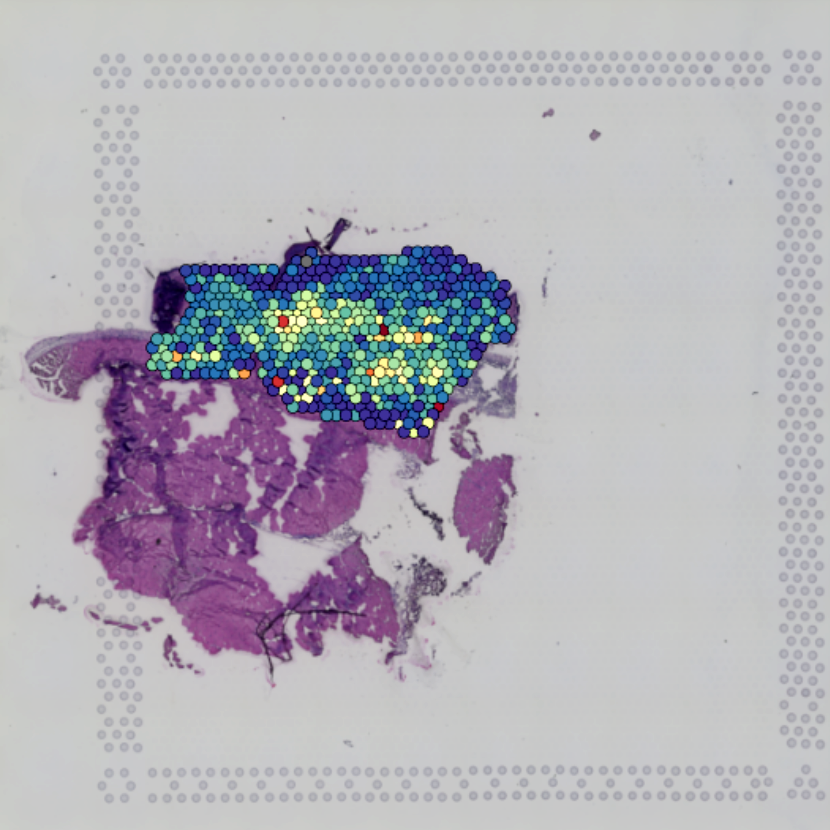

Supplement: Supplementary file 17 — Source data Fig. 4 [file 44319_2024_322_MOESM17_ESM.zip › SD figure 4/Figure4C/Macrophage.tif]

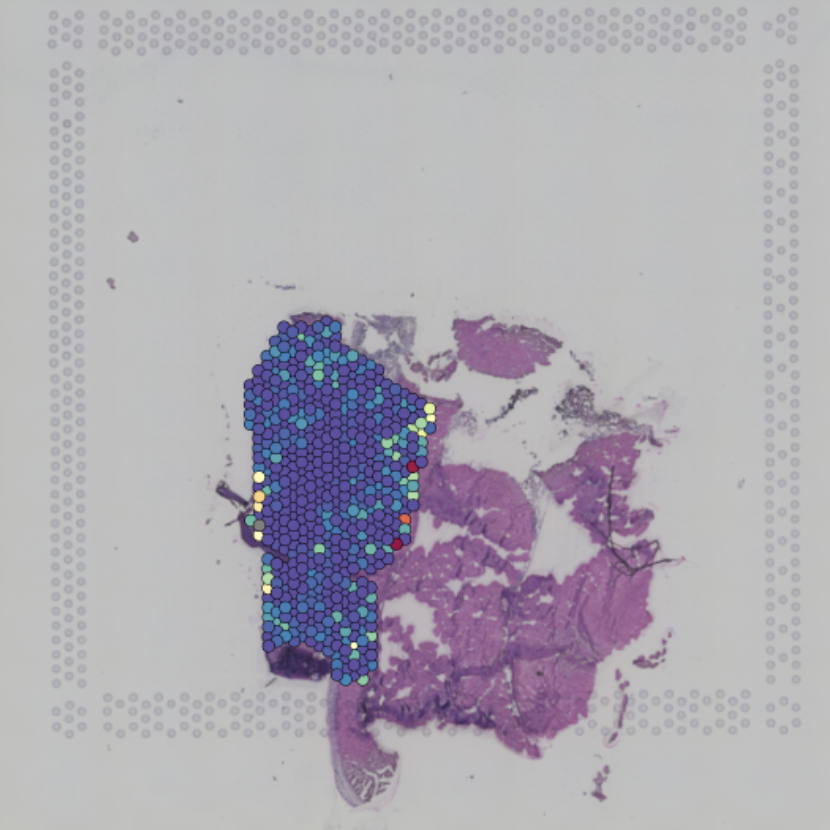

Supplement: Supplementary file 17 — Source data Fig. 4 [file 44319_2024_322_MOESM17_ESM.zip › SD figure 4/Figure4C/Neutophil.tif]

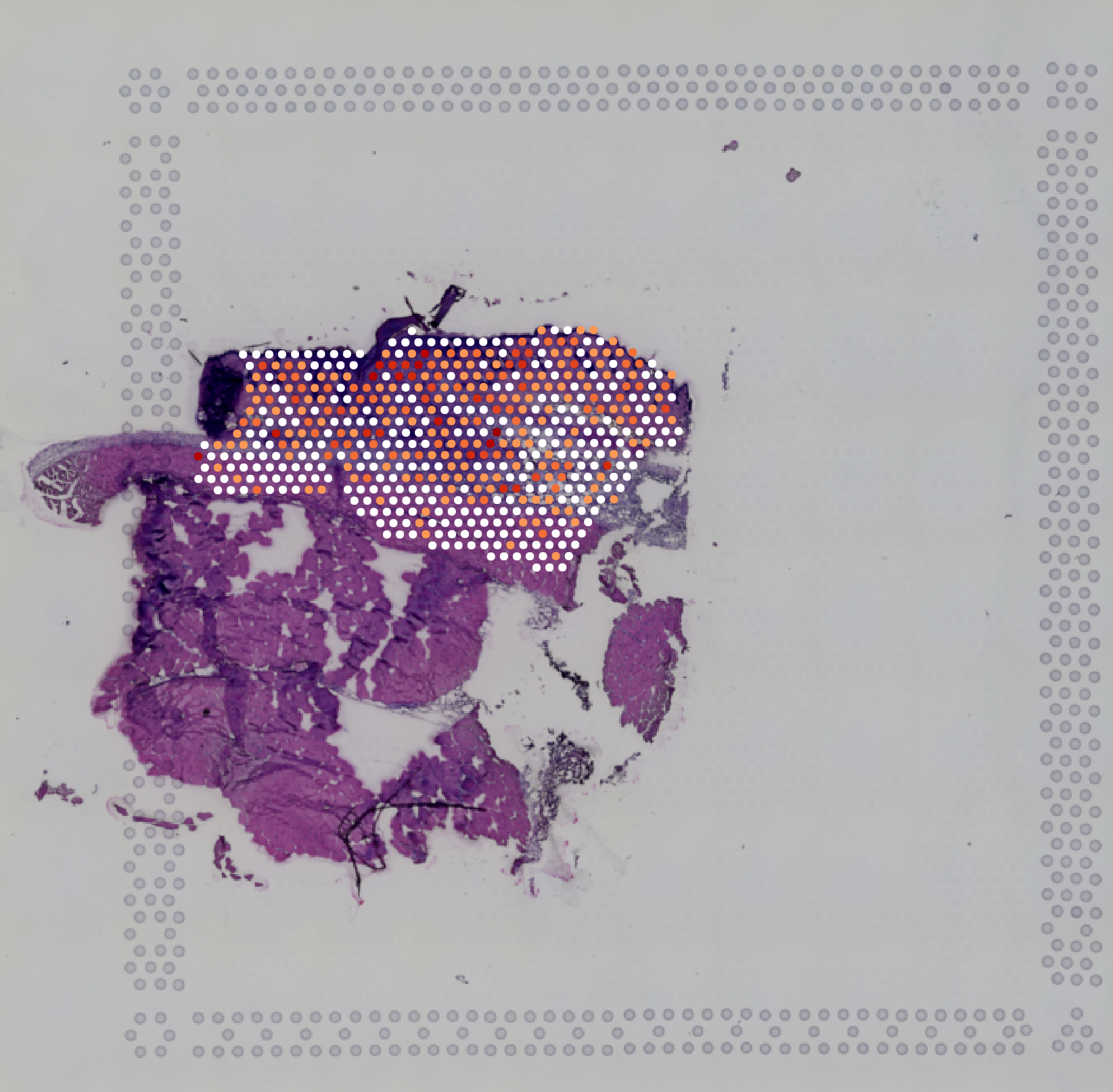

Supplement: Supplementary file 17 — Source data Fig. 4 [file 44319_2024_322_MOESM17_ESM.zip › SD figure 4/Figure4C/Pecam1.tif]

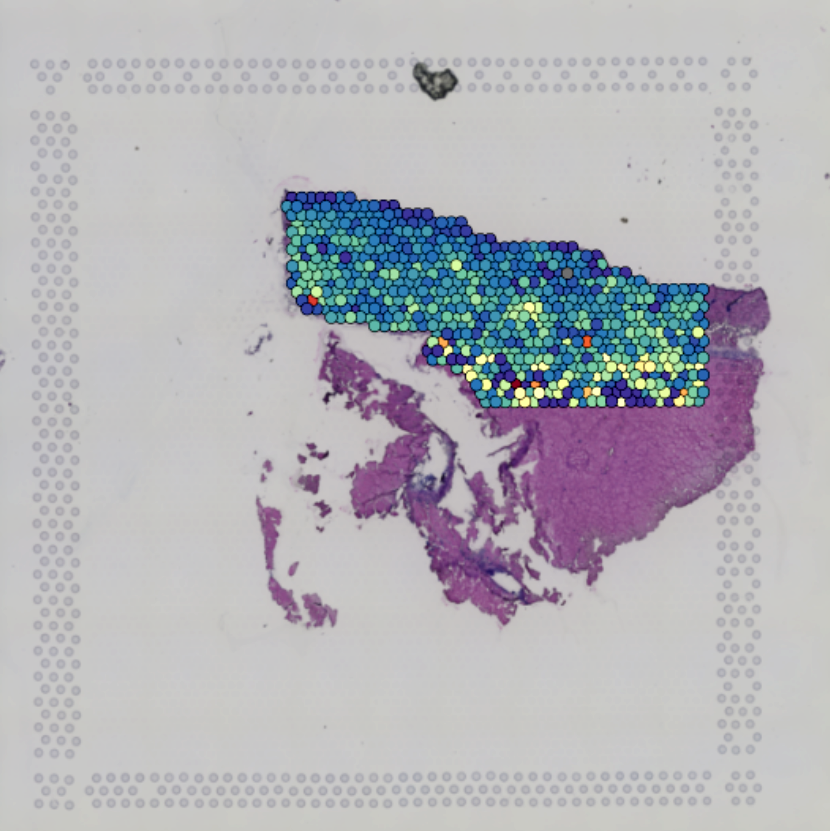

Supplement: Supplementary file 17 — Source data Fig. 4 [file 44319_2024_322_MOESM17_ESM.zip › SD figure 4/Figure4D/Endothelial cell.tif]

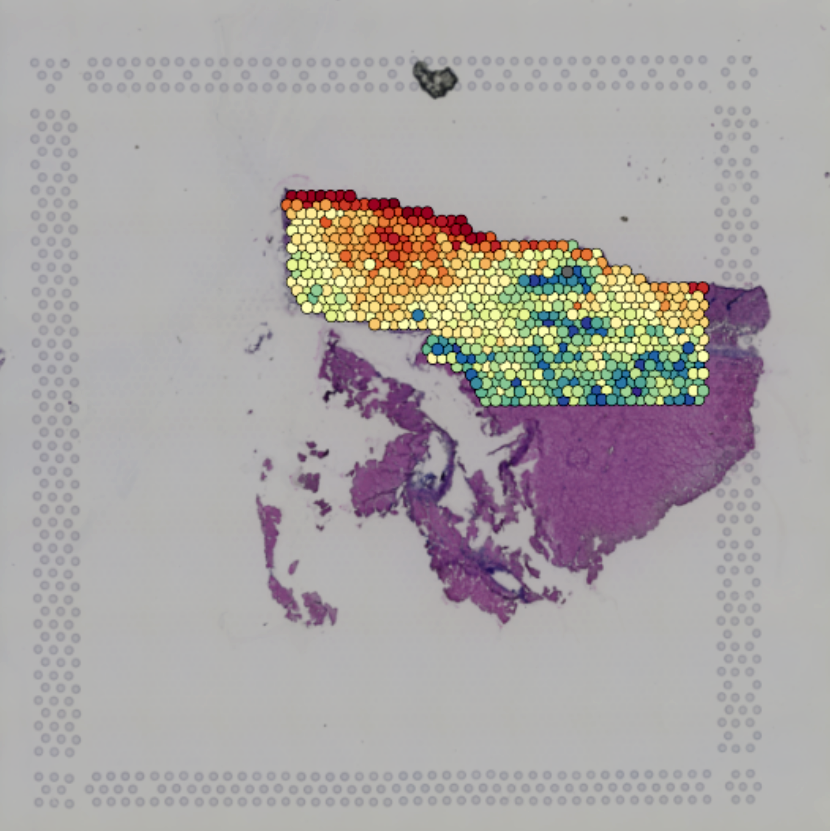

Supplement: Supplementary file 17 — Source data Fig. 4 [file 44319_2024_322_MOESM17_ESM.zip › SD figure 4/Figure4D/Epithelial cell.tif]

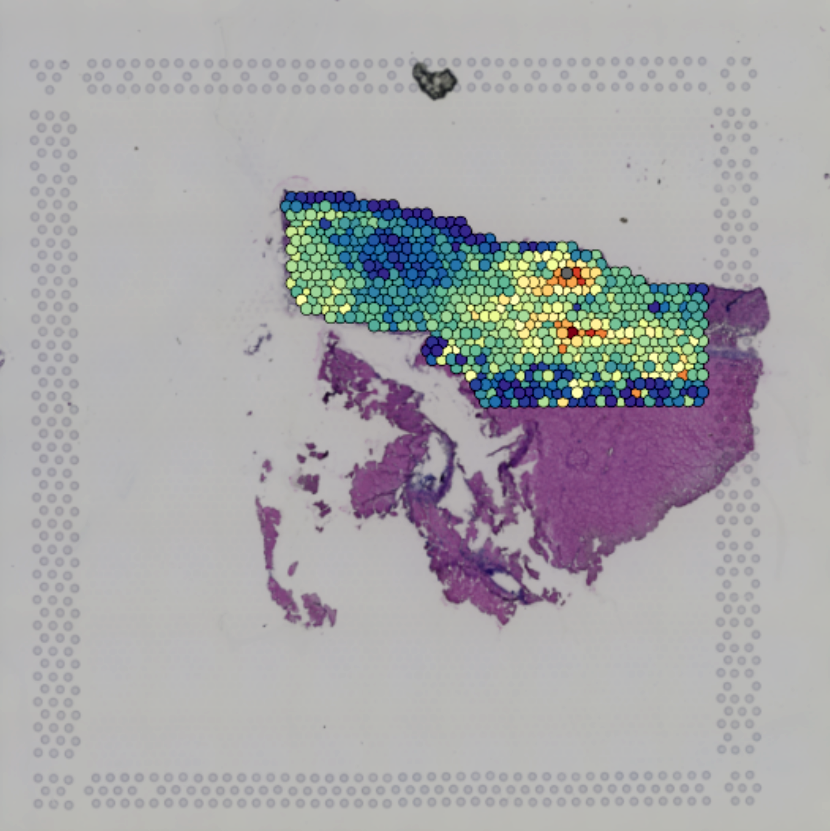

Supplement: Supplementary file 17 — Source data Fig. 4 [file 44319_2024_322_MOESM17_ESM.zip › SD figure 4/Figure4D/Fibroblast.tif]

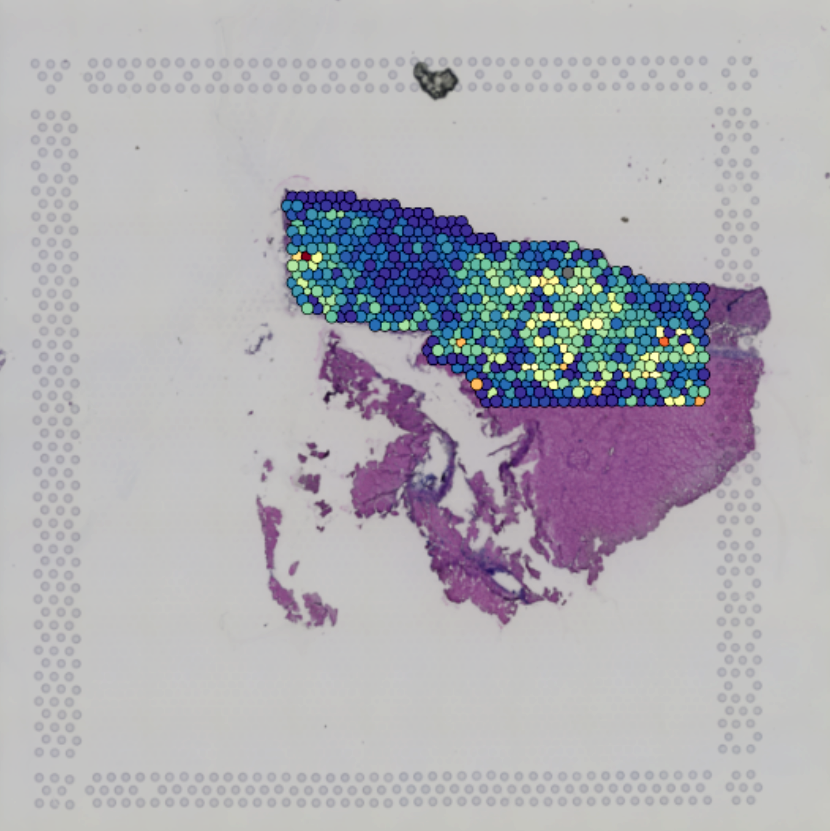

Supplement: Supplementary file 17 — Source data Fig. 4 [file 44319_2024_322_MOESM17_ESM.zip › SD figure 4/Figure4D/Macrophage.tif]

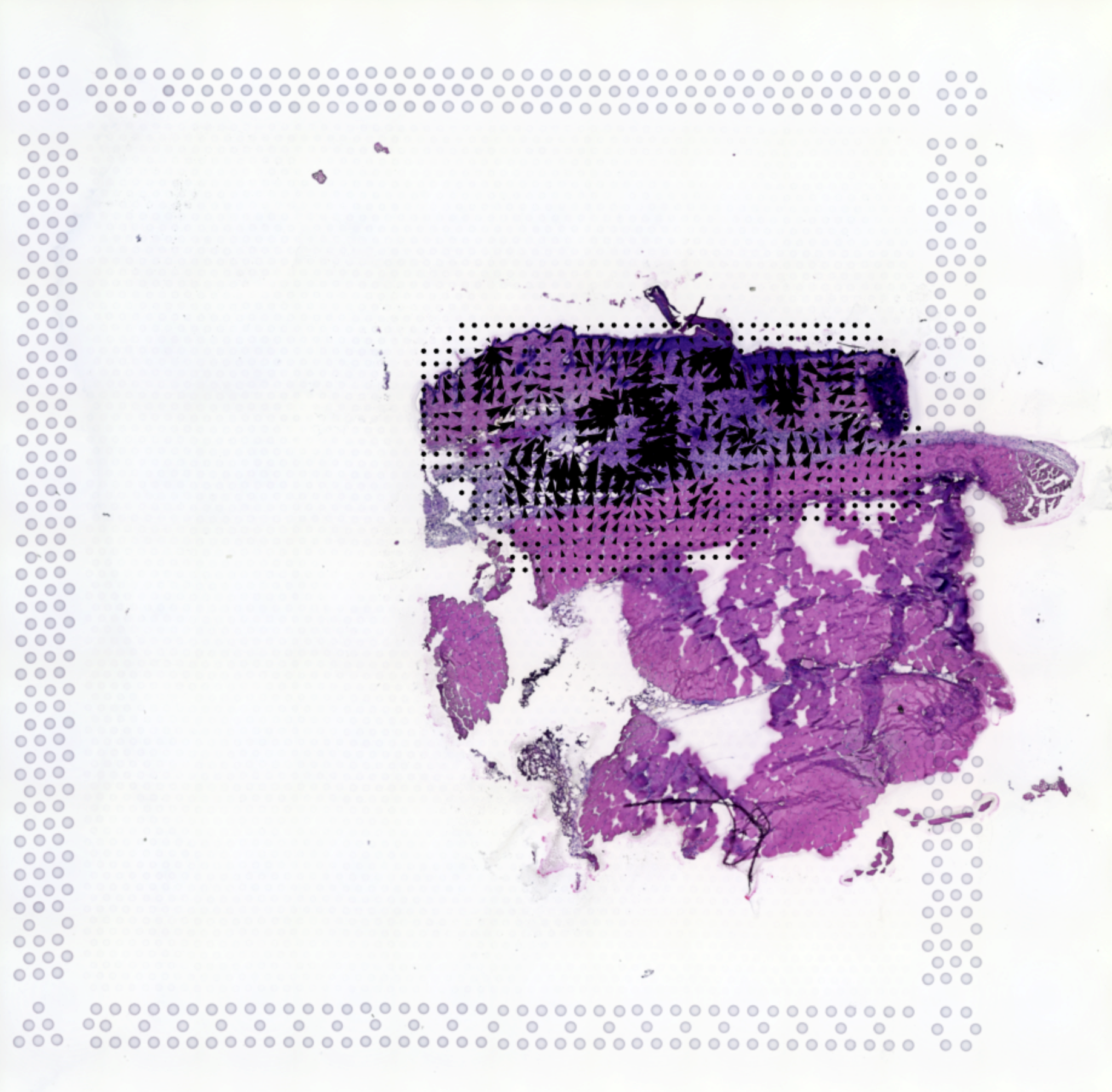

Supplement: Supplementary file 17 — Source data Fig. 4 [file 44319_2024_322_MOESM17_ESM.zip › SD figure 4/Figure4E/FN1.tif]

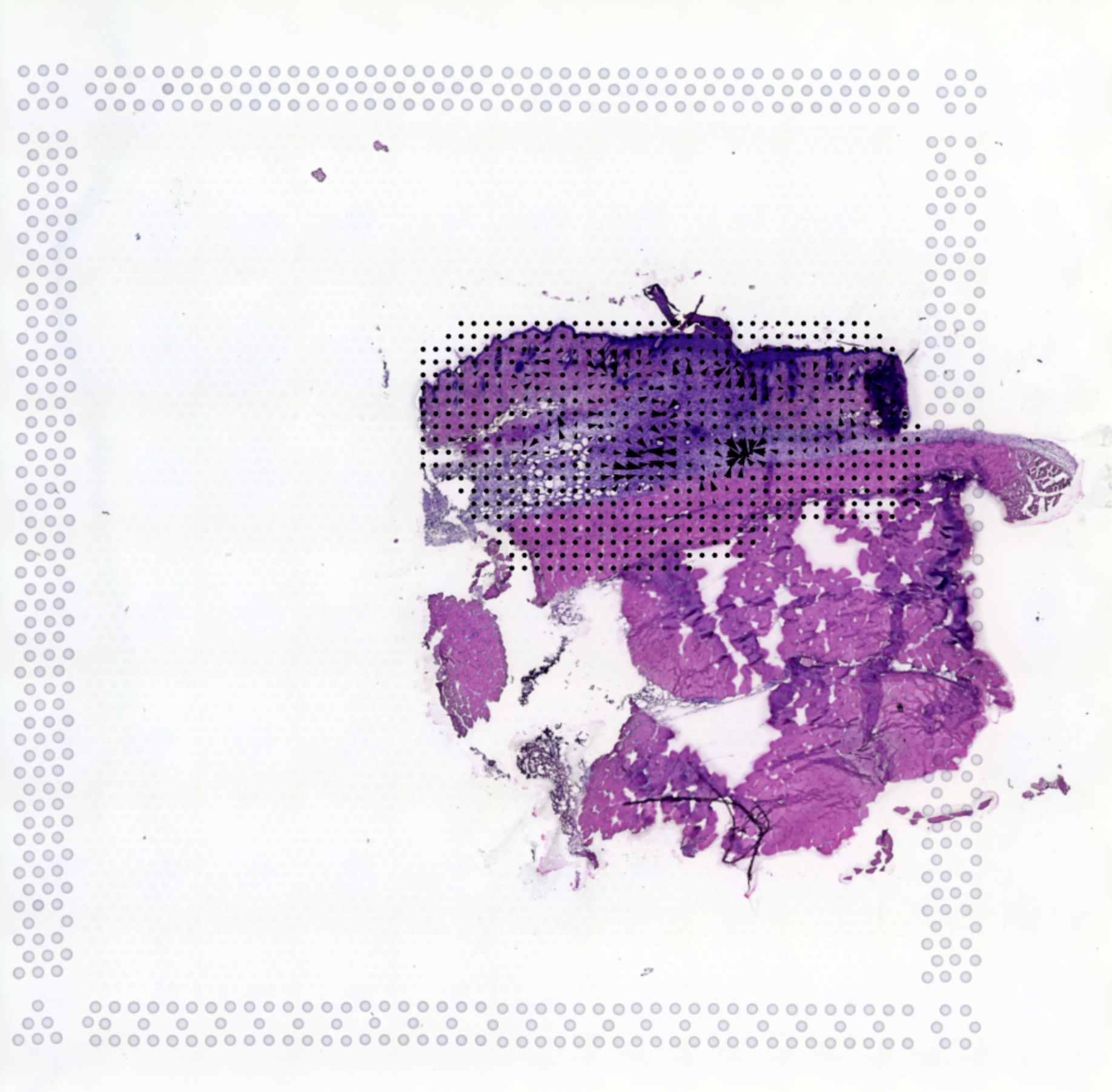

Supplement: Supplementary file 17 — Source data Fig. 4 [file 44319_2024_322_MOESM17_ESM.zip › SD figure 4/Figure4E/PERIOSTIN.tif]

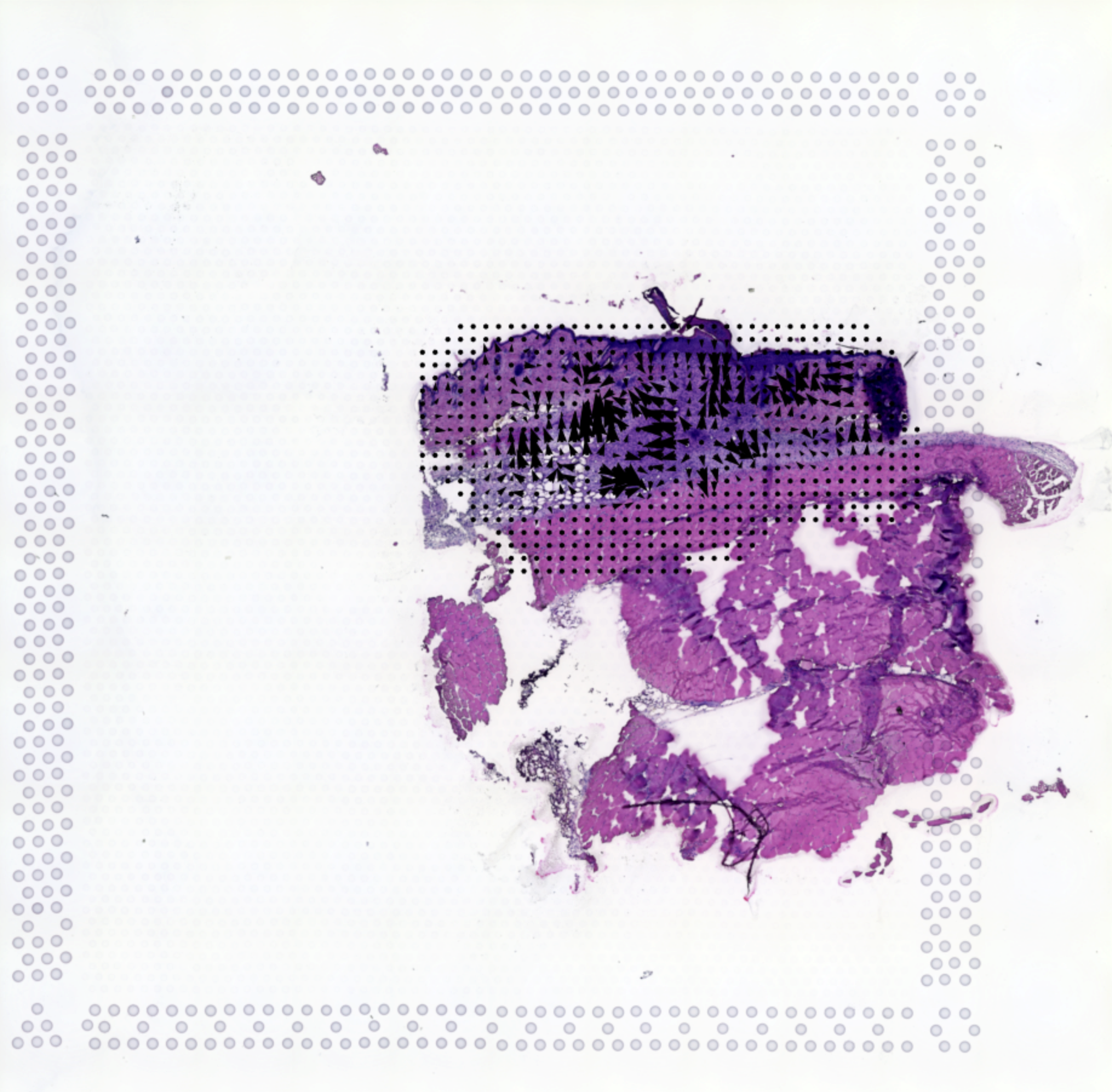

Supplement: Supplementary file 17 — Source data Fig. 4 [file 44319_2024_322_MOESM17_ESM.zip › SD figure 4/Figure4E/SPP1.tif]

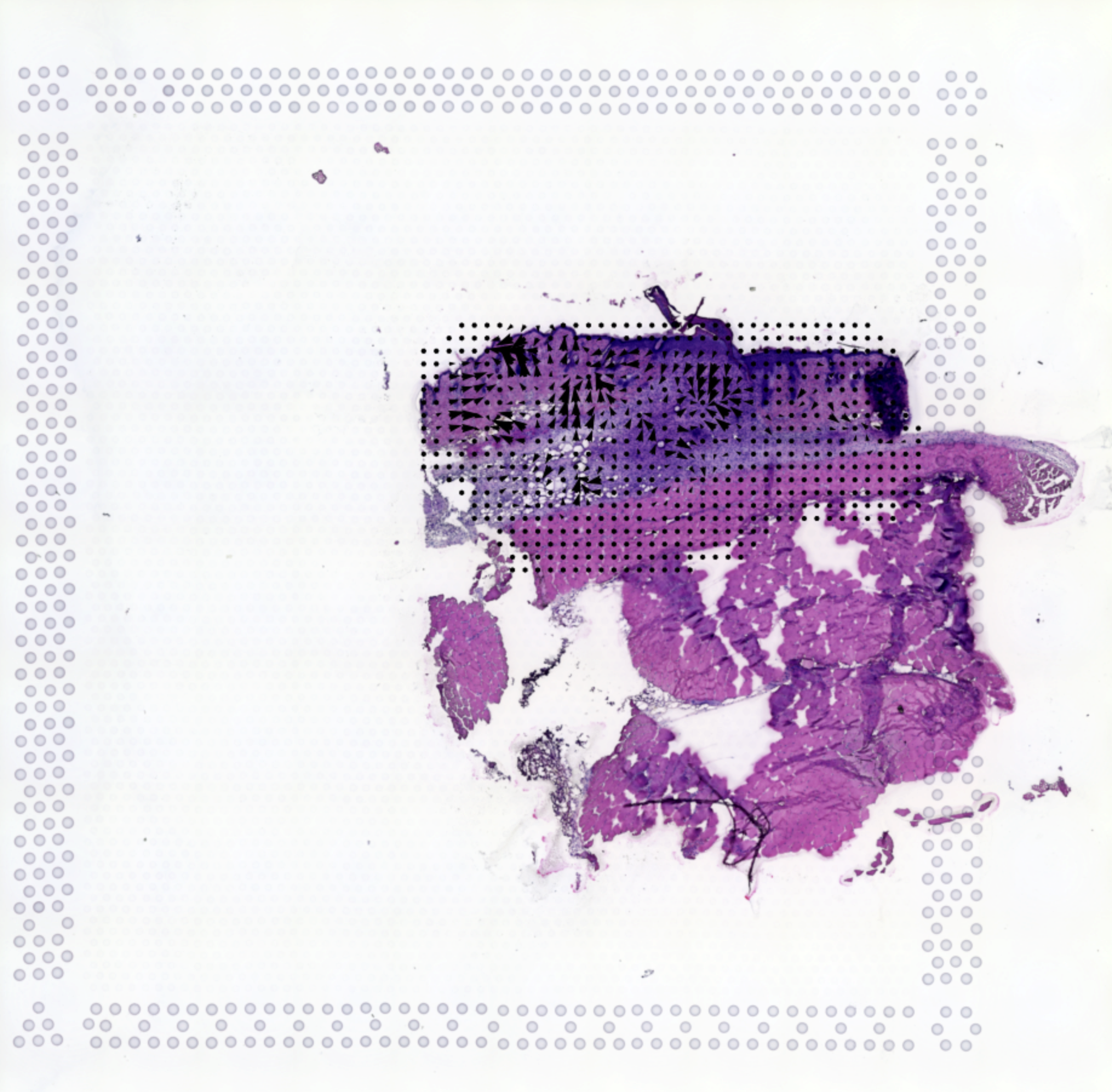

Supplement: Supplementary file 17 — Source data Fig. 4 [file 44319_2024_322_MOESM17_ESM.zip › SD figure 4/Figure4E/TGFb.tif]

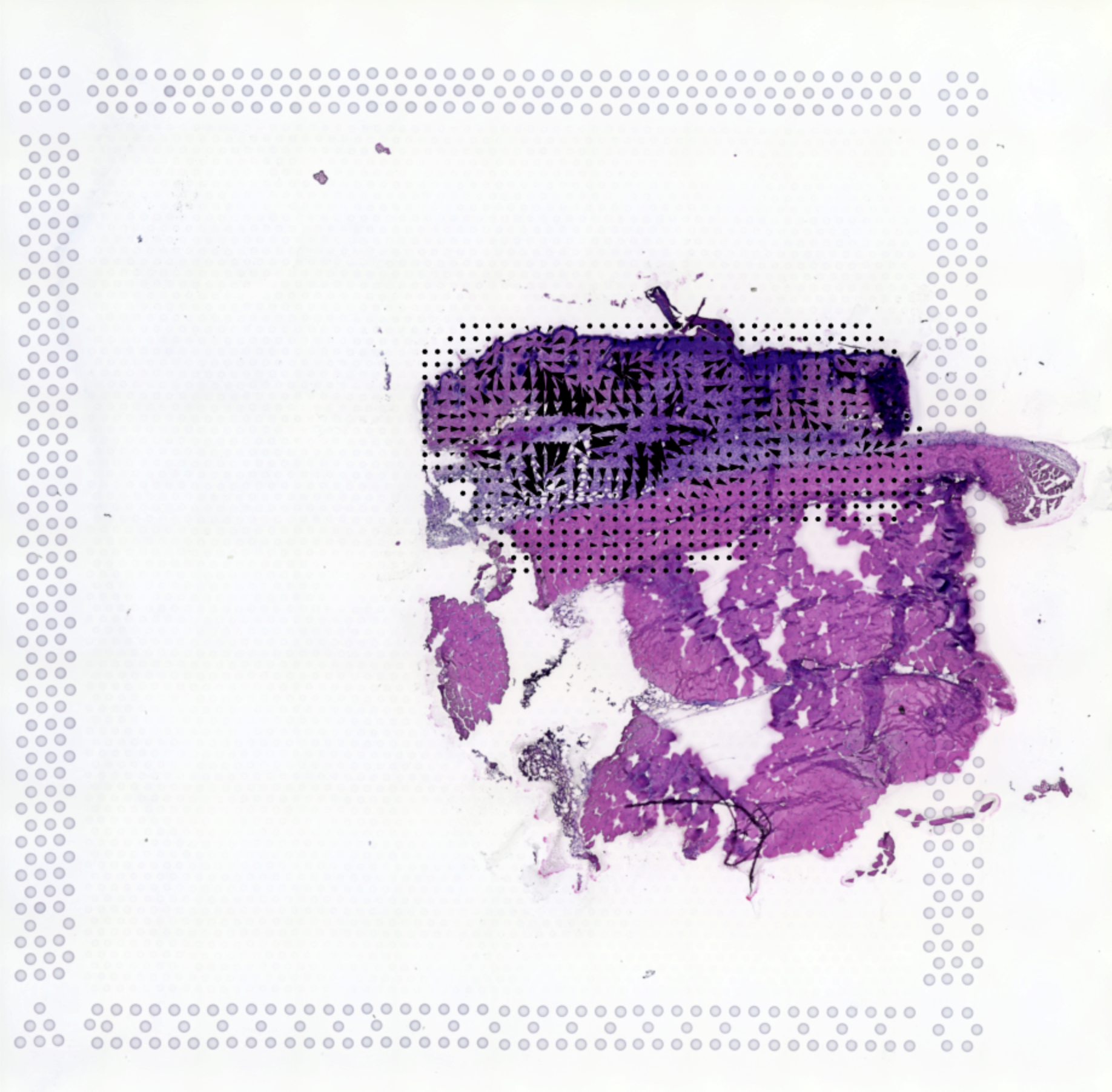

Supplement: Supplementary file 17 — Source data Fig. 4 [file 44319_2024_322_MOESM17_ESM.zip › SD figure 4/Figure4E/THBS.tif]

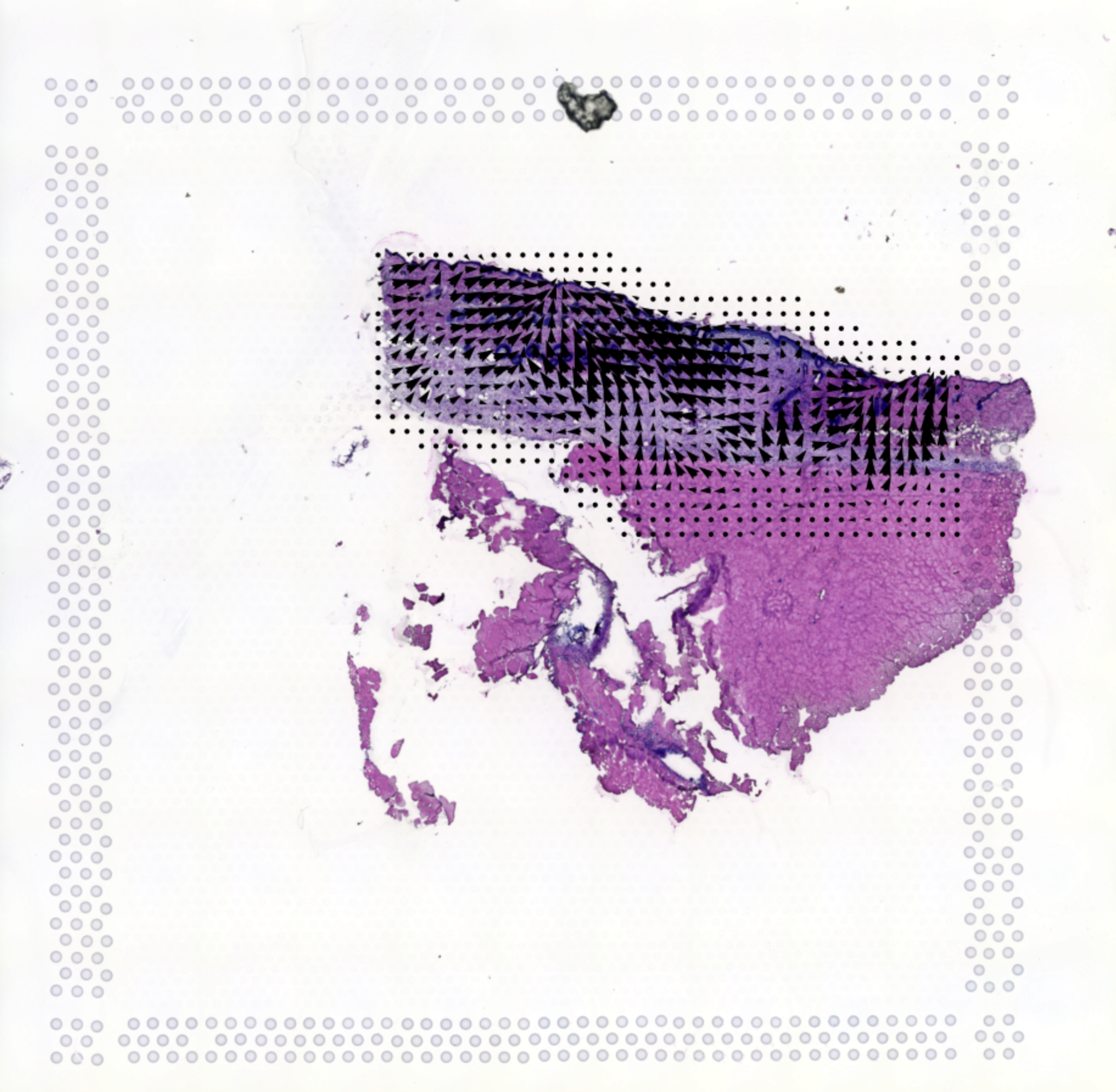

Supplement: Supplementary file 17 — Source data Fig. 4 [file 44319_2024_322_MOESM17_ESM.zip › SD figure 4/Figure4F/FN1.tif]

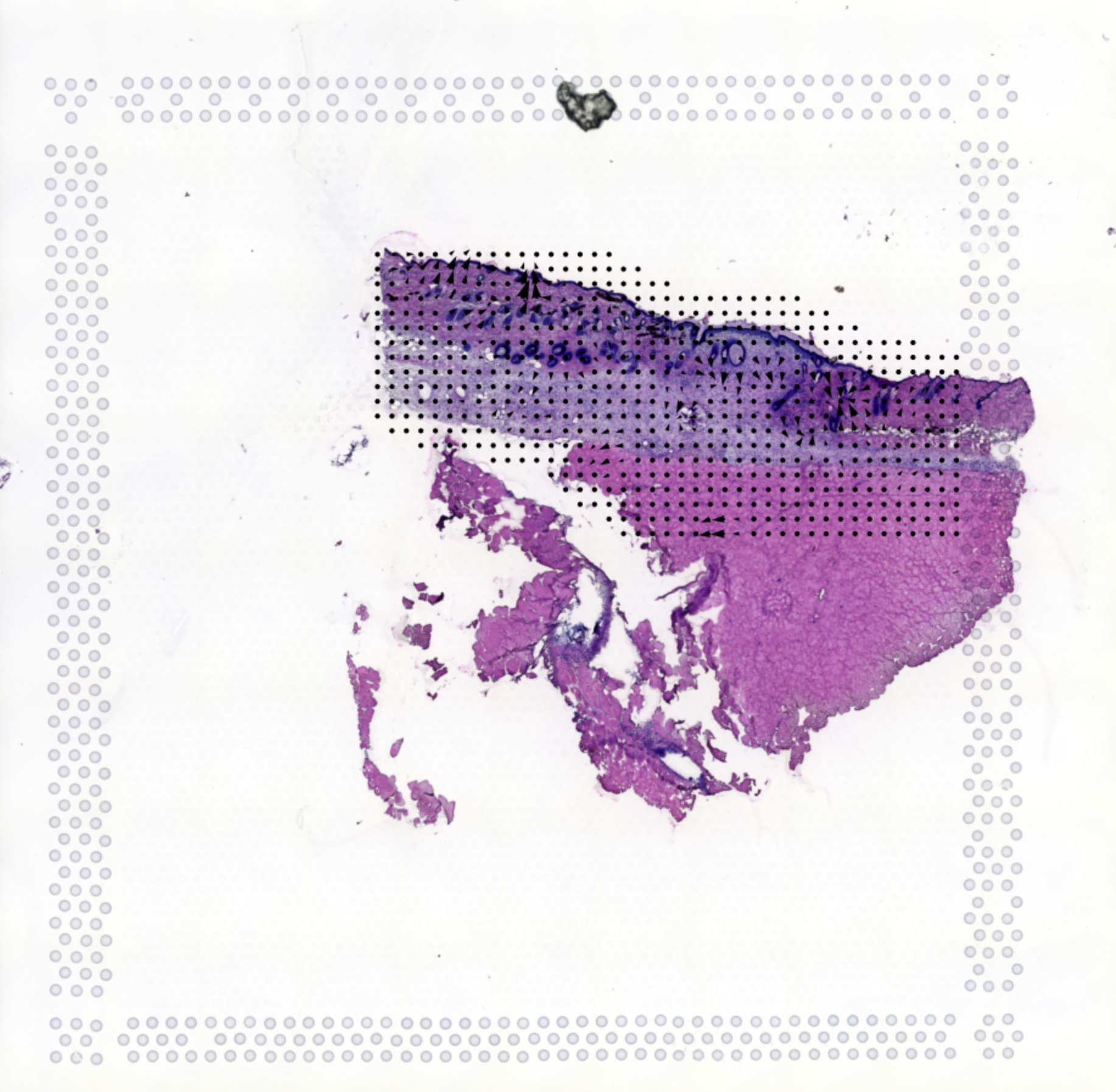

Supplement: Supplementary file 17 — Source data Fig. 4 [file 44319_2024_322_MOESM17_ESM.zip › SD figure 4/Figure4F/PERIOSTIN.tif]

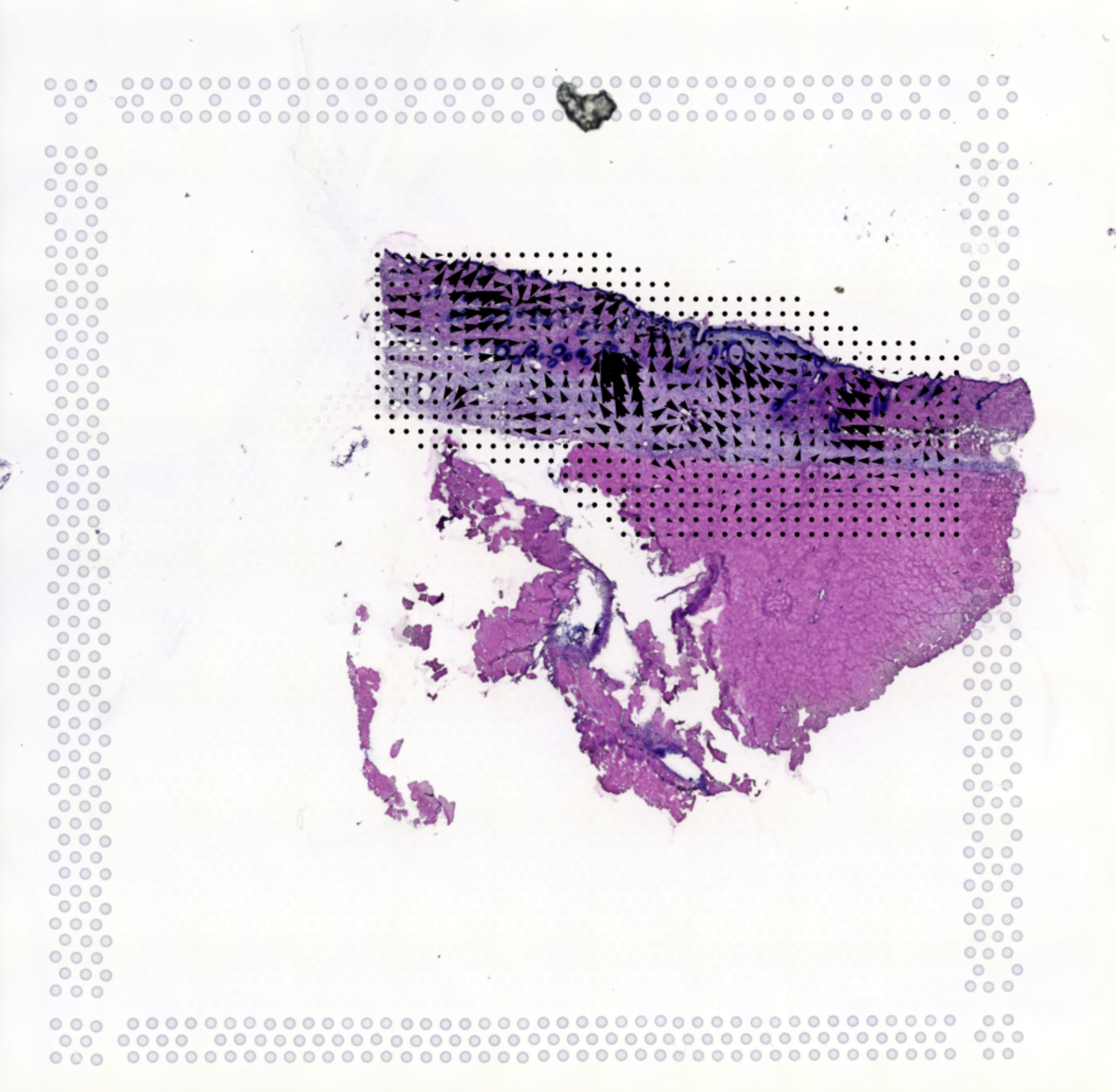

Supplement: Supplementary file 17 — Source data Fig. 4 [file 44319_2024_322_MOESM17_ESM.zip › SD figure 4/Figure4F/SPP1.tif]

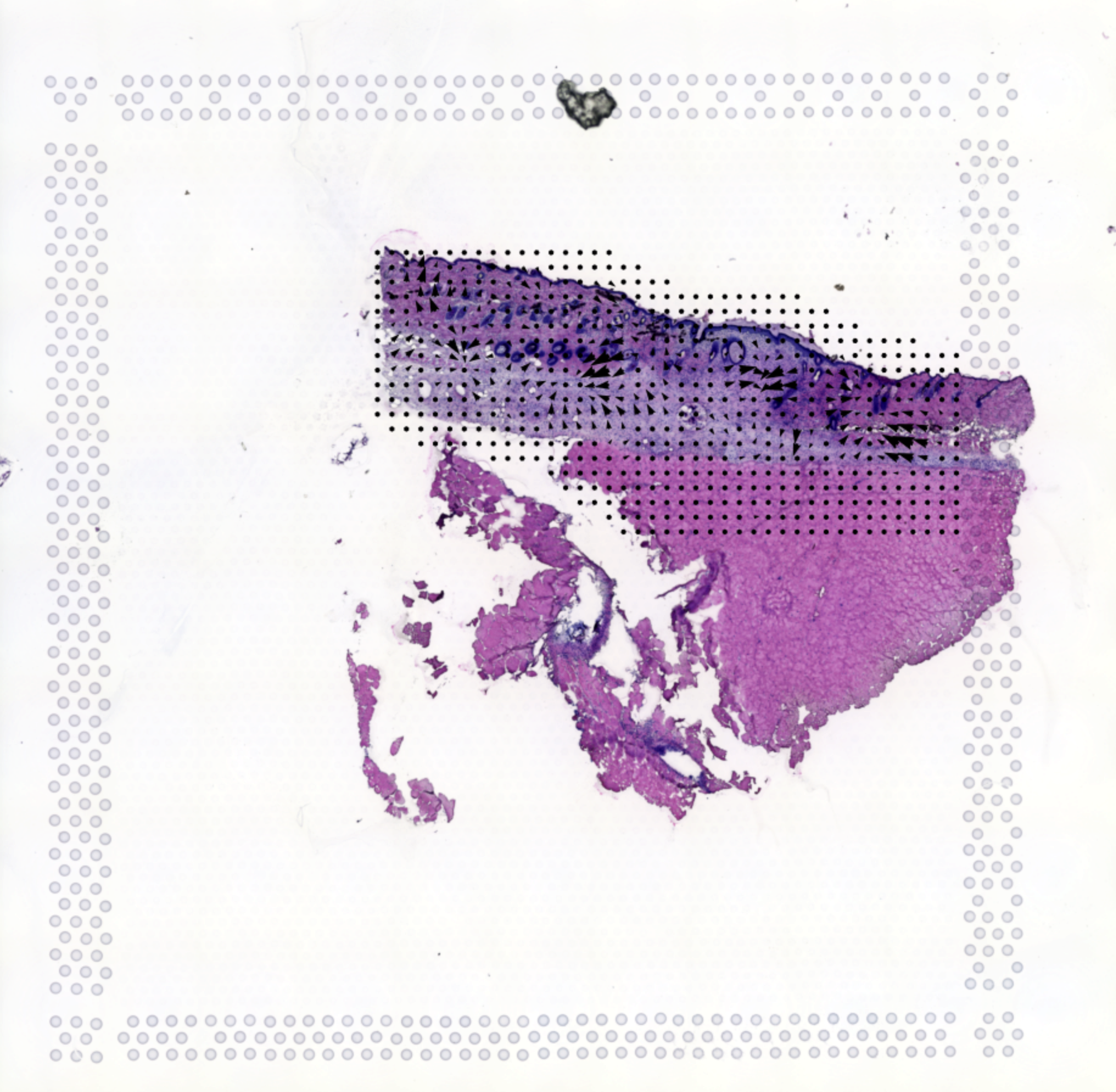

Supplement: Supplementary file 17 — Source data Fig. 4 [file 44319_2024_322_MOESM17_ESM.zip › SD figure 4/Figure4F/TGFb.tif]

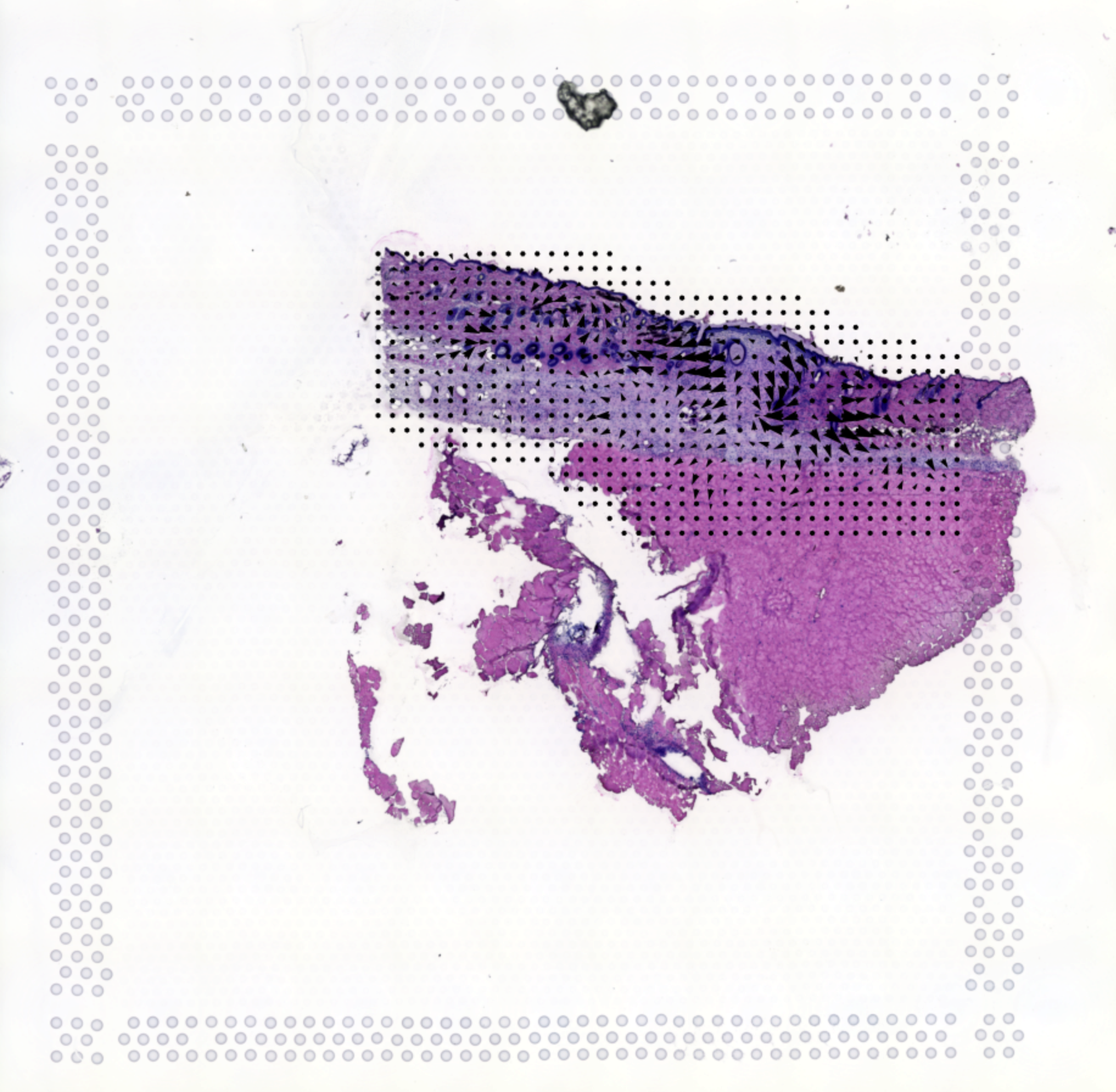

Supplement: Supplementary file 17 — Source data Fig. 4 [file 44319_2024_322_MOESM17_ESM.zip › SD figure 4/Figure4F/THBS.tif]
