# Supplementary material for: Novel integrated multiomics analysis reveals a key role for integrin beta-like 1 in wound scarring
Source: EMBO Rep. 2024 Nov 18;26(1):122–52. doi: 10.1038/s44319-024-00322-3 (PMC11724056; doi:10.1038/s44319-024-00322-3)
Supplement: Supplementary file 19 — Expanded View Figures [file 44319_2024_322_MOESM19_ESM.pdf]

Expanded View Figures

Figure EV1. Identification of top ten marker genes in the macrophage subclusters.

Bubble heatmap of macrophage subclusters (M1–M6) and their respective marker genes.

 © The Author(s)

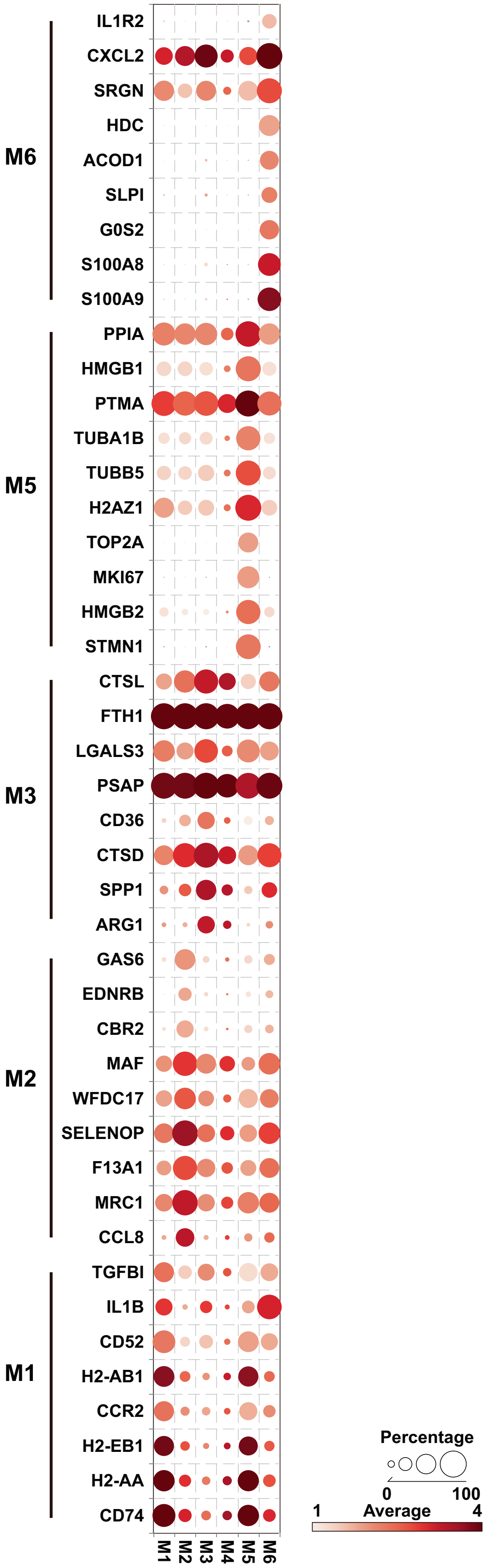
M6
IL1R2
CXCL2
SRGN
HDC
ACOD1
SLPI
G0S2
S100A8
S100A9
M5
PPIA
HMGB1
PTMA
TUBA1B
TUBB5
H2AZ1
TOP2A
MKI67
HMGB2
STMN1
M3
CTSL
FTH1
LGALS3
PSAP
CD36
CTSD
SPP1
ARG1
M2
GAS6
EDNRB
CBR2
MAF
WFDC17
SELENOP
F13A1
MRC1
CCL8
M1
TGFBI
IL1B
CD52
H2-AB1
CCR2
H2-EB1
H2-AA
CD74
M1
M2
M3
M4
M5
M6
Percentage
0
100
Average
1
4

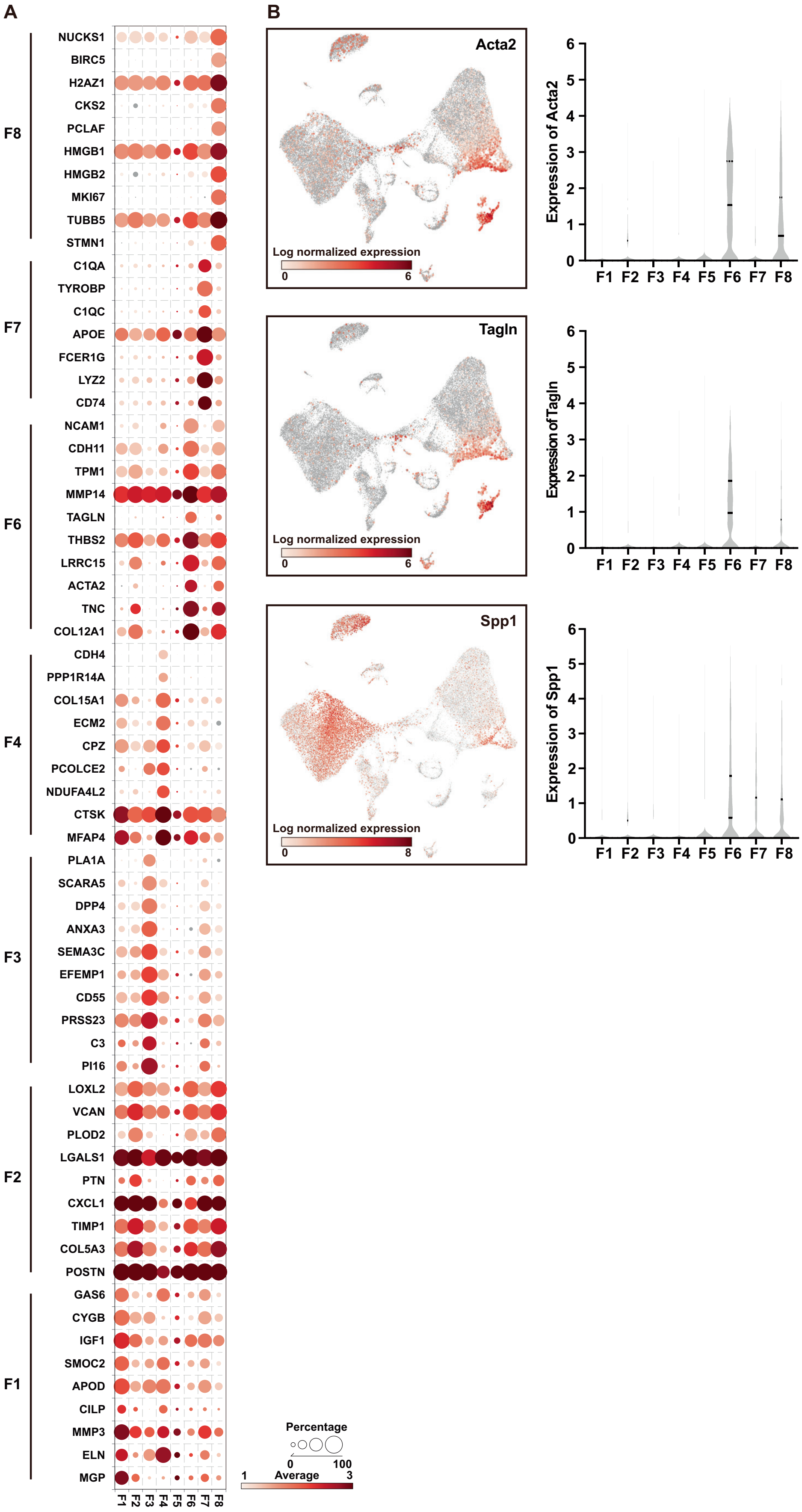
A
F8
NUCKS1
BIRC5
H2AZ1
CKS2
PCLAF
HMGB1
HMGB2
MKI67
TUBB5
STMN1
F7
C1QA
TYROBP
C1QC
APOE
FCER1G
LYZ2
CD74
F6
NCAM1
CDH11
TPM1
MMP14
TAGLN
THBS2
LRRC15
ACTA2
TNC
COL12A1
F4
CDH4
PPP1R14A
COL15A1
ECM2
CPZ
PCOLCE2
NDUFA4L2
CTSK
MFAP4
F3
PLA1A
SCARA5
DPP4
ANXA3
SEMA3C
EFEMP1
CD55
PRSS23
C3
PI16
F2
LOXL2
VCAN
PLOD2
LGALS1
PTN
CXCL1
TIMP1
COL5A3
POSTN
F1
GAS6
CYGB
IGF1
SMOC2
APOD
CILP
MMP3
ELN
MGP
F1 F2 F3 F4 F5 F6 F7 F8
Percentage
0 100
Average
1 3
B
Acta2
Log normalized expression
0 6
Expression of Acta2
Tagln
Log normalized expression
0 6
Expression of Tagln
Spp1
Log normalized expression
0 8
Expression of Spp1
0 1 2 3 4 5 6
F1 F2 F3 F4 F5 F6 F7 F8

Figure EV2. Identification of top 10 marker genes in the fibroblast subclusters.

(**A**) Bubble heatmap of fibroblast subclusters (F1–F8) and their respective marker genes. (**B**) UMAP plot (left) of expression levels of *Acta2* (top), *Tagln* (middle), and *Spp1* (bottom) across 40,024 cells from Days 3, 7, and 14 post injury (left panel). Violin plot (right) showing expression levels of these genes in each fibroblast subcluster. F1: $n = 4265$; F2: $n = 3849$; F3: $n = 2218$; F4: $n = 1421$; F5: $n = 1369$; F6: $n = 977$; F7: $n = 804$; F8: $n = 532$.

© The Author(s)

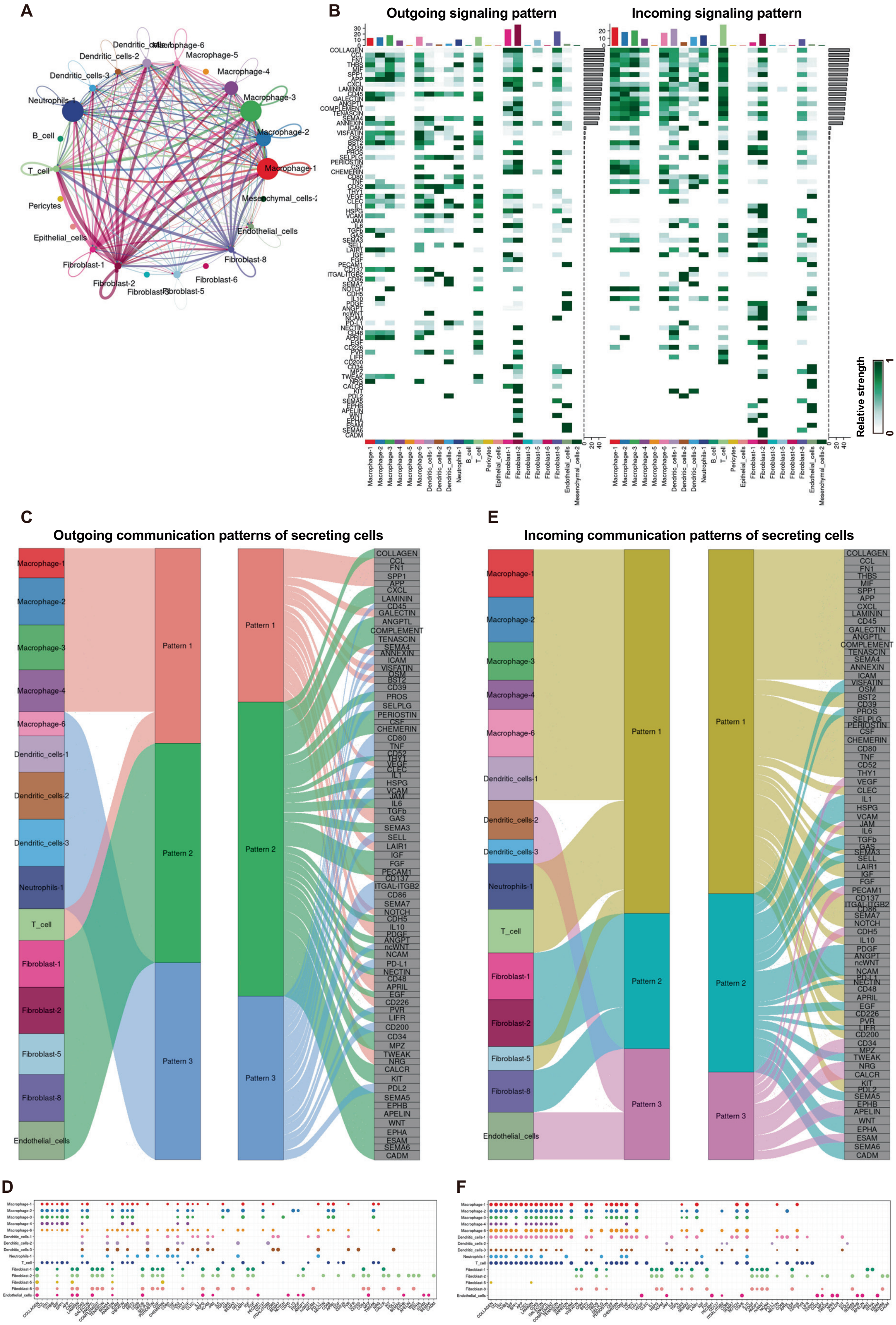
A
B
Outgoing signaling pattern
Incoming signaling pattern
Relative strength
C
Outgoing communication patterns of secreting cells
Pattern 1
Pattern 2
Pattern 3
D
E
Incoming communication patterns of secreting cells
F

Figure EV3. Inference with the cell–cell communication network on Day 3 post injury.

(**A**) Circle plot of signaling pathways. (**B**) Heatmap analysis of the roles of the representative signaling pathways in the aggregated cell–cell communication network. (**C**) Alluvial plot of the outgoing signaling patterns of secreting cells, demonstrating the correspondence between the inferred latent pattern and cell groups, as well as the signaling pathways. The thickness of the flow indicates the contribution of the cell group or signaling pathway to each latent pattern. The height of each pattern is proportional to the number of its associated cell groups or signaling pathways. Outgoing patterns reveal how the sender cells coordinate with each other, as well as how they coordinate with certain signaling pathways to drive communications. (**D**) Bobble plot of the outgoing signaling patterns of secreting cells. (**E**) Incoming signaling patterns of target cells, showing how the target cells coordinate with each other and with certain signaling pathways to respond to incoming signals. (**F**) Bobble plot of the incoming signaling patterns of target cells.

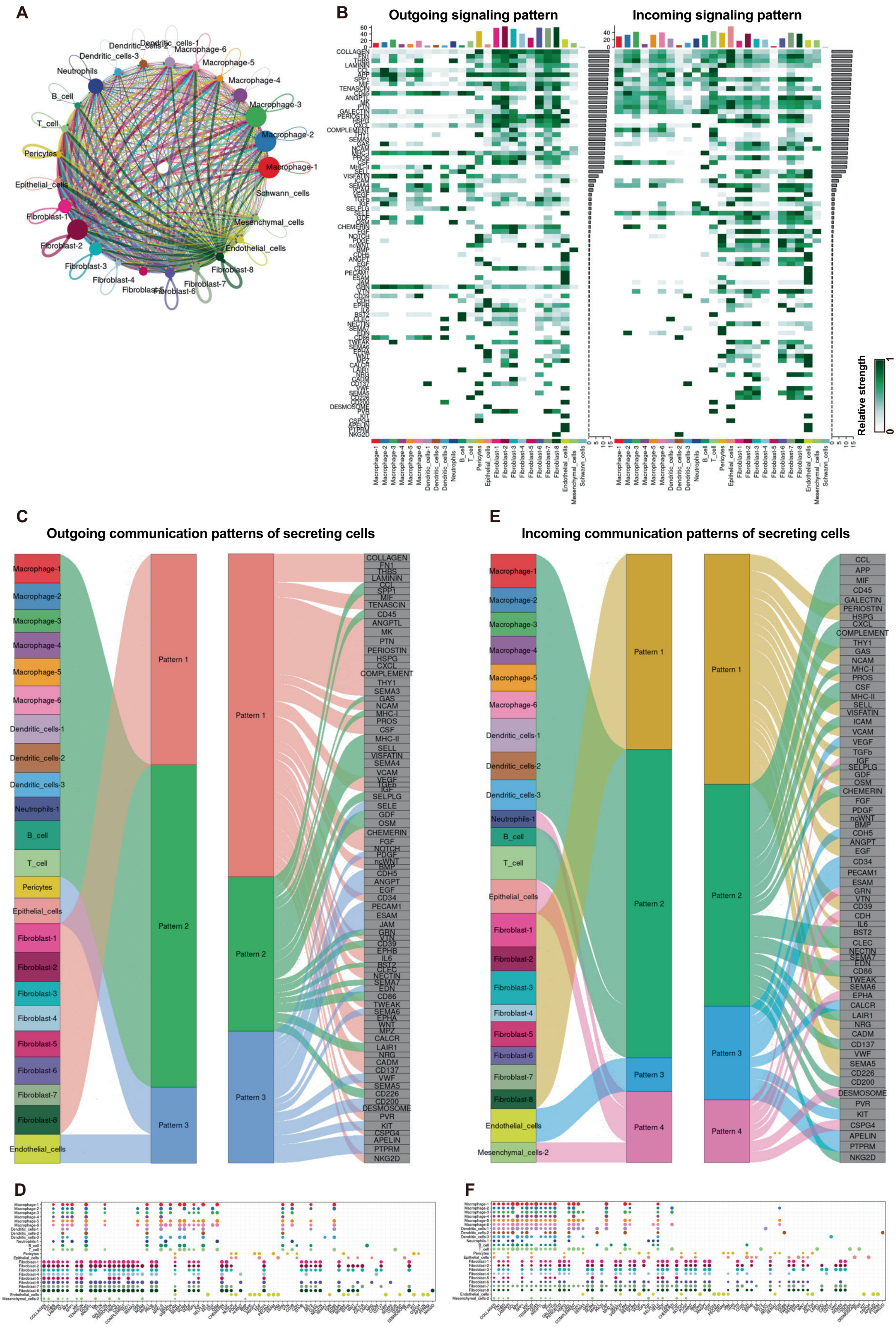
A
B
Outgoing signaling pattern
Incoming signaling pattern
Relative strength
C
Outgoing communication patterns of secreting cells
Pattern 1
Pattern 2
Pattern 3
E
Incoming communication patterns of secreting cells
Pattern 1
Pattern 2
Pattern 3
Pattern 4
D
F

Figure EV4. Inference with the cell–cell communication network on Day 7 post injury.

(**A**) Circle plot of signaling pathways. (**B**) Heatmap analysis of the roles of the representative signaling pathways in the aggregated cell–cell communication network. (**C**) Alluvial plot of the outgoing signaling patterns of secreting cells, demonstrating the correspondence between the inferred latent pattern and cell groups, as well as the signaling pathways. The thickness of the flow indicates the contribution of the cell group or signaling pathway to each latent pattern. The height of each pattern is proportional to the number of its associated cell groups or signaling pathways. Outgoing patterns reveal how the sender cells coordinate with each other, as well as how they coordinate with certain signaling pathways to drive communications. (**D**) Bobble plot of the outgoing signaling patterns of secreting cells. (**E**) Incoming signaling patterns of target cells, showing how the target cells coordinate with each other and with certain signaling pathways to respond to incoming signals. (**F**) Bobble plot of the incoming signaling patterns of target cells.

© The Author(s)

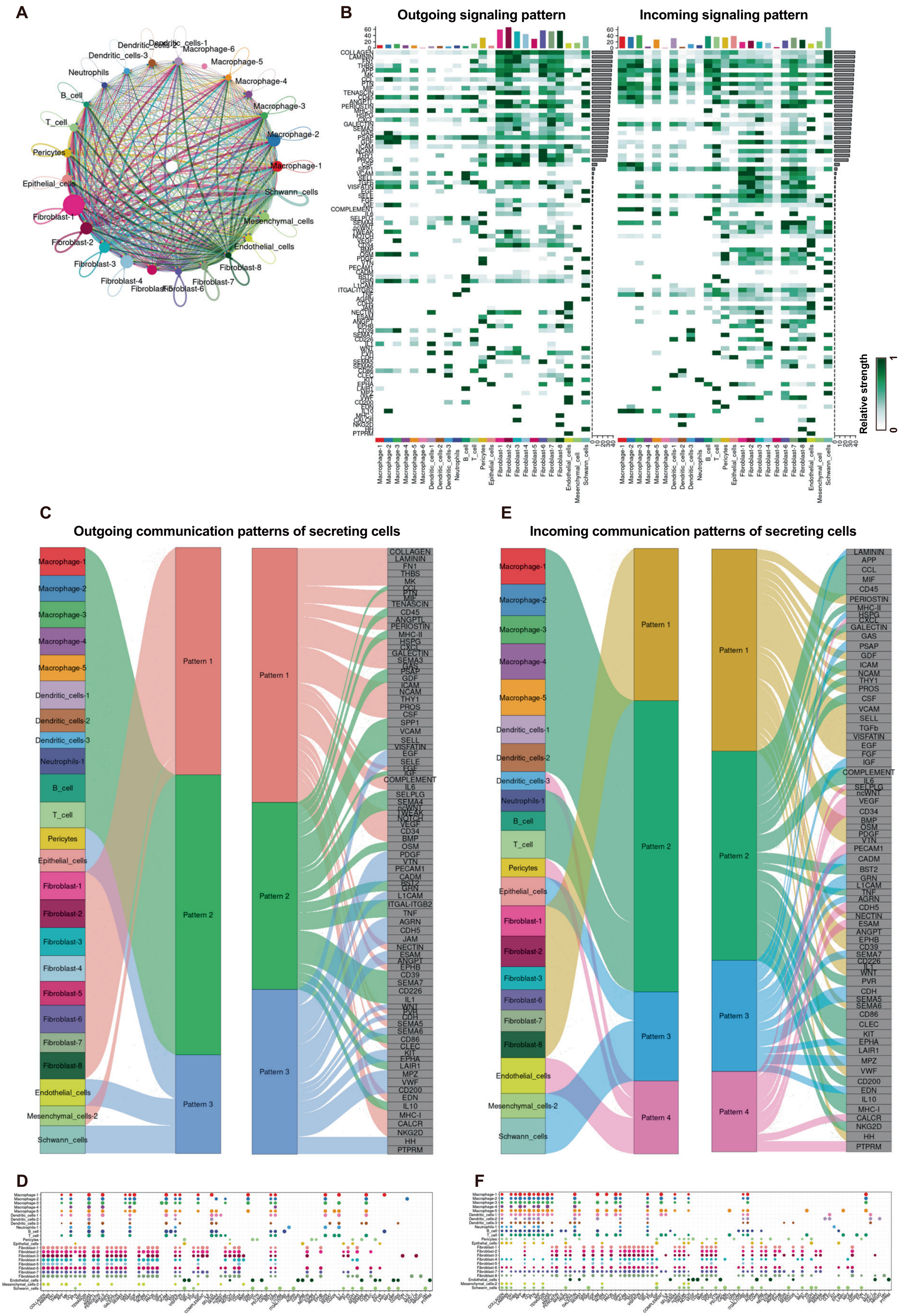
A
B
Outgoing signaling pattern
Incoming signaling pattern
Relative strength
C
Outgoing communication patterns of secreting cells
E
Incoming communication patterns of secreting cells
D
F

Figure EV5. Inference with the cell–cell communication network on Day 14 post injury.

(**A**) Circle plot of signaling pathways. (**B**) Heatmap analysis of the roles of the representative signaling pathways in the aggregated cell–cell communication network. (**C**) Alluvial plot of the outgoing signaling patterns of secreting cells, demonstrating the correspondence between the inferred latent pattern and cell groups, as well as the signaling pathways. The thickness of the flow indicates the contribution of the cell group or signaling pathway to each latent pattern. The height of each pattern is proportional to the number of its associated cell groups or signaling pathways. Outgoing patterns reveal how the sender cells coordinate with each other, as well as how they coordinate with certain signaling pathways to drive communications. (**D**) Bobble plot of the outgoing signaling patterns of secreting cells. (**E**) Incoming signaling patterns of target cells, showing how the target cells coordinate with each other and with certain signaling pathways to respond to incoming signals. (**F**) Bobble plot of the incoming signaling patterns of target cells.

© The Author(s)
